# Endophytic Microbes from Medicinal Plants in Fenghuang Mountain as a Source of Antibiotics

**DOI:** 10.3390/molecules28176301

**Published:** 2023-08-28

**Authors:** Aiping Yang, Yu Hong, Fengjuan Zhou, Ling Zhang, Youjuan Zhu, Chang Wang, Yang Hu, Li Yu, Lihong Chen, Xiachang Wang

**Affiliations:** 1 College of Pharmacy, Nanjing University of Chinese Medicine, Nanjing 210023, China; 2 Fujian Province Key Laboratory for the Development of Bioactive Material from Marine Algae, College of Oceanology and Food Science, Quanzhou Normal University, Quanzhou 362000, China; 3 Level 3 Laboratory of Molecular Biology (Epidemic and Febrile Diseases) of National TCM Administrator, Nanjing University of Chinese Medicine, Nanjing 210023, China

**Keywords:** antibiotics, endophyte, medicinal plant, strain diversity, *Streptomyces*, *Penicillium*

## Abstract

One of the largest concerns with world health today is still antibiotic resistance, which is making it imperative to find efficient alternatives as soon as possible. It has been demonstrated that microbes are reliable sources for the creation of therapeutic antibiotics. This research intends to investigate the endophytic microorganisms from several medicinal plants in Fenghuang Mountain (Jiangsu Province, China) and to discover new antibiotics from their secondary metabolites. A total of 269 endophytic strains were isolated from nine distinct medicinal plants. Taxonomic analysis revealed that there were 20 distinct species among these endophytes, with *Streptomyces* being the most common genus. Three of the target strains were chosen for scale-up fermentation after preliminary screening of antibacterial activities and the metabolomics investigation using LC-MS. These strains were *Penicillium* sp. NX-S-6, *Streptomyces* sp. YHLB-L-2 and *Streptomyces* sp. ZLBB-S-6. Twenty-three secondary metabolites (**1**–**23**), including a new sorbicillin analogue (**1**), were produced as a result of antibacterial activity-guided isolation. Through spectroscopic analysis using MS and NMR, the structures of yield compounds were clarified. According to antibacterial data, *S. aureus* or *B. subtilis* were inhibited to varying degrees by sorrentanone (**3**), emodic acid (**8**), GKK1032 B (**10**), linoleic acid (**14**), toyocamycin (**17**) and quinomycin A (**21**). The most effective antimicrobial agent against *S. aureus*, *B. subtilis*, *E. coli* and *A. baumannii* was quinomycin A (**21**). In addition, quinomycin A showed strong antifungal activity against *Aspergillus fumigatus, Cryptococcus neoformans,* and two clinical isolated strains *Aspergillus fumigatus* #176 and #339, with MIC as 16, 4, 16 and 16 µg/mL, respectively. This is the first time that bioprospecting of actinobacteria and their secondary metabolites from medicinal plants in Fenghuang Mountain was reported. The finding demonstrates the potential of endophytic microbes in medical plants to produce a variety of natural products. Endophytic microbes will be an important source for new antibiotics.

## 1. Introduction

Currently, antibiotic resistance is a significant issue that needs to be tackled globally [1]. Researchers have had to redouble their efforts to find additional novel antibiotics in order to look for more therapeutic possibilities. New antibiotic agents can be derived from natural products, as has been demonstrated [2]. Microorganisms are the most reproducible and long-lasting producers of bioactive compounds, and endophytic microbes are thought to be an important component [3]. After the isolation of taxol from an endophytic fungus in Pacific yew [4], endophytes have emerged as a valuable source for the discovery of bioactive natural products [5,6], as they were found to produce bioactivity-related secondary metabolites in a manner similar to that of the hosts [7]. The majority of endophyte research has concentrated on strain diversity and functions [8], but little was discovered regarding secondary metabolites and their activities, particularly for endophytic bacteria. More than 65% of the antibiotics used in clinical settings were produced by Actinobacteria, making them as the most productive bacterium [9]. Actinobacteria from various and distinctive environments are being studied by researchers as result of this substantial contribution to medication discovery.

Fenghuang Mountain (Yixing city, Jiangsu Province, China) is a treasure trove of diverse natural medicines [10]. Due to the peculiar ecological conditions, it is thought to be replete with endophytes and secondary metabolites thereof. Here, we discuss the diversity and metabolomics profiling of endophytic strains isolated from nine medicinal plants gathered in Fenghuang Mountain, China, as part of our effort to look for bioactive natural products from various medicinal plants [11,12] and associated endophytes [13,14]. By using 16S rRNA or ITS sequencing, phylogenetic analysis was carried out. Utilizing metabolite profiles from HPLC-UV-MS data and antibacterial assay results, strain prioritization was carried out. Twenty-three secondary metabolites (**1**–**23**), including a new sorbicillin analogue, were produced after a bioassay-guided isolation, and they were then identified by using spectroscopic data analysis. We tested each isolated compound for the presence of antibacterial or antifungal properties (Supplementary Scheme S1).

## 2. Results

### 2.1. Endophytic Strain Isolation and Identification

**Figure 1:**
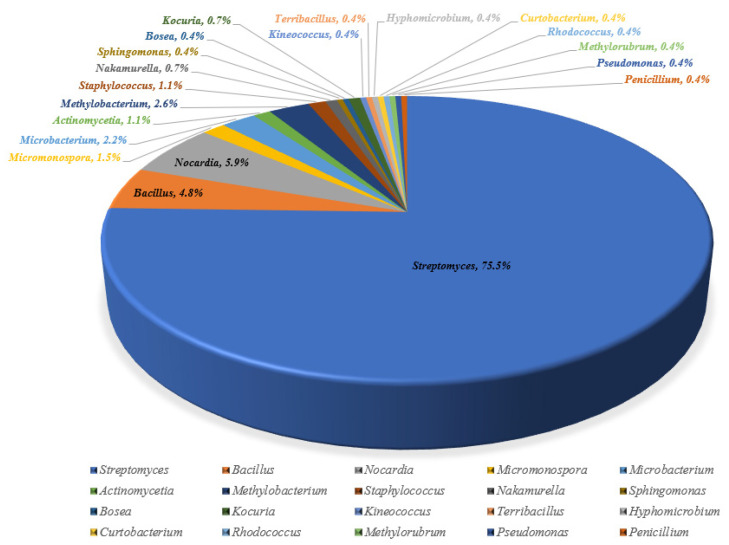
Diversity analysis of 269 endophytic strains from nine medicinal plants.

Nine different medicinal plants [*Achyranthes bidentata* Blume, *Ainsliaea fragrans* Champ., *Artemisia capillaris* Thunb., *Daucus carota* L., *Leonurus japonicus* Houtt., *Perilla frutescens* (L.) Britt., *Prunella vulgris* L., *Salvia miltiorrhiza* Bge. and *Stemona sessilifolia* (Miq.) Miq.] were gathered for endophyte cultivation. After plant tissues were surface sterilized, 269 endophytic microbes were gradually isolated and identified. With a few bacteria and one fungus (*Penicillium* sp. NX-S-6 from *Achyranthes bidentata*), the endophytes were primarily actinomycetes. Twenty genera, including *Streptomyces*, *Bacillus*, *Nocardia*, *Micromonospora*, and others, were represented by all of the strains, etc. Seventy-five percent of the total was *Streptomyces* (Figure 1 and Table 1, Supplementary Figures S1–S10 and Supplementary Tables S1–S9). Even after applying amphotericin B, *Penicillium* sp. NX-S-6 was the only endophytic fungus isolated. In the same geographical area, there were excellent chances that the same strain might be isolated from many plants or portions of the same plant.

### 2.2. Strain Prioritization

The majority of the isolated endophytic strains (1 mg/mL) showed inhibitory activity against the tested pathogenic bacteria (*E. coli* and *S. aureus*) in some way (see Supplementary Figures S14–S22). A few strains, such as strain No. 98 from *Achyranthes bidentata* (Supplementary Figure S14) and strain No. 28 from *Ainsliaea fragrans* (Supplementary Figure S15) significantly inhibited both *E. coli* and *S. aureus*. More strains exhibited inhibitory effects on *S. aureus* compared to *E. coli*. *Penicillium* sp. NX-S-6 (Supplementary Figure S23), *Streptomyces* sp. YHLB-L-2 (Supplementary Figure S24), and *Streptomyces* sp. ZLBB-S-6 (Supplementary Figure S25) were chosen for further secondary metabolites investigation after a thorough study of HPLC-MS data for strain crude extracts.

### 2.3. Scale-Up Fermentation and Isolation

**Figure 2:**
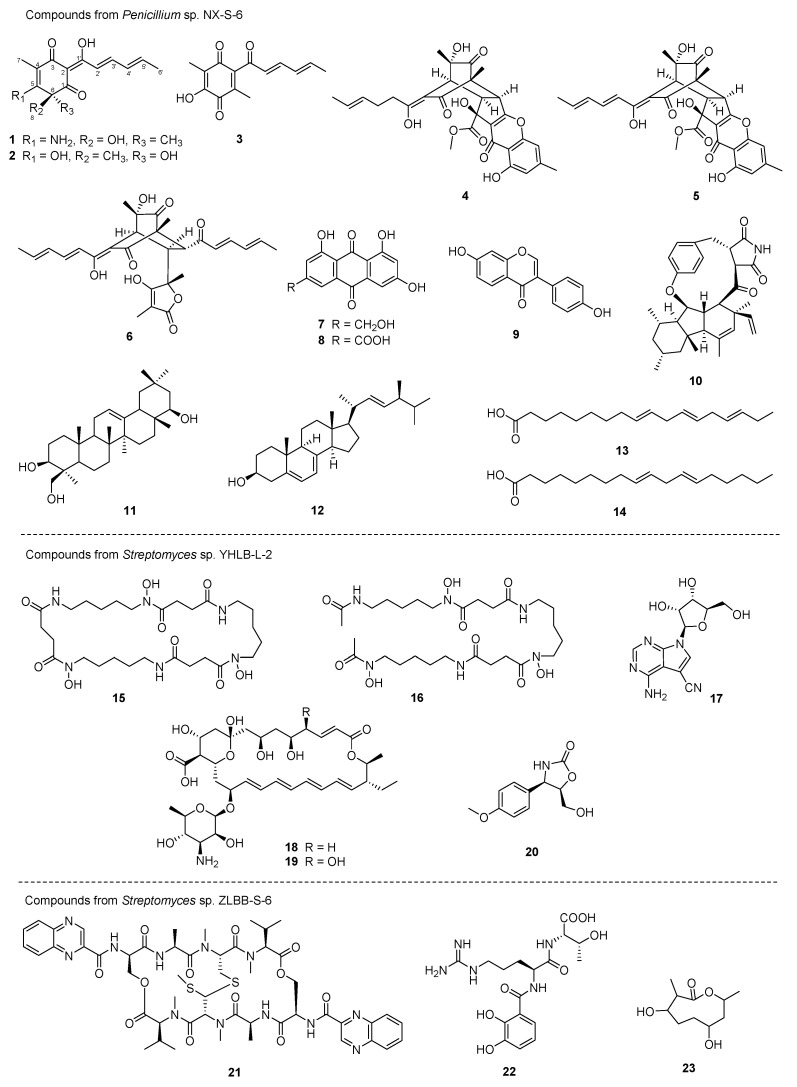
**1**–**23** isolated from three endophytes *Penicillium* sp. NX-S-6, *Streptomyces* sp. YHLB-L-2 and *Streptomyces* sp. ZLBB-S-6.

Scale-up fermentations of *Penicillium* sp. NX-S-6 (12 L of seed medium and 10 L of SDB medium), followed by extraction, fractionation and chromatographic purification (Supplementary Schemes S2 and S3), produced a new sorbicillin analogue, aminosorbicillinol (**1**), along with thirteen previously identified compounds: oxosorbicillinol (**2**) [15], sorrentanone (**3**) [16], bisorbicillchaetone A (**4**) [17], bisorbicillchaetone B (**5**) [17], bisorbibutenolide (**6**) [18], citreorosein (ω-hydroxyemodin) (**7**) [19,20,21,22], emodic acid (**8**) [23], daidzein (**9**) [24,25], GKK1032 B (**10**) [26], soyasapogenol B (**11**) [27,28], ergosterol (**12**) [29,30], α-linolenic acid (**13**) [31] and linoleic acid (**14**) [32] (Figure 2).

Similar scale-up fermentation (10 L) and subsequent extraction, fractionation and chromatographic purification (Supplementary Scheme S4) allowed the isolation of six natural products from *Streptomyces* sp. YHLB-L-2, including nocardamine (**15**) [33,34], desferrioxamine D1 (**16**) [33], toyocamycin (**17**) [35], tetramycin A (**18**) [36], tetramycin B (**19**) [37] and cytoxazone (**20**) [38]. Three compounds—quinomycin A (**21**) [39,40], benarthin (**22**) [41] and actinolactomycin (**23**) [42] (Figure 2)—were isolated in the same way (from *Streptomyces* sp. ZLBB-S-6 (Supplementary Scheme S5).

### 2.4. Structure Elucidation

**Figure 3:**
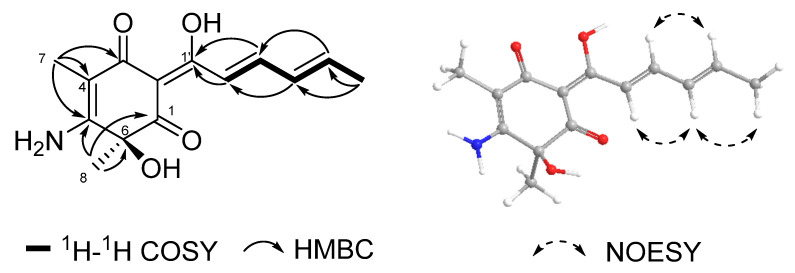
Key COSY, HMBC and NOESY correlations of **1**.

Compound **1** was obtained as a yellowish powder, and its molecular formula was established as C_14_H_17_NO_4_ by using HRESIMS data ([M + H]^+^, *m*/*z* 264.1231) with 7 degrees of unsaturation. Its ^1^H NMR and ^13^C NMR (Supplementary Figures S30 and S31) revealed three carbonyls/enols (*δ*_C_ 185.1, 187.9, and 196.5), three sp^2^ quaternary carbons (*δ*_C_ 97.3, 103.4, and 163.5), one oxygenated quaternary carbon (*δ*_C_ 75.0), four sp^2^ methines (*δ*_C_ 123.8, 131.2, 140.4, and 144.1) and three methyl (*δ*_C_ 7.6, 18.9 and 32.8). The NMR data of compound **1** and oxosorbicillinol (**2**) [15] were strikingly comparable (Table 2). The COSY coupling system of H-2′/H-3′/H-4′/H-5′/H_3_-6′ and two HMBC correlations of both H-2′ and H-3′ to an enol (C-1′, *δ*_C_ 185.1) indicated the presence of a single sorbyl chain. The HMBC correlations from H_3_-7 (*δ*_H_ 1.80) to C-3 (*δ*_C_ 187.9), C-4 (*δ*_C_ 97.3) and C-5 (*δ*_C_ 163.5) suggested that 7-CH_3_ was situated at C-4. According to HMBC correlations from H_3_-8 (*δ*_H_ 1.58) to C-1 (*δ*_C_ 196.5), C-5 (*δ*_C_ 163.5) and C-6 (*δ*_C_ 75.0) (Figure 3), the 8-CH_3_ was confirmed at the C-6 position. Due to the distinct chemical shifts, the only structural variation between **1** and oxosorbicillinol (**2**) was a C-5 substitution. Instead of a hydroxyl group (OH) at C-5 in **2**, an amide group (NH_2_) was present in **1**. The NOESY correlations of H-2′/H-4′/H_3_-6′ and H-3′/H-5′ indicated clearly that the side chain double bonds were both in *E* configuration. Using the time-dependent density-function theory (TD-DFT) calculations on the ^13^C NMR spectroscopic data of 6*S*-**1** and 6*R*-**1**, the absolute configuration of C-6 was ascertained (Supplementary Table S10). The computational ^13^C NMR data of 6*S*-**1**, when analyzed using a multiple linear regression analysis approach [11], were significantly closer to the experimental values (Supplementary Figure S35), with a higher correlation coefficient (R^2^) of 0.9976. As a result, compound **1** was designated as a new sorbicillin member and named as aminosorbicillinol.

Twenty-two known compounds (**2**–**23**) were also isolated and identified from scale-up fermentations from *Penicillium* sp. NX-S-6, *Streptomyces* sp. YHLB-L-2 and *Streptomyces* sp. ZLBB-S-6. The structures were determined by comparison with previously reported spectroscopic data (Supplementary Figures S36–S76).

*Aminosorbicillinol (**1**)*. yellowish powder; [α^25^_D_ +8.0 (c 0.1, CH_3_OH); IR (KBr) *ν*_max_ 3338, 3193, 2930, 1697, 1642, 1526, 1435 cm^−1^; ^1^H and ^13^C NMR data, see Table 2; (−)-ESI-MS: *m*/*z* 262.2 [M − H]^–^; (+)-ESI-MS: *m*/*z* 264.2 [M + H]^+^; (+)-HR-ESI-MS *m*/*z* 264.1231 [M + H]^+^ (calcd. for C_14_H_18_NO_4_, 264.1236).

### 2.5. Antimicrobial Results

The first test performed on each of the yield compounds was a standard antibacterial assay. The antibacterial activities of compounds **3**, **8**, **10**, **14**, **17** and **21** against *Staphylococcus aureus*, *Bacillus subtilis*, *Escherichia coli*, *Pseudomonas aeruginosa* and *Acinetobacter baumannii* varied in intensity (Table 3). The most active one of these, quinomycin A (**21**) [40], was even more effective than two positive controls, ampicillin and polymyxin, against four of the test bacteria. Impressively, quinomycin A had a MIC of less than 0.125 µg/mL against *B. subtilis*. In order to further assess the antifungal efficacy of quinomycin A, standard and isolated clinical strains were used. With MIC values of 16, 4, 16 and 16 µg/mL, respectively, this natural product demonstrated potent inhibition against *Aspergillus fumigatus, Cryptococcus neoformans, Aspergillus fumigatus* #176 and *Aspergillus fumigatus* #339 (Table 4).

## 3. Discussion and Conclusions

One of the biggest concerns with human health worldwide continues to be antibiotic resistance. To address this issue, a thorough search for new and better antibiotics is currently in progress. A total of 269 endophytic strains were isolated and identified from the nine medicinal plants that were collected in Fenghuang Mountain. Twenty-three secondary metabolites (**1**–**23**) were isolated by bioassay-guided purification from three of the most active endophytes. Along with **2**–**6**, compound **1** was designed as a new compound that belongs to the sorbicillin family. The unusual hybrid sorbicillinoids known as bisorbicillchaetones A and B (**4**, **5**) were recently identified as coming from the deep-sea derived fungus *Penicillium* sp. SCSIO06868 with anti-inflammatory activities [17]. The production of compounds **7**–**9** by an endophyte has been demonstrated to occur often in secondary metabolites from medicinal plants [24]. A unique peptide alkaloid–polyketide hybrid called GKK1032 B (**10**) was previously produced from *Penicillium* sp. GKK1032. It was confirmed further by the isolation of the triterpenoid molecule soyasapogenol B (**11**) from an endophytic fungus in *Achyranthes bidentata* that plant endophytes might interact with their hosts’ metabolic process and generate analogs [43]. A bioactive sterol compound known as ergosterol (**12**) is frequently present in plants [29], edible mushrooms [44] and microorganisms [45]. Tetramycins A and B (**18**, **19**) are polyene macrolides with remarkable antifungal activities against *Corynespora cassiicola* [46] and *Fusarium oxysporum* f. sp. *cucumerinum* [47]. Quinomycin A (**21**) was the most effective antimicrobial compound against both Gram-positive and negative bacteria, as well as the standard and clinical isolated fungal strains, out of the 23 isolated compounds. Even better than ampicillin and polymyxin, quinomycin A’s MIC against *B. subtilis* was less than 0.125 µg/mL. Quinomycin A, also known as echinomycin, has previously been recognized as a possible anticancer drug that has a great affinity for double-stranded DNA [48]. Numbers of studies have demonstrated the strong antitumor activity of quinomycin A [49,50], and it has been the subject of multiple Phase II clinical trials for various cancers, including prostate cancer [51], breast cancer [52] and renal cell carcinoma [53]. Quinomycins were also discovered to have strong antibacterial properties [54,55], which may be related to their high cytotoxicity. But this warrants more thorough investigation.

Endophytes are now a crucial component in the synthesis of new bioactive natural products. The majority of these microorganisms, which include fungi and bacteria, reside in the intercellular gaps of plant tissues, and some of them create bioactive compounds that could be relevant to host–endophyte connections. Eventually, it might be demonstrated that these bioactive secondary metabolites are useful in medicine [56]. Our research highlights that the medicinal plants in Fenghuang Mountain harbor endophytic microorganisms that have the capacity to produce a variety of natural products with antimicrobial activity. This suggests that endophytes are an extraordinary source for the creation of novel antibiotics.

## 4. Materials and Methods

### 4.1. General Experimental Procedures

Infrared radiation spectra were recorded with a Thermo Scientific Nicolet iS5 FT-IR spectrometer (Thermo, Waltham, MA, USA). Nuclear magnetic resonance spectra were measured with a Bruker Advance AV500 spectrometer (Bruker, Mannheim, Germany). LC-MS was performed with an Agilent 1290 system equipped with a 6120 Quadrupole MSD mass spectrometer (Agilent Technologies, Santa Clara, CA, USA). HRESIMS spectra were recorded with a AB Sciex TripleTOF 5600 mass spectrometer (AB Sciex, Redwood City, CA, USA). HPLC analyses were conducted on an Agilent 1260 system with a PDA detector (Agilent Technologies, USA) and a Waters 2695 system with a 2998 PDA detector (Waters, Milford, MA, USA). Preparative HPLC separation was conducted on a Waters 1525EF LC system (Waters). MCI Gel high-porous polymer (75–150 μm) was obtained from Mitsubishi Chemical Corporation (Mitsubishi, Sagamihara, Japan). Sephadex LH-20 resin (25–100 μm) was obtained from GE Healthcare Company (GE Healthcare, Danderyd, Sweden). Amberlite XAD16N resin was purchased from Taitan Company (Shanghai, China). Chemicals were purchased from Aldrich or Juyou Company (Nanjing, China).

### 4.2. Culture Media

Oatmeal agar: oatmeal agar 65.0 g/L.

ISP4 agar: ISP4 37.0 g/L and agar 15.0 g/L.

LNMS agar: soluble starch 0.1 g/L, yeast extract 0.1 g/L, CaCO_3_ 0.02 g/L, NaCl 0.2 g/L, MgSO_4_·7H_2_O 0.05 g/L, K_2_HPO_4_ 2.0 g/L, FeSO_4_·7H_2_O 0.01 g/L and agar 18.0 g/L.

KMB agar: glycerin 10.0 g/L, peptone 20.0 g/L, K_2_HPO_4_ 1.5 g/L, MgSO_4_·7H_2_O 0.05 g/L and agar 15.0 g/L, pH was 7.2.

Modified Gaoshi No.2 agar: glucose 1.0 g/L, peptone 0.5 g/L, tryptone 0.3 g/L, NaCl 0.5 g/L, multivitamins (niacin, riboflavin, vitamin B1, vitamin B6, p-aminobenzoic acid, calcium pantothenate, inositol, each 0.5 mg, biotin 0.25 mg) and agar 20.0 g/L, pH was 7.2.

M2 agar: malt extract 10.0 g/L, glucose 10.0 g/L, yeast extract 4.0 g/L and agar 15.0 g/L.

CMA agar: corn flour 40.0 g/L and agar, 15.0 g/L.

YG agar: glucose 5.0 g/L, yeast extract 3.0 g/L, tryptone 5.0 g/L and agar 15.0 g/L.

Seed medium: glucose 10.0 g/L, corn flour 40.0 g/L, gluten powder 5.0 g/L, bran 10.0 g/L, K_2_HPO_4_·3H_2_O 0.5 g/L, (NH_4_)_2_SO_4_ 1.0 g/L and CaCO_3_ 2.0 g/L.

SDB medium: peptone, 10.0 g/L and glucose, 40.0 g/L.

Medium A: glucose 10.0 g/L, soluble starch 20.0 g/L, yeast extract 5.0 g/L, peptone 5.0 g/L, MgSO_4_·7H_2_O 0.5 g/L, NaCl 4.0 g/L, K_2_PO_4_ 0.5 g/L and CaCO_3_ 2.0 g/L.

### 4.3. Endophytic Strain Isolation

From Fenghuang Mountain (Yixing City, China), fresh samples of *Achyranthes bidentata*, *Ainsliaea fragrans*, *Artemisia capillaris*, *Daucus carota*, *Leonurus japonicus*, *Perilla frutescens*, *Prunella vulgris*, *Salvia miltiorrhiza*, and *Stemona sessilifolia* were gathered. All the plants mentioned above were identified by co-author Dr. Yang Hu. The voucher specimens (No. 2020005–2020013) were deposited in our Jiangsu Key Laboratory for Functional Substances of Chinese Medicine. Plant parts (leaves, roots, stems and flowers) were first cleaned with water before being submerged in 75% ethanol for 5 min and 2% sodium hypochlorite for 5 min, respectively. Tissue samples were washed three times in sterile water following each treatment. The surface-sterilized samples were divided into small pieces aseptically and 0.2 g of each was suspended in sterile water (1.0 mL) and heated at for one minute at 75 °C to kill nonsporulating bacteria [57]. ISP4 agar, oatmeal agar, LNMS agar, KMS agar, and Modified Gaoshi No.2 agar plates, which contained amphotericin B (25 μg/mL) and nalidixic acid (25 μg/mL), were streaked with the supernatant (100 μL). Each sporulating colony was selected and purified on additional M2 agar plates for pure culture after about a month of incubation at 28 °C [58].

### 4.4. Phylogenetic Analysis

#### 4.4.1. 16S rRNA Sequence for Bacteria

The target 16S rRNA gene was amplified using universal primers (27F 5′-AGAGTTTGATCCTGGCTCAG-3′, 1492R 5′-GGTTACCTTGTTACGACTT-3′) after bacteria were seeded in 4 mL of TSB broth for three days at 28 °C [13]. The amplified fragments (1200–1500 bps) were sent for sequencing analysis by Sangong Company (Shanghai, China). In order to find the closest match sequence, the 16S rRNA gene sequences of the endophytes were compared with the GenBank database using BlastN (https://blast.ncbi.nlm.nih.gov/Blast.cgi, accessed on 20 February 2023). The accession numbers for each of the retrieved 16S rRNA sequences were deposited in the NCBI nucleotide database.

#### 4.4.2. ITS Sequence for Fungus

From a fresh strain agar plate, 100 mg of fruiting bodies or mycelia were taken and ground with liquid nitrogen. The Fungal Genomic DNA Rapid Extraction Kit (Shanghai Sangon Biotechnology Company, Shanghai, China) was used to extract the DNA. The DNA samples were dissolved in 100 µL of TE Buffer and kept at −20 °C for storage. Using the universal primers: ITS1 5′-TCCGTAGGTGAACCTGCGG-3′; ITS4 5′-TCCTCCGCT TATTGATATGC-3′, the partial ITS gene fragment was amplified [59]. The amplified DNA sequence was analyzed by Shanghai Sangon Biotechnology Company (Shanghai, China). The ITS gene sequence was compared with the GenBank database using BlastN (https://blast.ncbi.nlm.nih.gov/Blast.cgi) for searching the sequence that was the closest match. The ITS sequence was deposited in the NCBI nucleotide database with accession number OQ438028.

### 4.5. Strain Prioritization

#### 4.5.1. Small Scale Fermentation and Extraction

Using 250 mL baffled conical flasks, individual microbial colonies were cultivated for 7 days at 28 °C in seed medium (50 mL). Then, prior to harvesting, 1 g of XAD-16 resin was applied. The culture of each strain was centrifuged in a falcon tube for 15 min at 5000 rpm. The supernatant was discarded and 30 mL of distilled water was used to wash the mycelia and XAD resin component twice before vortexing and centrifuging. The crude extract was then obtained by centrifuging and evaporating the supernatant after adding 15 mL of methanol and extracting by sonication for 30 min.

#### 4.5.2. HPLC-MS Analysis

The following procedure was used to subject the crude extracts for HPLC-MS analysis: Using an Agilent Eclipse Plus C18 column (2.1 × 100 mm, 1.8 µm), mobile phases were acetonitrile (A) and H_2_O with 0.05% FA (B), and the gradient program was 5–100% A in 0–5 min, 100% B in 5–7 min, 100–5% B in 7–8 min, 5% B in 8–10 min. The flow rate was 0.3 mL/min. The spectrum of wavelengths was 200–600 nm. 120 eV for API-MS. Prioritization would begin with strains that produced an abundance of products with comparable UV absorbances (Supplementary Figures S23–S25).

#### 4.5.3. Preliminary Antimicrobial Activities Screening

*Staphylococcus aureus* (No. ATCC 29213) and *Escherichia coli* (No. ATCC 25922) were acquired from the China Center of Industrial Culture Collection. All of the examined microorganisms were incubated in LB broth media overnight at 37 °C. The cultures were quantified using a spectrophotometer and diluted to A = 0.02 (OD_600_) before being distributed to 96-well plates (100 µL/well). The crude extracts of endophytes (1.0 mg/mL) and positive control (ampicillin, 0.2 mg/mL) were subsequently added to the 96-well plates (100 µL/well, n = 3). The plates were examined for absorbance at 600 nm using a microplate reader to determine the inhibitory rate following a 16 h incubation at 37 °C.

Inhibition (%) = [(Model − Blank) − (Test − Blank)] × 100%/(Model − Blank)

### 4.6. Fermentation, Extraction and Isolation

#### 4.6.1. *Penicillium* sp. NX-S-6

Two different media were used to ferment the strain twice (Supplementary Schemes S2 and S3).

Firstly, portions of the fully grown strain were transferred from M2 agar plates into ten 250 mL Erlenmeyer flasks with 50 mL of seed medium. The seed cultures (each 1 mL) were moved from the three days of incubation at 28 °C (200 rpm) to the 50 mL seed media (for a total of 240 flasks). After 7 days of incubation at 28 °C with 200 rpm agitation, the fermentation broth was combined and filtered to afford mycelium and supernatant. The mycelium component was extracted using MeOH (3 × 2.0 L) under sonication. The supernatant portion was then mixed with XAD-16 resin (4% *w*/*v*) and stirred for six hours. Following filtration, the resin was three times (500 mL each) washed with water, and three times (500 mL each) extracted with methanol. HPLC results showed that the metabolite profiles of the mycelial and supernatant fractions were comparable; thus, they were combined and concentrated to afford crude extract (101.63 g). The crude extract underwent further ethyl acetate (EtOAc) extraction to afford EtOAc extract (29.56 g), which was then isolated using a silica gel chromatographic column (800 g, 10 × 60 cm) with gradient petroleum ether/EtOAc system to produce 13 fractions (A–M). Fraction C (1.13 g) was purified on a Sephadex LH-20 column eluted with DCM: MeOH = 1: 1 to obtain sub-fraction C1 (0.1 g), which was further purified by a preparative HPLC (80% MeCN) to obtain compounds **13** (4.1 mg) and **14** (28.4 mg). Fraction D (5.05 g) was crystallized to obtain compound **12** (182.1 mg). Fraction F (0.56 g) and fraction J (0.95 g) were combined and purified on a Sephadex LH-20 column with DCM: MeOH = 1: 1 to yield compounds **9** (15.7 mg) and **10** (14.1 mg). Fraction I (0.47 g) was purified through a Sephadex LH-20 column (DCM: MeOH = 1: 1) to obtain compound **11** (20.0 mg). Sub-fraction I1 (18.9 mg) was purified by using a preparative HPLC (40% MeCN) to acquire compound **1** (5.6 mg).

Secondly, *Penicillium* sp. NX-S-6 cultivated on CMA agar plates at 28 °C was sliced and transferred into ten Erlenmeyer baffled flasks with 50 mL of SDB medium. After incubation at 28 °C for three days, the seed cultures (each 1 mL) were transferred to 100 mL of SDB media (a total of 100 flasks). The fermentation broth was combined and centrifuged at 4 °C (8000 rpm, 20 min) following two weeks of incubation at 28 °C with 200 rpm agitation. The mycelium component was extracted using MeOH (3 × 2.0 L) with sonication, and 54.46 g of crude extract A was produced after the solvent was evaporated. Crude extract B (17.69 g) was produced after the supernatant part was extracted with EtOAc three times and evaporated. Crude extract A (54.46 g) was isolated by using a silica gel column (900 g, 10 × 60 cm) and a petroleum ether/EtOAc system to yield 14 fractions (A′–N′). Fraction K′ (0.24 g) was purified on a Sephadex LH-20 column using MeOH to obtain sub-fraction K′1 (0.12 g) and compound **7** (3.1 mg). Sub-fraction K′1 was then purified by using a preparative HPLC (40% aqueous MeCN) to acquire compounds **3** (2.4 mg), **4** (14.6 mg) and **5** (3.9 mg). Compound **6** (10.1 mg) was isolated form fraction M′ (0.11 g) by using preparative HPLC (45% MeCN). Fraction N′ (0.80 g) was purified on a Sephadex LH-20 column using MeOH to obtain sub-fraction N′1 (61 mg) and compound **8** (2.1 mg). Sub-fraction N′1 was then isolated by using a preparative HPLC (30% MeCN) to yield compound **2** (7.1 mg).

#### 4.6.2. *Streptomyces* sp. YHLB-L-2

*Streptomyces* sp. YHLB-L-2 was grown for 7 days at 28 °C on M2 agar plates, as shown in Supplementary Scheme S4. Cut portions of fully-grown strain-containing agar were used to inoculate ten 50 mL flasks of medium A. The seed cultures (each 1 mL) were transferred to 100 mL of medium A (total 100 flasks) after three days of incubation at 28 °C (200 rpm). The fermentation broth was combined after 7 days of incubation at 28 °C with 200 rpm agitation, and then filtered to afford the mycelium and supernatant. The mycelium component was extracted with MeOH (3 × 2.0 L) under sonication. The supernatant fraction was then mixed with XAD-16 resin (4% *w*/*v*) and agitated for six hours. Following filtration, the resin was rinsed with water three times (500 mL each), and three times extracted with methanol (500 mL each). HPLC results showed that the metabolite profiles of mycelial and supernatant fractions were comparable; thus, they were mixed and concentrated to produce crude extract (56.32 g). The crude extract was firstly subjected to an MCI column (500 g, 9 × 40 cm) using a gradient MeOH/H_2_O system to obtain 11 fractions (A–K). Fraction C (1.32 g) was purified with a Sephadex LH-20 column using 80% MeOH as eluent to obtain compound **17** (39.0 mg) and sub-fraction C1. Compound **16** (7.7 mg) was isolated from sub-fraction C1 (142.6 mg) by using a preparative HPLC (23% MeOH). Fraction E (3.02 g) and fraction F (1.93 g) were mixed and purified by using a Sephadex LH-20 column (80% MeOH) to acquire compounds **15** (58.5 mg) and **20** (16.5 mg). Fraction H and fraction J were identified to be pure compounds **19** (0.74 g) and **18** (0.19 g) respectively.

#### 4.6.3. *Streptomyces* sp. ZLBB-S-6

As shown in Supplementary Scheme S5, *Streptomyces* sp. ZLBB-S-6 was grown for 7 days at 28 °C on M2 agar plates. In total, ten flasks of 50 mL of seed medium were inoculated with cut pieces of agar containing a fully grown strain. The seed cultures (each 1 mL) were transferred to 100 mL seed medium (total 100 flasks) after three days of incubation at 28 °C (200 rpm). The fermentation broth was combined after 7 days of incubation at 28 °C with 200 rpm agitation and then filtered to afford the mycelium and supernatant. Under sonication, the mycelium component was extracted with MeOH (3 × 2.0 L). XAD-16 resin (4% *w/v*) was added to the supernatant portion, and the mixture was agitated for six hours. Following filtration, the resin was three times washed with water (500 mL each), and three times extracted with methanol (500 mL each). The HPLC results showed that the metabolite profiles of mycelial and supernatant fractions were comparable; thus, they were combined and concentrated to produce crude extract (71.08 g). The crude extract was firstly subjected to an MCI column (500 g, 9 × 40 cm) using a gradient MeOH/H_2_O system to obtain 9 fractions (A-I). Fraction C (1.61 g) was isolated by using a Sephadex LH-20 column (80% MeOH) to obtain sub-fraction C1 (99.0 mg), which was further purified by preparative HPLC using 5% MeOH to obtain compound **22** (6.6 mg). Compounds **21** (85.0 mg) and **23** (20.4 mg) were isolated from fraction H (3.52 g) by using a flash column silica-cs (50 g) eluted with a gradient of DCM/MeOH.

### 4.7. Computational Methods

The Chem3D 15.0 software with PM3 force field was used to establish the initial conformations of target molecules. Then the conformational searches were conducted via random searching in the Schrodinger Maestro 2019 using MMFFs force field with an energy cutoff of 10.0 kcal/mol. Duplicate conformations were identified and removed when the root-mean-square distances (RMSD) were less than 0.5 Å for any two geometry-optimized conformations. Subsequently, the remaining conformations were re-optimized at the B3LYP/6-311G(d,p) level in vacuo by using the Gaussian 09 program [60]. The ^13^C NMR shielding constants were calculated with the time-dependent density-function theory (TD-DFT) methodology at the B3LYP/6-311+G(d,p) level in CDCl_3_ with the IEFPCM solvation model using Gaussian 09. Frequency analysis was performed as well to confirm that the re-optimized conformers were at the energy minima. The spectra were combined after Boltzmann weighting according to their population contributions using SpecDis 1.64 software [61]. Utilizing multiple linear regression analysis method [62], the computational ^13^C NMR data of 6*S*-**1** was consistent with experimental data (Supplementary Table S10), with a high correlation coefficient (R^2^) of 0.9976.

### 4.8. Antimicrobial Assay

#### 4.8.1. Antibacterial Activity

From CICC (China Center of Industrial Culture Collection, China), standard strains of *Staphylococcus aureus* (ATCC 29213), *Bacillus subtilis* (A186), *Escherichia coli* (ATCC 25922), *Pseudomonas aeruginosa* (ATCC 27853) and *Acinetobacter baumannii* (ATCC 19606) were purchased. The bacteria were inoculated in LB Broth media overnight at 37 °C. The cultures were quantified with a spectrophotometer, diluted to A = 0.02 (OD_600_) and then dispensed to 96-well plates (100 µL/well). All isolated compounds **1**–**23**, positive controls ampicillin and polymyxin (1–128 µM by double dilution method) and DMSO (placebo control) were then added (n = 3). Plates were incubated for 16 h at 37 °C, and the inhibitory rate was calculated by measuring the absorbance at 600 nm. When compared to positive control groups, bacterial growth inhibition greater than 80% was used to determine the MICs.

#### 4.8.2. Antifungal Activity

Standard *Aspergillus fumigatus* 1160, *Candida albicans* (ATCC 210231), *Cryptococcus neoformans* H99 and the clinically isolated strains *Aspergillus fumigatus* 77, 176, 185, 331, 339 and 353 were used as test strains. On YG agar plates, fungi were grown at a temperature of 37 °C. After two days, spores were collected, and the RPMI-1640 culture was used to modify the suspension concentrations. By using a blood counting chamber, the *Aspergillus fumigatus* strain final inoculated concentrations were prepared as 0.4 × 10^4^ to 5.0 × 10^4^ CFU/mL. The final inoculated concentrations of *Candida albicans* and *Cryptococcus neoformans* strains were prepared with McFar Land (OD_530_) to be 500~2500 CFU/mL. By using the double dilution procedure, compound **21** (0.125–32 µM) was added to 96-well plates (n = 3) and maintained for 48 h at 37 °C. Itraconazole served as the positive control. The MICs were acquired by ocular inspection without mycelia developing.

## Figures and Tables

**Table 1:** Summary of the endophytes from nine medicinal plants.

Host Plants	Endophyte Numbers
*Achyranthes bidentata*	124
*Ainsliaea fragrans*	13
*Artemisia capillaris*	11
*Daucus carota*	13
*Leonurus japonicus*	21
*Perilla frutescens*	15
*Prunella vulgris*	28
*Salvia miltiorrhiza*	16
*Stemona sessilifolia*	28
Total	269

**Table 2:** ^1^H NMR and ^13^C NMR Data for compounds **1** and **2**.

No.	1 ^1^		2 ^2^	
	*δ*_C_, Type	*δ*_H_ (*J* in Hz)	*δ*_C_, Type	*δ*_H_ (*J* in Hz)
**1**	196.5, C		196.5, C	
**2**	103.4, C		104.0, C	
**3**	187.9, C		191.3, C	
**4**	97.3, C		105.0, C	
**5**	163.5, C		174.1, C	
**6**	75.0, C		75.1, C	
**7**	7.6, CH_3_	1.80, s	7.8, CH_3_	1.73, s
**8**	32.8, CH_3_	1.58, s	28.1, CH_3_	1.40, s
**1** **’**	185.1, C		184.1, C	
**2** **’**	123.8, CH	7.34, d (15.1)	123.8, CH	7.36, d (15.2)
**3** **’**	144.1, CH	7.48, dd, (15.1, 11.0)	144.7, CH	7.46, dd, (15.2, 9.6)
**4** **’**	131.2, CH	6.35, dd, (14.5, 11.0)	131.4, CH	6.41, dd, (15.6, 9.6)
**5** **’**	140.4, CH	6.21, dq, (14.6, 6.7)	142.1, CH	6.38, dq, (15.6, 5.4)
**6** **’**	18.9, CH_3_	1.89, d (6.7)	19.2, CH_3_	1.88, d (5.4)

(^1^ CDCl_3_, 500 MHz; ^2^ DMSO-*d*_6_, 125 MHz).

**Table 3:** Antibacterial MIC results of some active compounds (µg/mL).

Bacteria	3	8	10	14	17	21	AMP	PB
*S. aureus*	128	8	128	64	-	0.5	16	-
*B. subtilis*	32	64	4	32	-	<0.125	64	32
*E. coli*	-	-	-	-	-	16	-	4
*A. baumannii*	-	64	-	-	-	<1	-	1
*P. aeruginosa*	-	-	-	-	64	-	-	<64

“-” indicated the antibacterial activity was greater than 128 µg/mL.

**Table 4:** Antifungal activity of quinomycin A (**21**) (MIC, µg/mL).

Organism		21	Itraconazole ^1^
Standard strains	*Aspergillus fumigatus*	16	1
	*Candida albicans*	>32	0.25
	*Cryptococcus neoformans*	4	<0.125
Clinical isolates (*Aspergillus fumigatus*)	176	16	<0.125
	339	16	<0.125
	353	>32	4
	331	>32	8
	77	>32	16
	185	>32	1

^1^ Positive control.

## Data Availability

The authors confirm that the data supporting the findings of this study are available within the article and its Supplementary Materials.

## Supplementary Materials

The following supporting information can be downloaded at: https://www.mdpi.com/article/10.3390/molecules28176301/s1.

## Sample Availability

Compound samples **1**–**23** are available from the authors.
