# Supplementary material for: Endophytic Microbes from Medicinal Plants in Fenghuang Mountain as a Source of Antibiotics
Source: Molecules. 2023 Aug 28;28(17):6301. doi: 10.3390/molecules28176301 (PMC10488465; doi:10.3390/molecules28176301)
Supplement: Supplementary file 1 [file molecules-28-06301-s001.zip › molecules-2504027-supplementary.pdf]

Supplementary Material

Endophytic Microbes from Medicinal Plants in Fenghuang Mountain as a Source of Antibiotics

Aiping Yang, Yu Hong, Fengjuan Zhou, Ling Zhang, Youjuan Zhu, Chang Wang, Yang Hu, Li Yu, Lihong Chen* and Xiachang Wang*

*** Correspondence:** Xiachang Wang: xiachangwang@njucm.edu.cn;
Lihong Chen: clh_helen@njucm.edu.cn

1 Supplementary Figures and Tables

1.1 Supplementary Figures

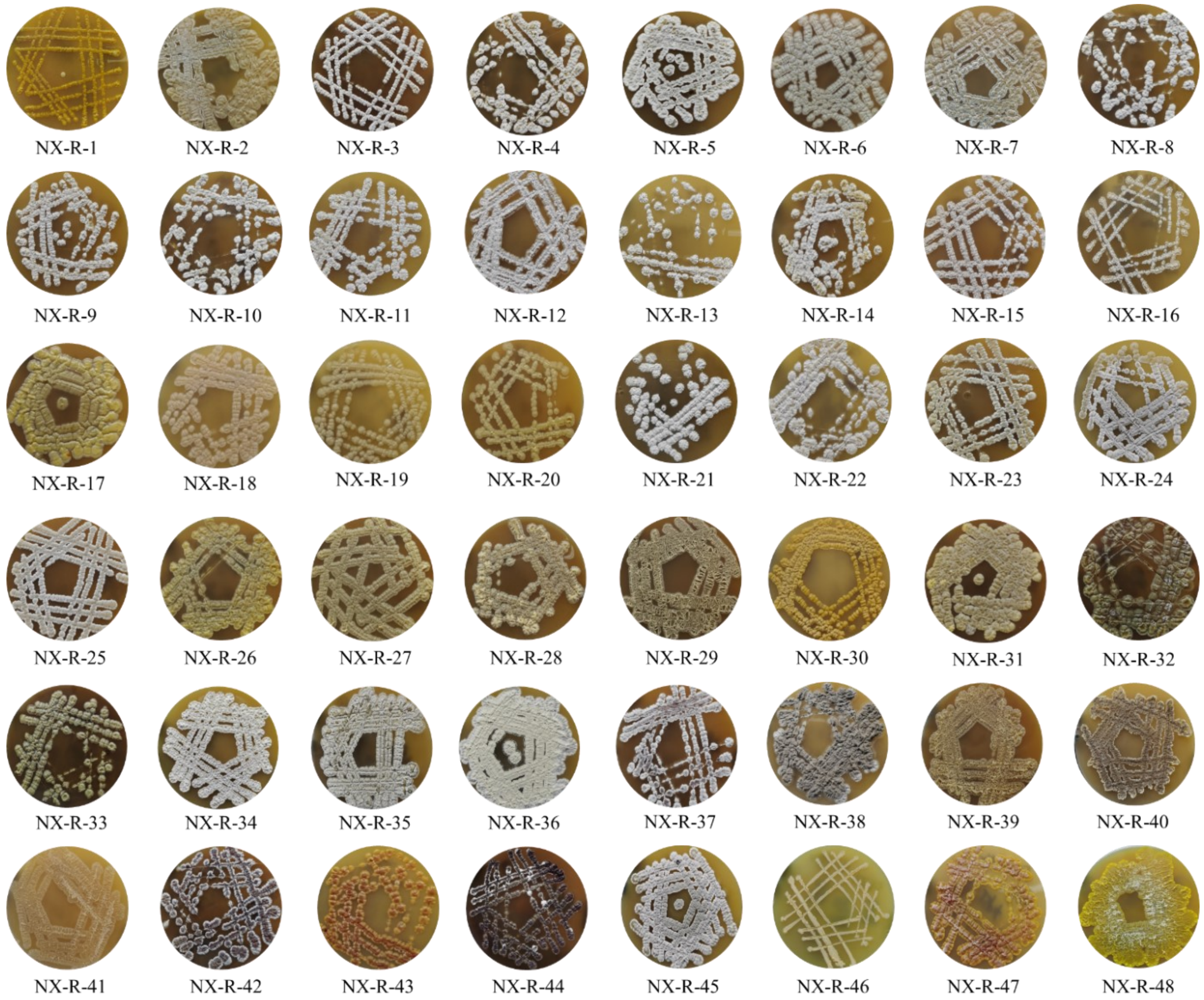

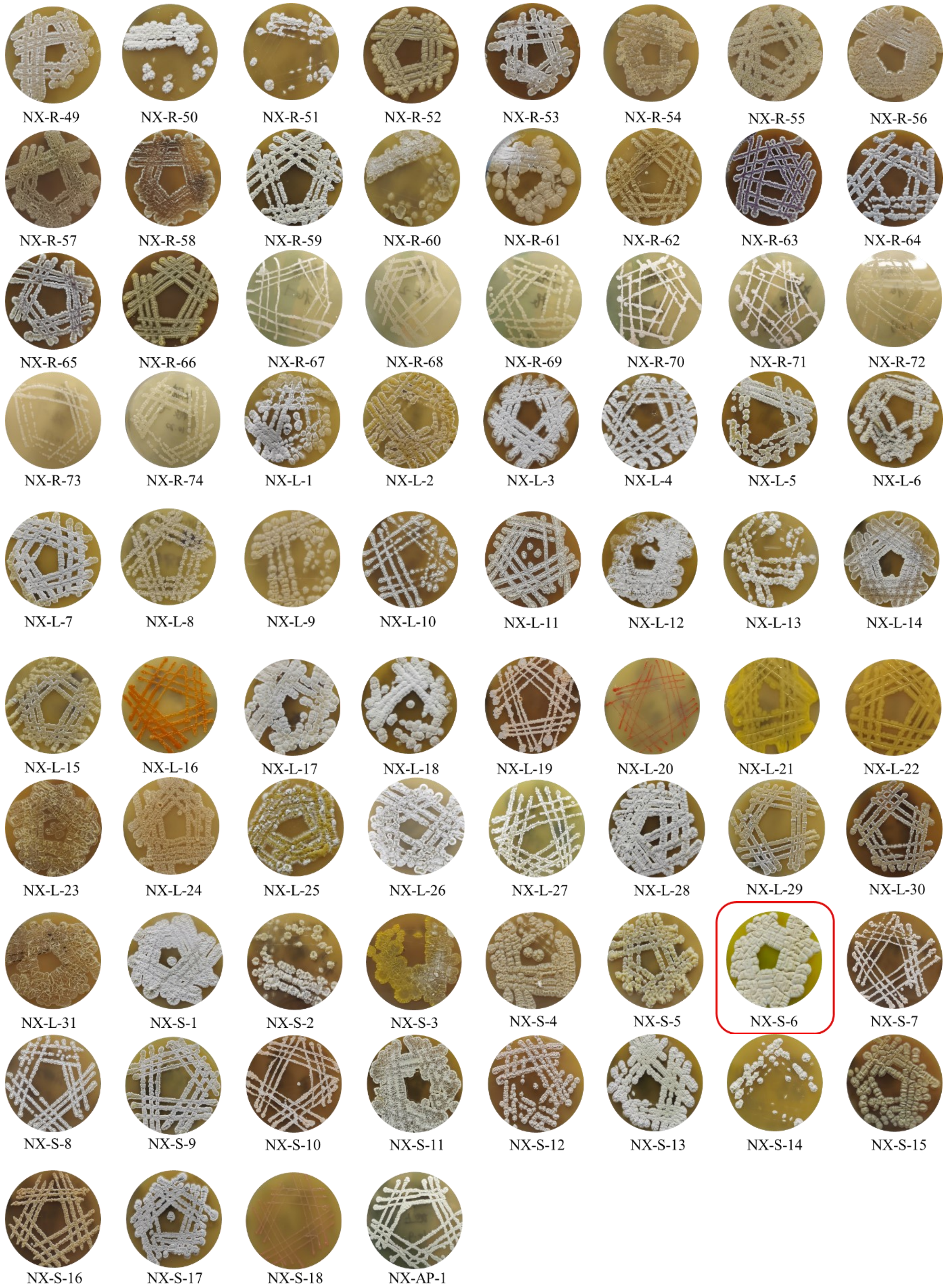

Supplementary Figure S1. Morphological features of 124 endophytic strains from *Achyranthes bidentata* (Red box: selected *Penicillium sp.* NX-S-6 for further study).

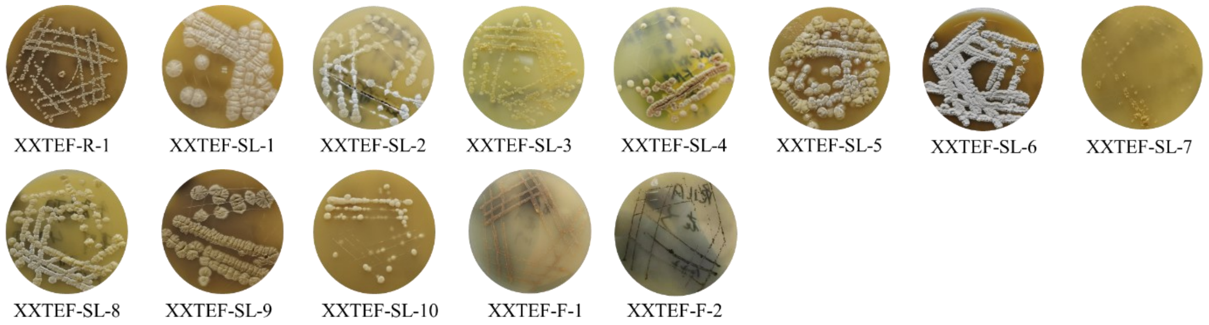

Supplementary Figure S2. Morphological features of 13 endophytic strains from *Ainsliaea fragrans*.

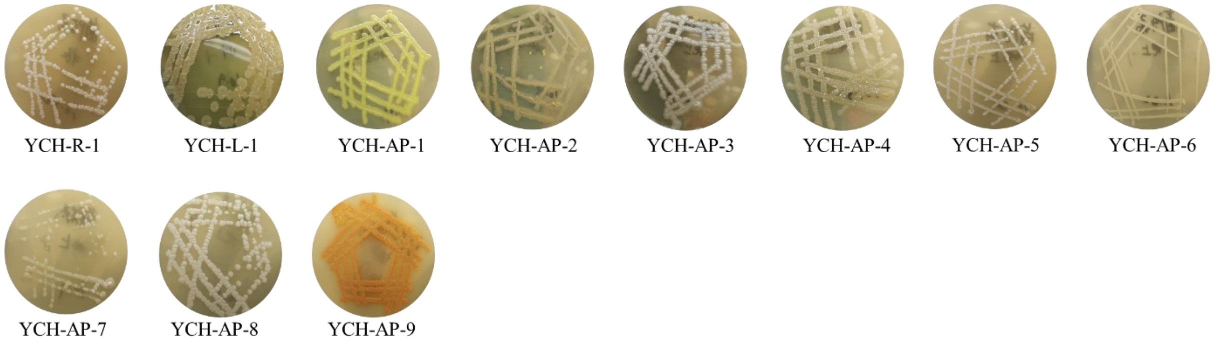

Supplementary Figure S3. Morphological features of 11 endophytic strains from *Artemisia capillaris*.

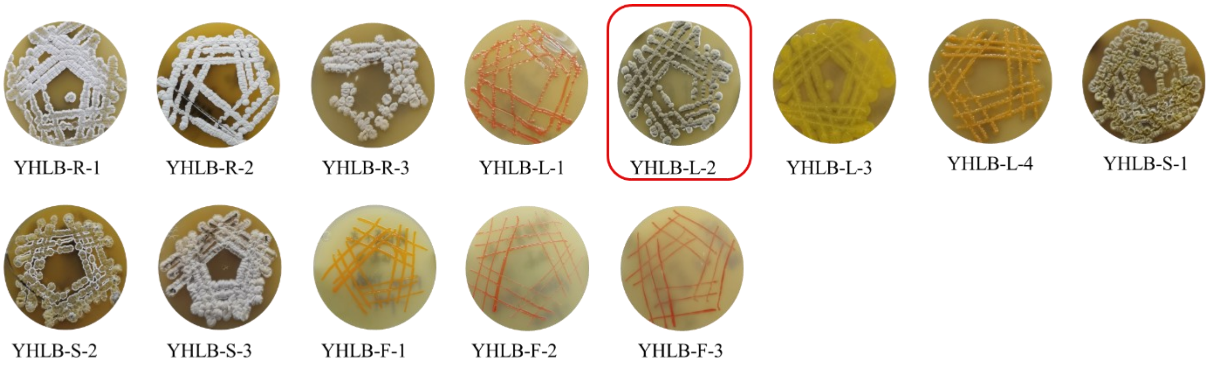

Supplementary Figure S4. Morphological features of 13 endophytic strains from *Daucus carota* (Red box: selected *Streptomyces sp.* YHLB-L-2 for further study).

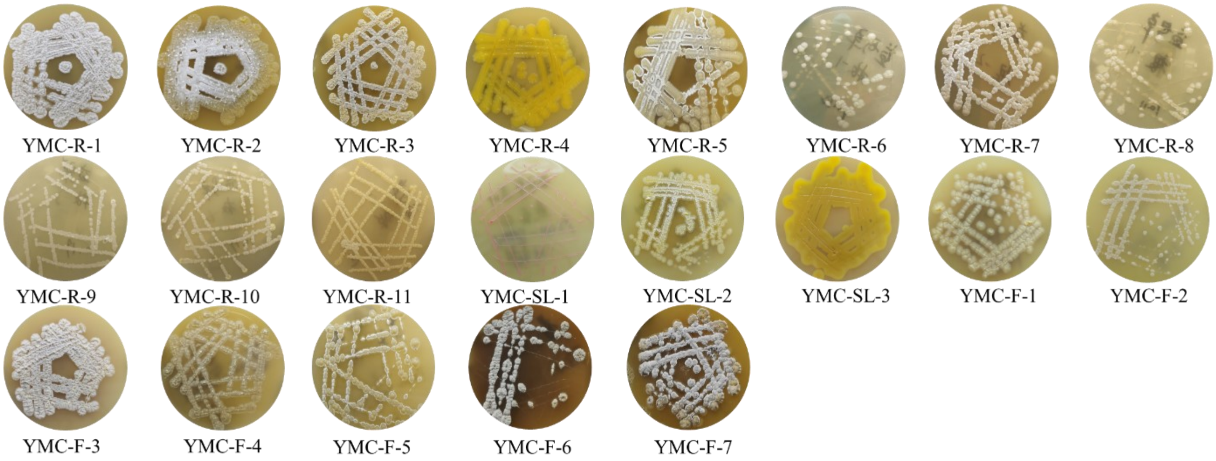

Supplementary Figure S5. Morphological features of 21 endophytic strains from *Leonurus japonicus*.

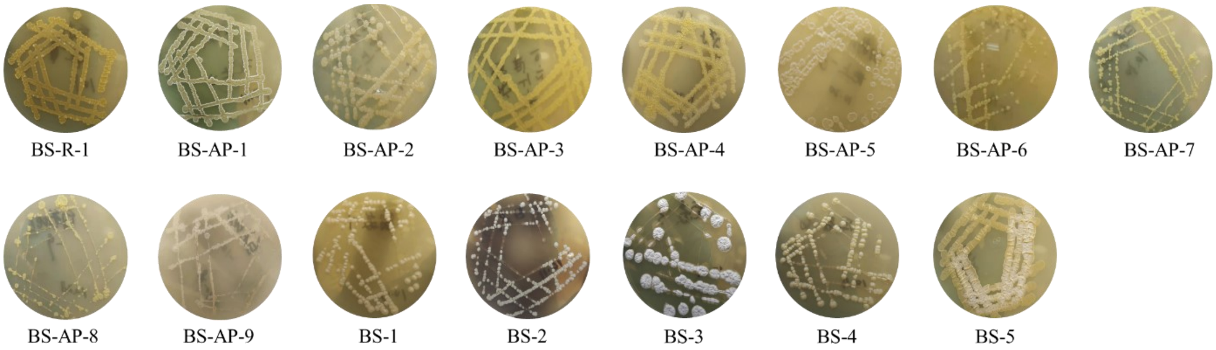

Supplementary Figure S6. Morphological features of 15 endophytic strains from *Perilla frutescens*.

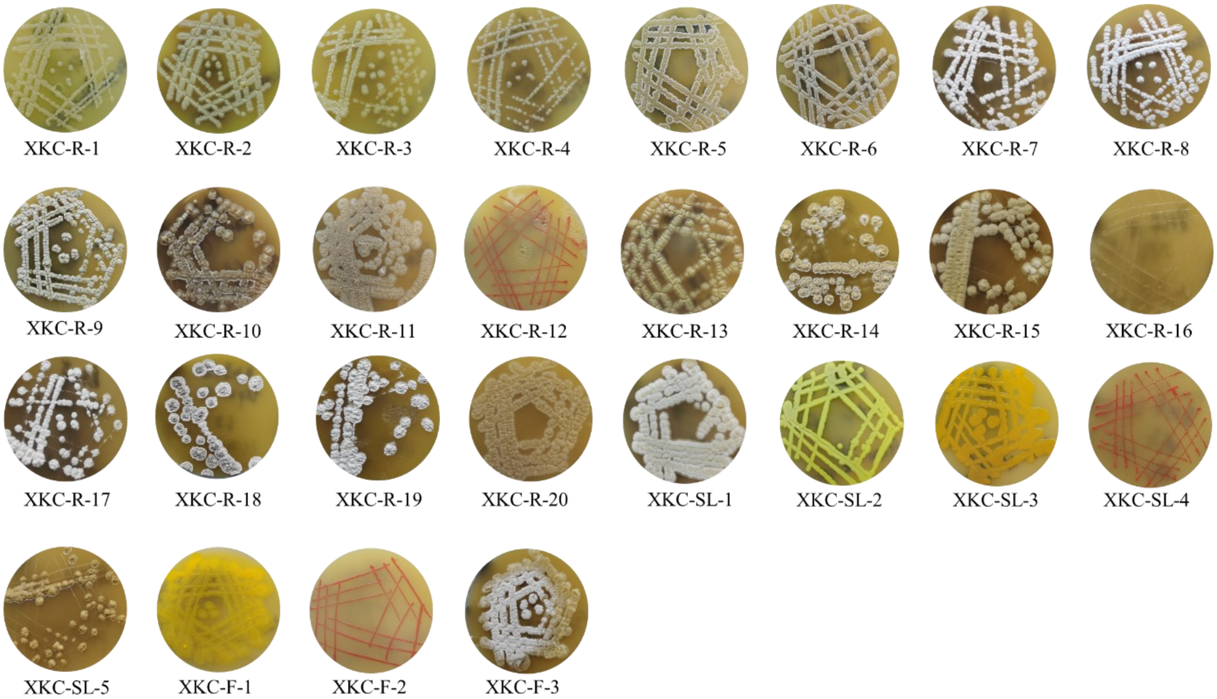

Supplementary Figure S7. Morphological features of 28 endophytic strains from *Prunella vulgris*.

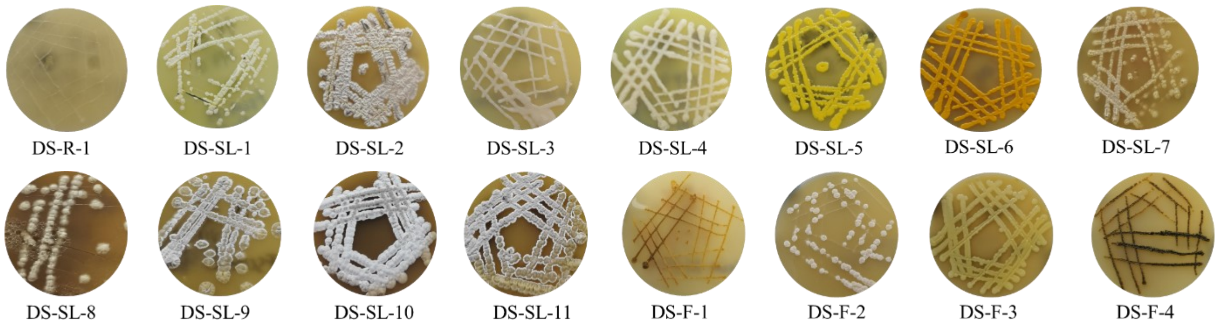

Supplementary Figure S8. Morphological features of 16 endophytic strains from *Salvia miltiorrhiza*.

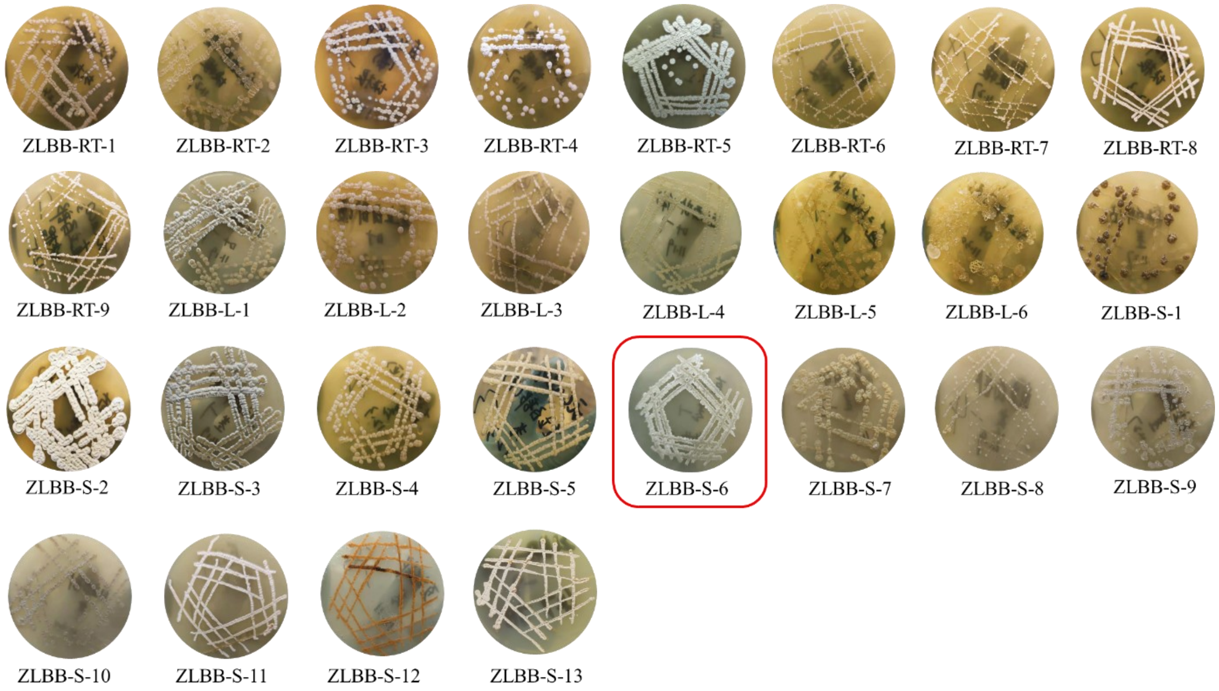

Supplementary Figure S9. Morphological features of 28 endophytic strains from *Stemona sessilifolia* (Red box: selected *Streptomyces sp.* ZLBB-S-6 for further study).

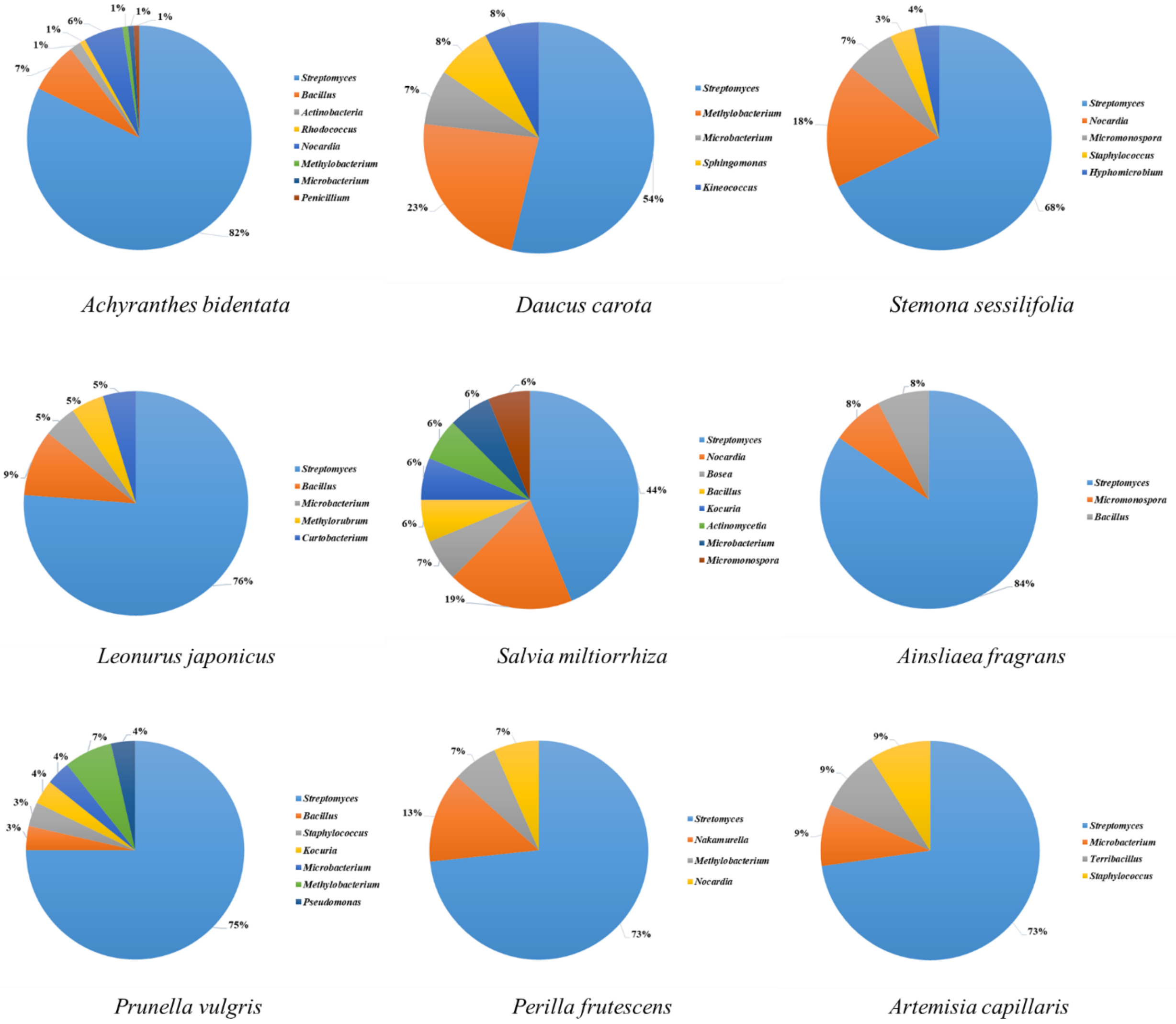

Supplementary Figure S10. Diversity analyses of endophytes from nine medicinal plants.

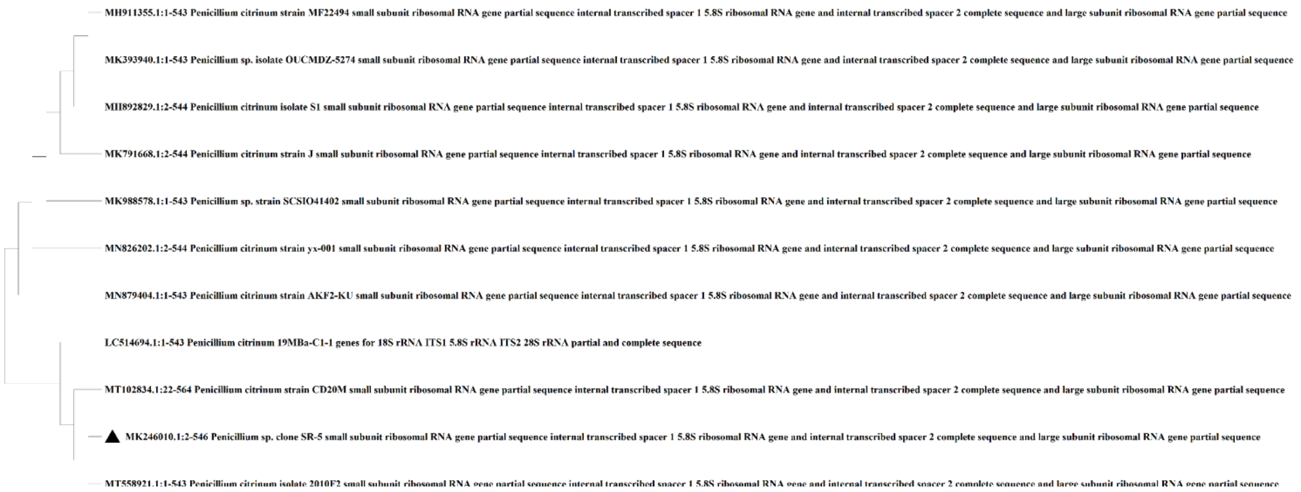

Supplementary Figure S11. Evolutionary relationship analysis of *Penicillium sp.* NX-S-6 (▲).

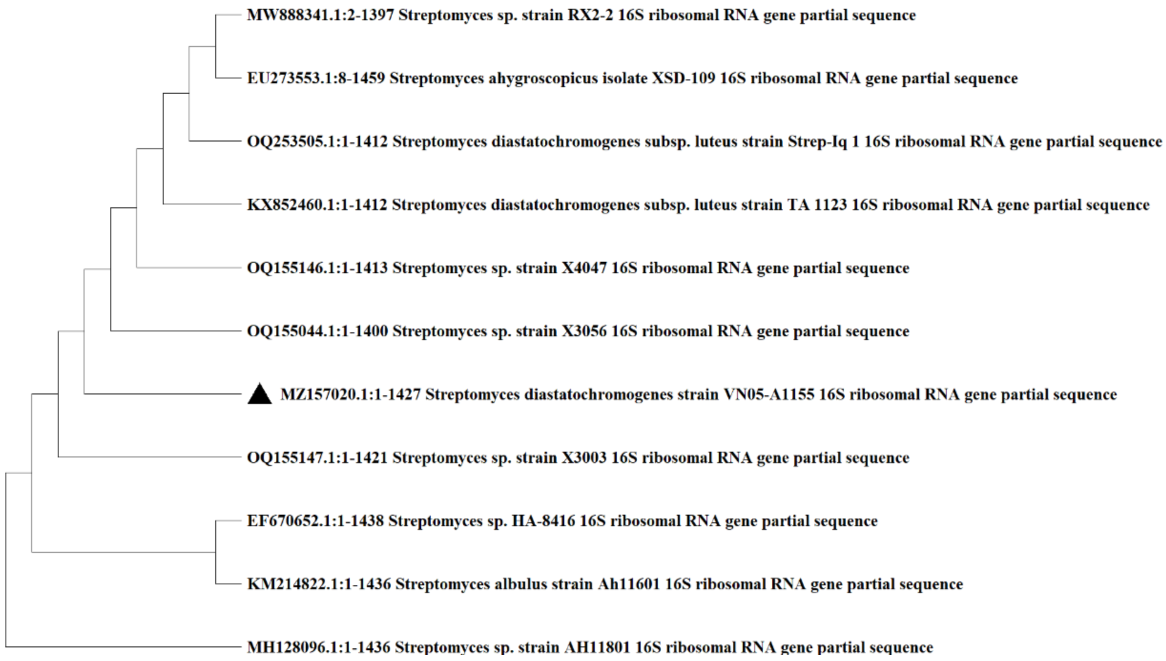

Supplementary Figure S12. Evolutionary relationship analysis of *Streptomyces sp.* YHLB-L-2 (▲).

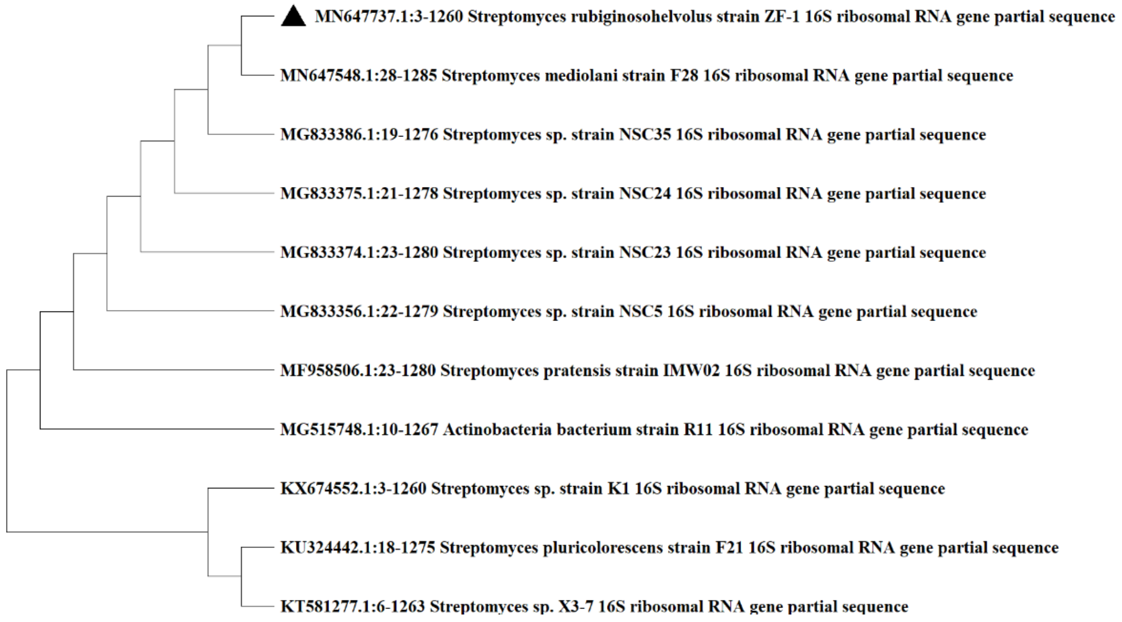

Supplementary Figure S13. Evolutionary relationship analysis of *Streptomyces sp.* ZLBB-S-6 (▲).

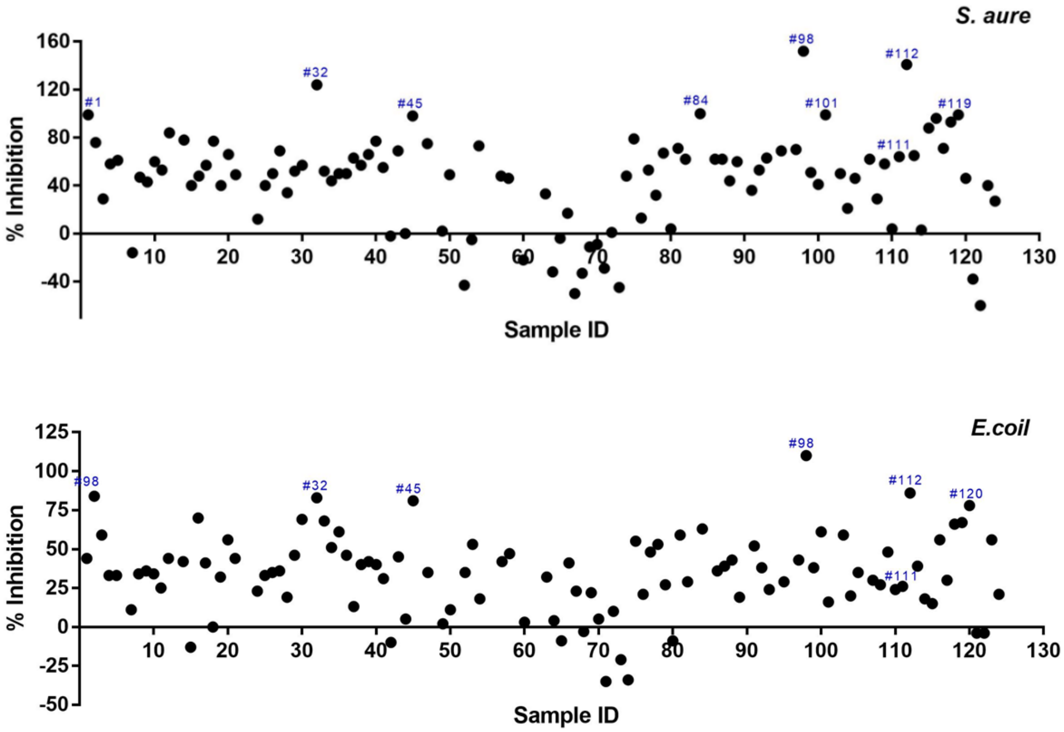

Supplementary Figure S14. Scatterplots of 124 strain extracts (1.0 mg/mL) from *Achyranthes bidentata* screened in *S. aureus* and *E. coli* inhibitory assay.

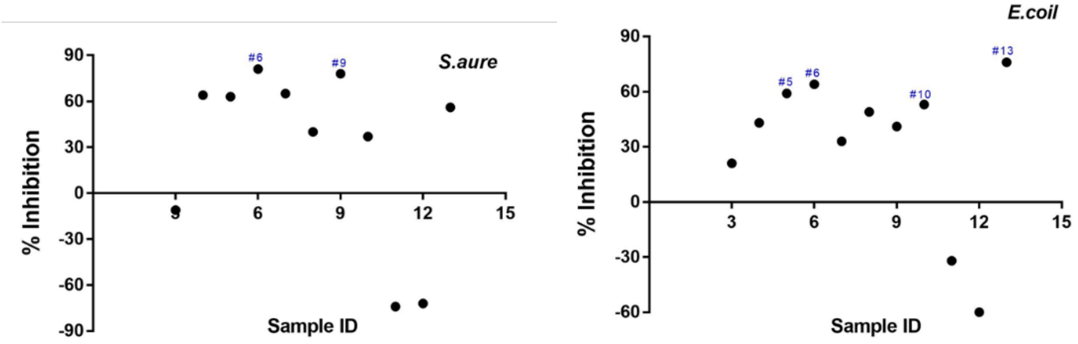

Supplementary Figure S15. Scatterplots of 13 strain extracts (1.0 mg/mL) from *Ainsliaea fragrans* screened in *S. aureus* and *E. coli* inhibitory assay.

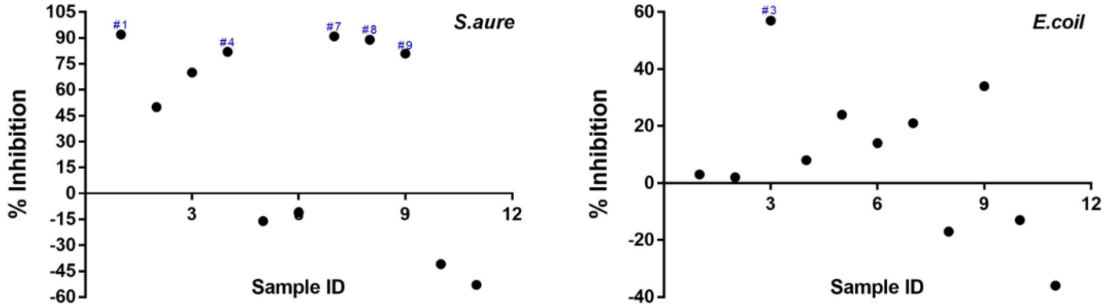

Supplementary Figure S16. Scatterplots of 11 strain extracts (1.0 mg/mL) from *Artemisia capillaris* screened in *S. aureus* and *E. coli* inhibitory assay.

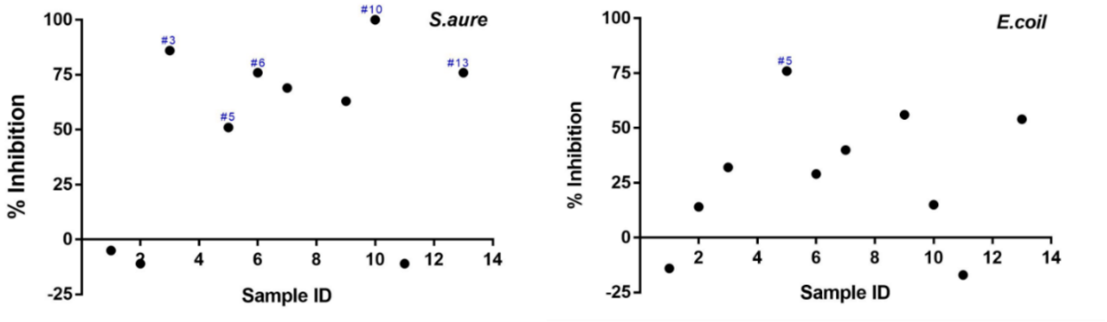

Supplementary Figure S17. Scatterplots of 13 strain extracts (1.0 mg/mL) from *Daucus carota* screened in *S. aureus* and *E. coli* inhibitory assay.

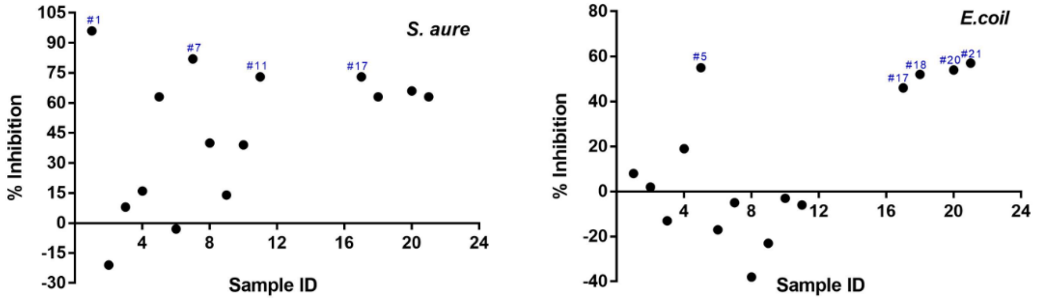

Supplementary Figure S18. Scatterplots of 21 strain extracts (1.0 mg/mL) from *Leonurus japonicus* screened in *S. aureus* and *E. coli* inhibitory analyses.

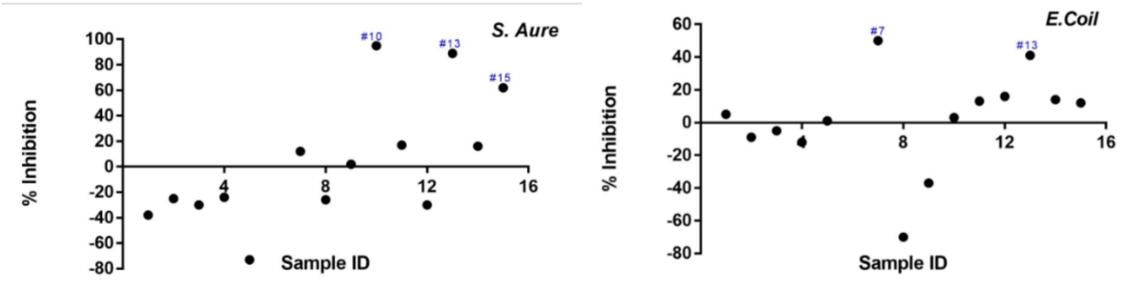

Supplementary Figure S19. Scatterplots of 15 strain extracts (1.0 mg/mL) from *Perilla frutescens* screened in *S. aureus* and *E. coli* inhibitory assay.

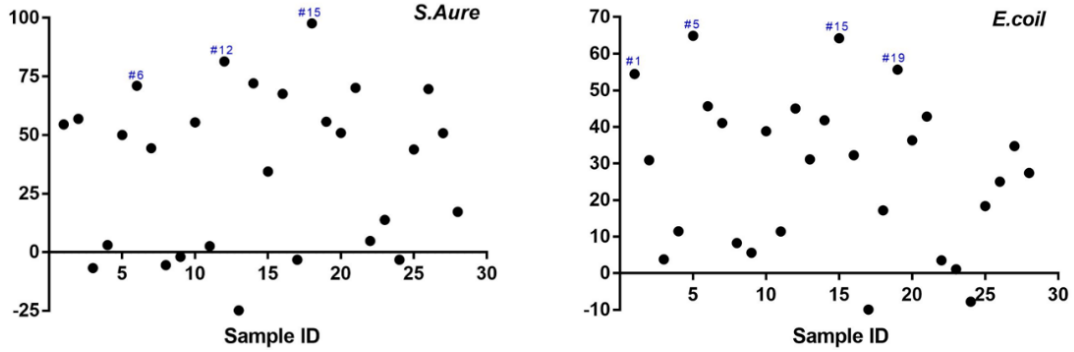

Supplementary Figure S20. Scatterplots of 28 strain extracts (1.0 mg/mL) from *Prunella vulgris* screened in *S. aureus* and *E. coli* inhibitory assay.

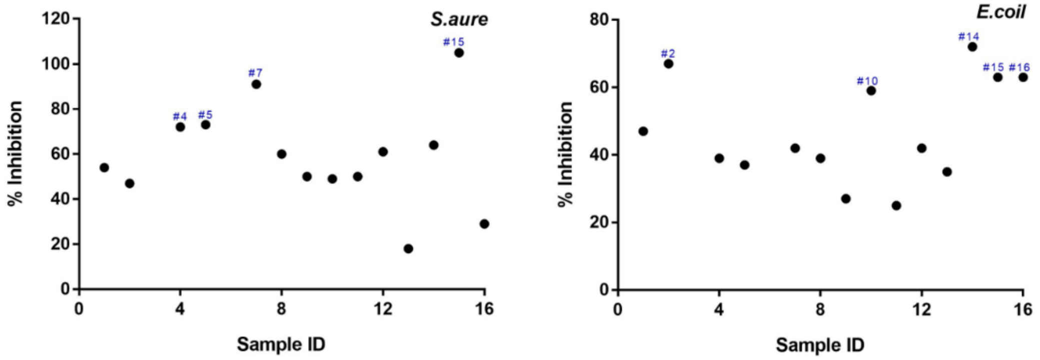

Supplementary Figure S21. Scatterplots of 16 strain extracts (1.0 mg/mL) from *Salvia miltiorrhiza* screened in *S. aureus* and *E. coli* inhibitory assay.

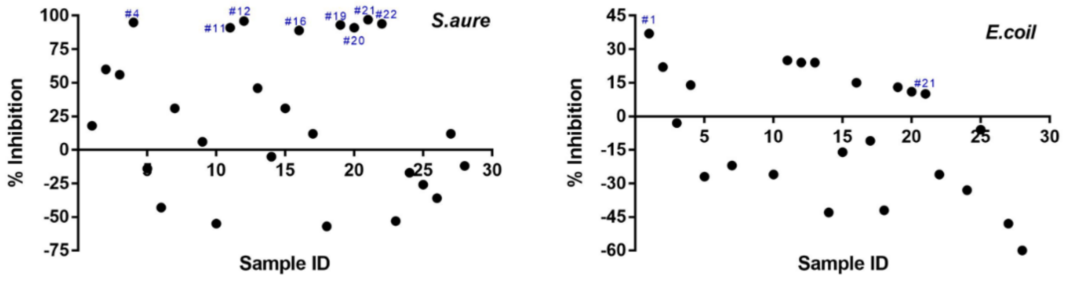

Supplementary Figure S22. Scatterplots of 28 strain extracts (1.0 mg/mL) from *Stemona sessilifolia* screened in *S. aureus* and *E. coli* inhibitory assay.

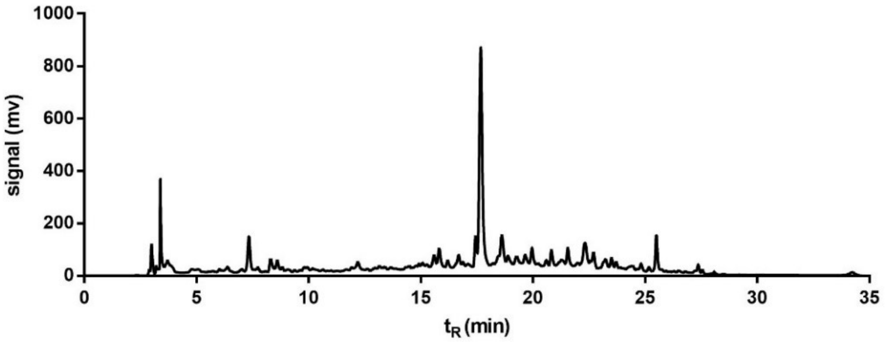

Supplementary Figure S23. HPLC spectrum of *Penicillium sp.* NX-S-6

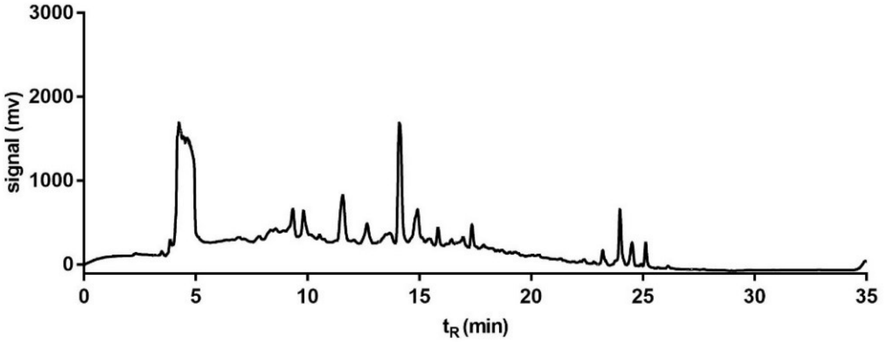

Supplementary Figure S24. HPLC spectrum of *Streptomyces* sp. YHLB-L-2

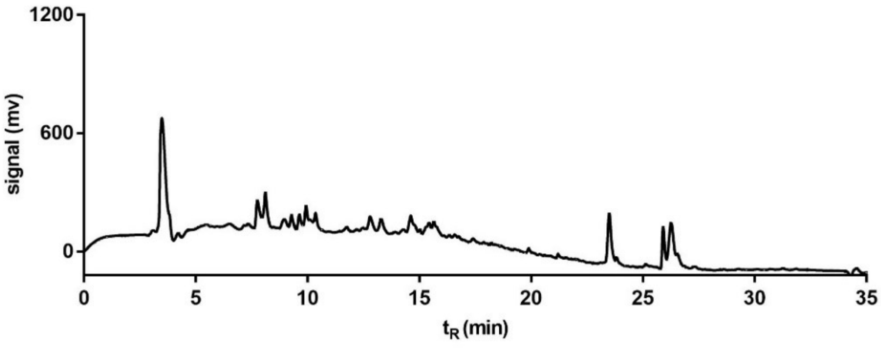

Supplementary Figure S25. HPLC spectrum of *Streptomyces* sp. ZLBB-S-6

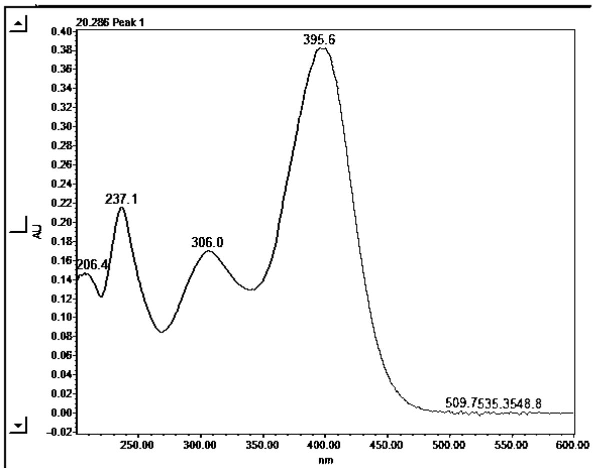

Supplementary Figure S26. UV spectrum of compound **1**.

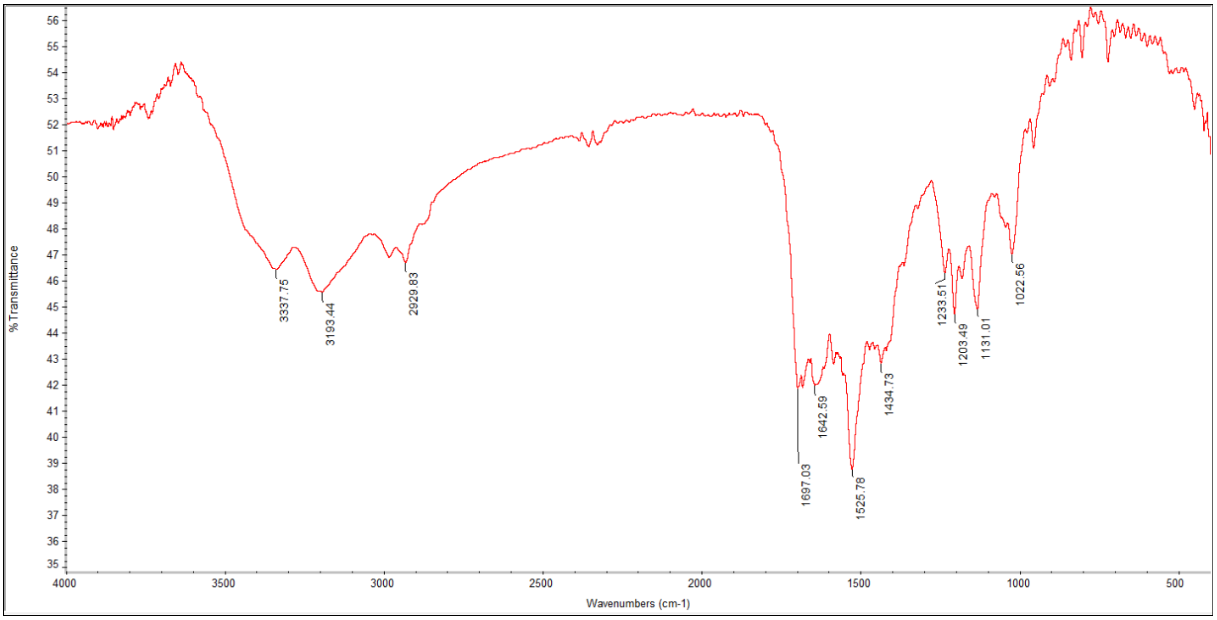

Supplementary Figure S27. IR spectrum of compound **1**.

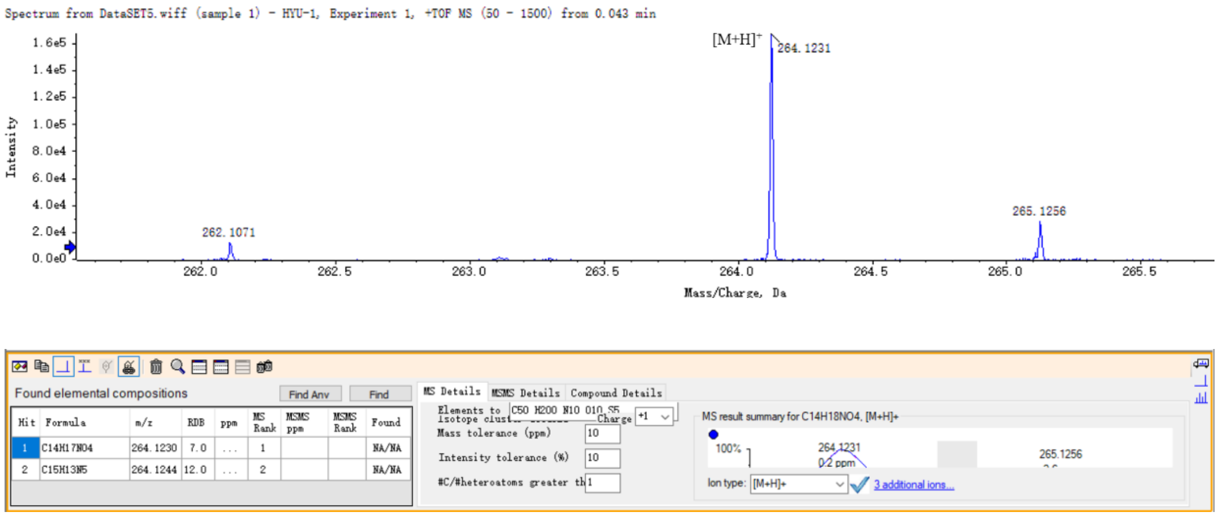

Supplementary Figure S28. (+)-HRESI-MS spectrum of compound **1**.

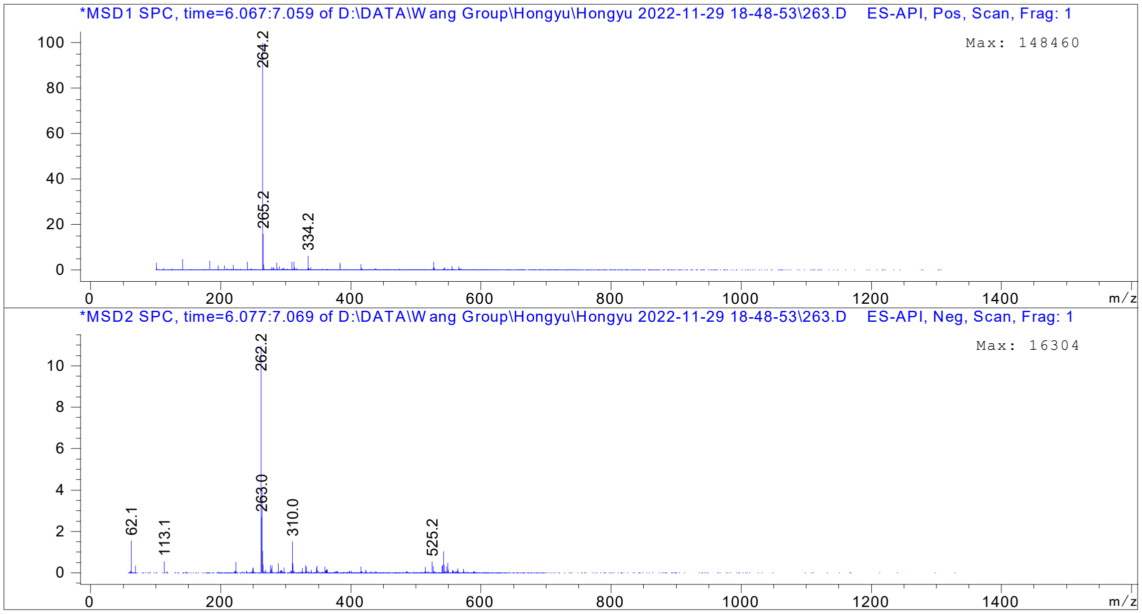

Supplementary Figure S29. (+) and (–)-ESI-MS of compound **1**.

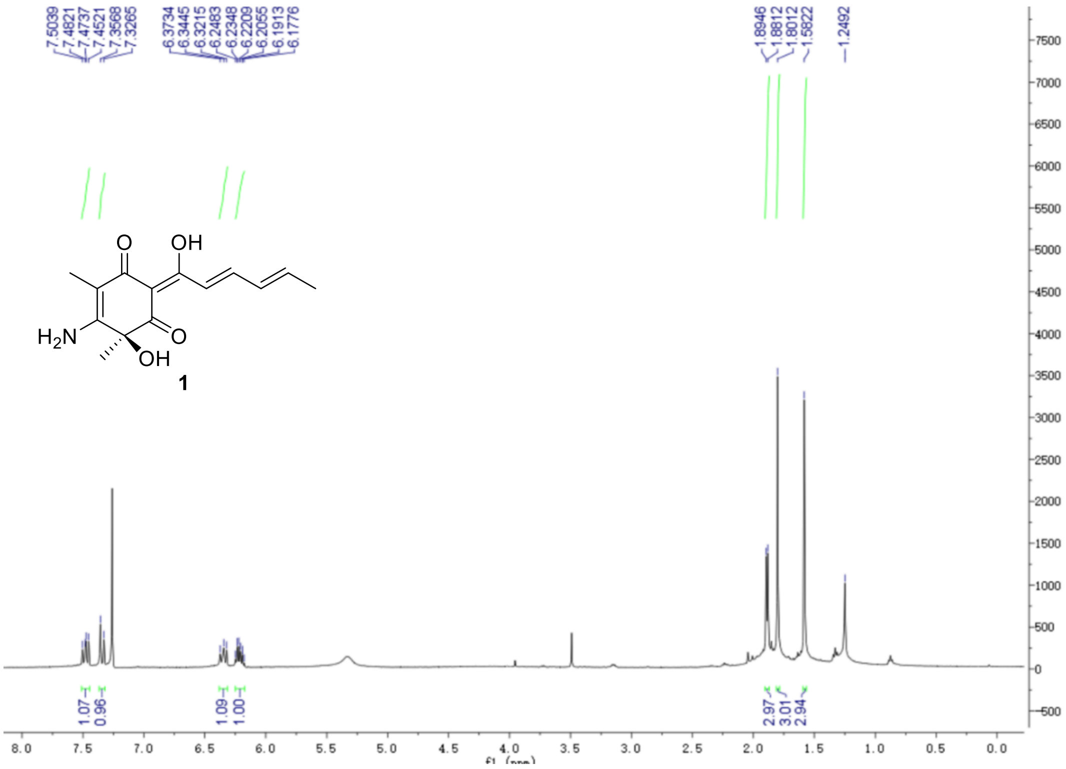

Supplementary Figure S30. ^{1}H NMR (500 MHz, $CDCl_3$) spectrum of compound **1**.

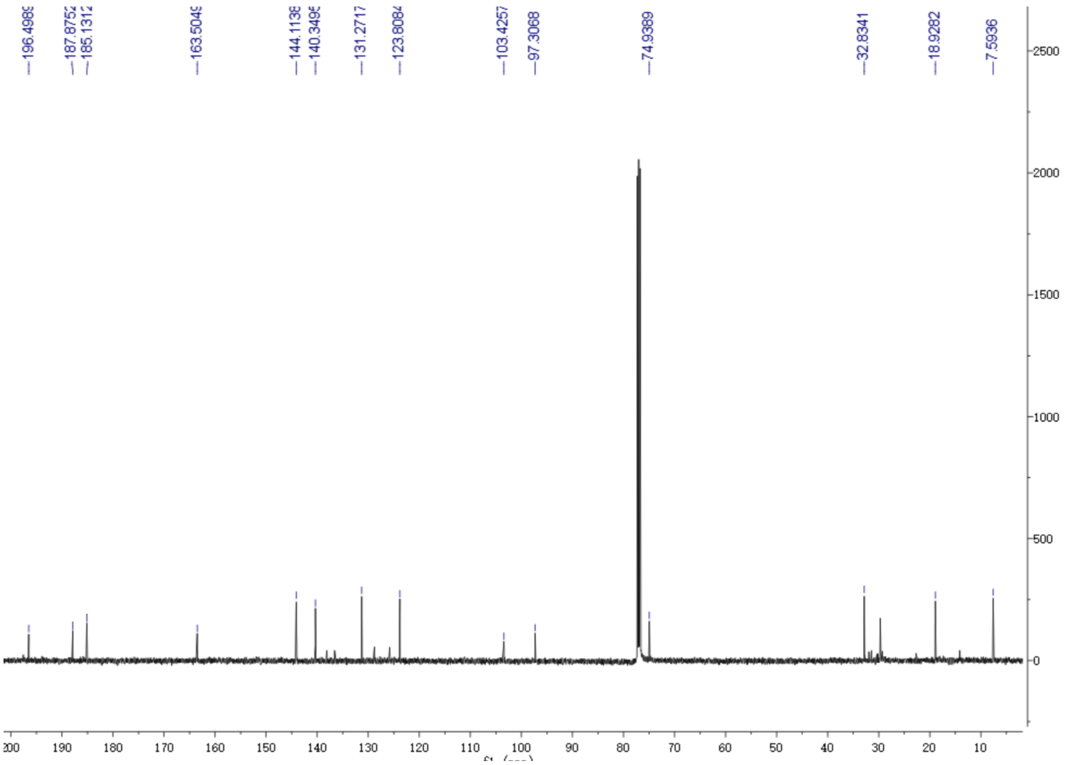

Supplementary Figure S31. ^{13}C NMR (125 MHz, $CDCl_3$) spectrum of compound **1**.

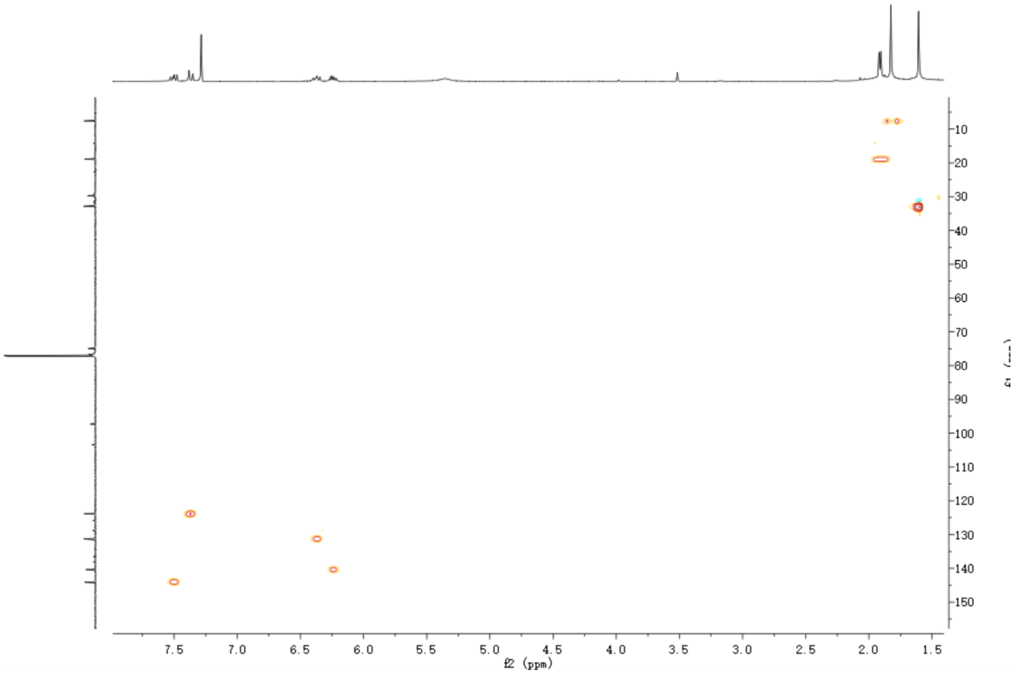

Supplementary Figure S32. HSQC (500 MHz, $CDCl_3$) spectrum of compound **1**.

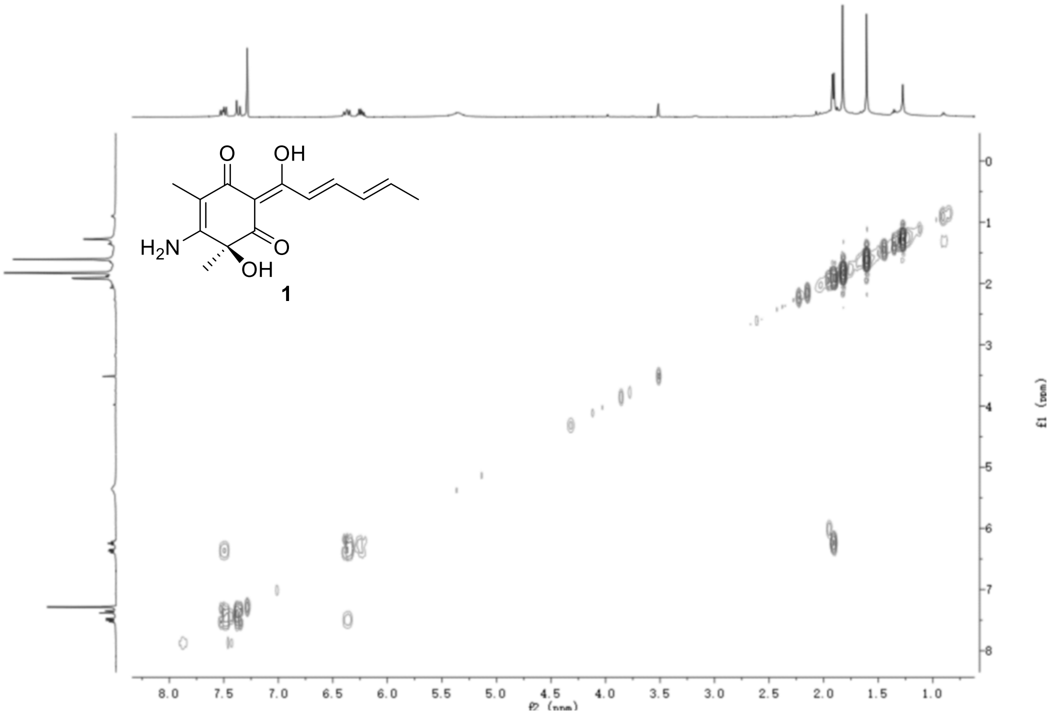

Supplementary Figure S33. ^1H-^1H COSY (500 MHz, $CDCl_3$) spectrum of compound **1**.

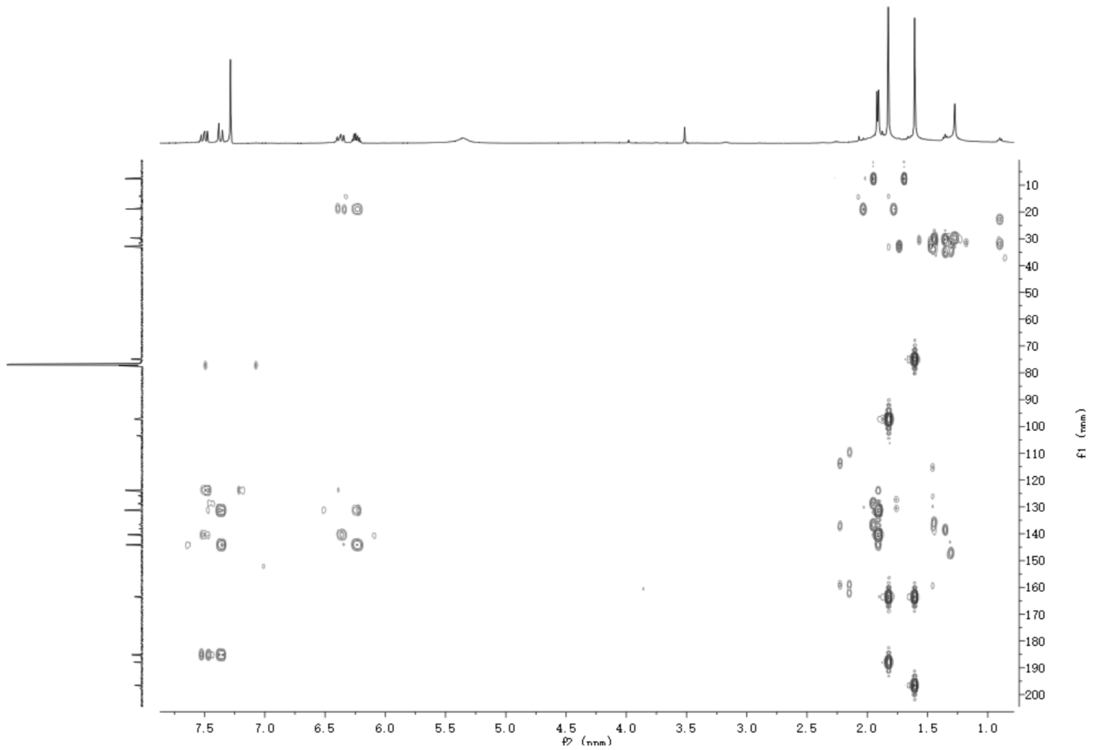

Supplementary Figure S34. HMBC (500 MHz, $CDCl_3$) spectrum of compound **1**.

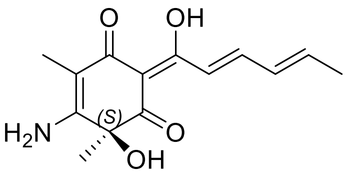

6*S*-**1**

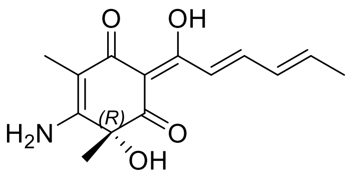

6*R*-**1**

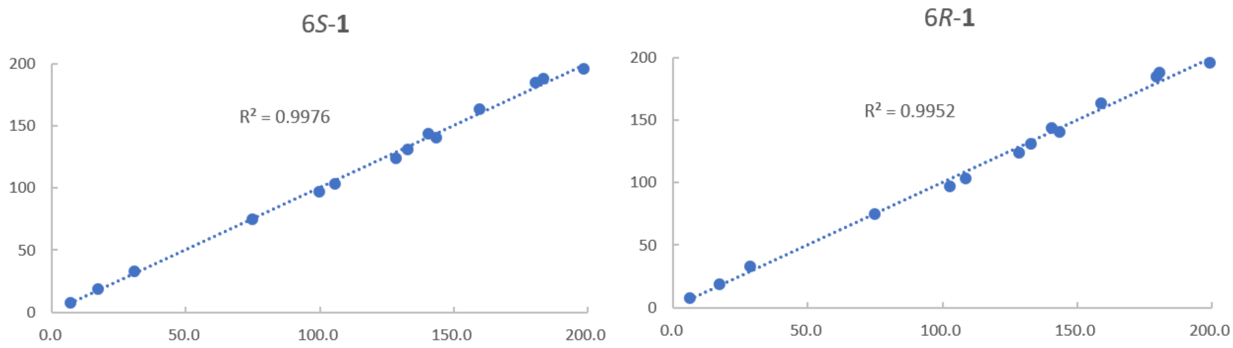

Supplementary Figure S35. Regression analysis of the experimental *vs* calculated ^{13}C NMR chemical shifts of **1** at the B3LYP/6-311+G(d,p) level in $CDCl_3$.

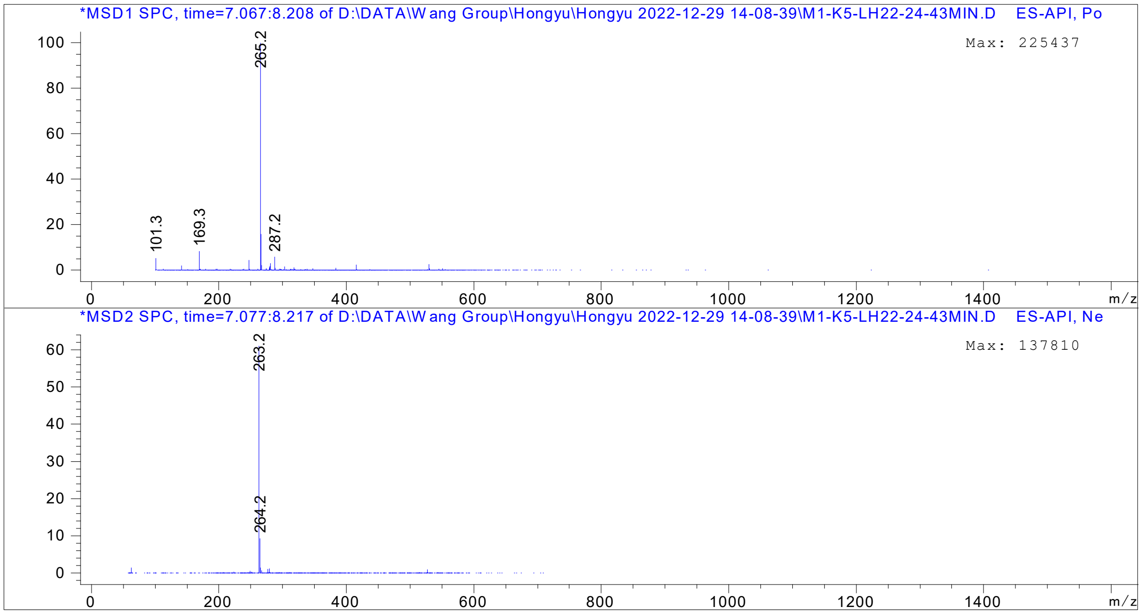

Supplementary Figure S36. (+) and (–)-ESI-MS of compound **2**.

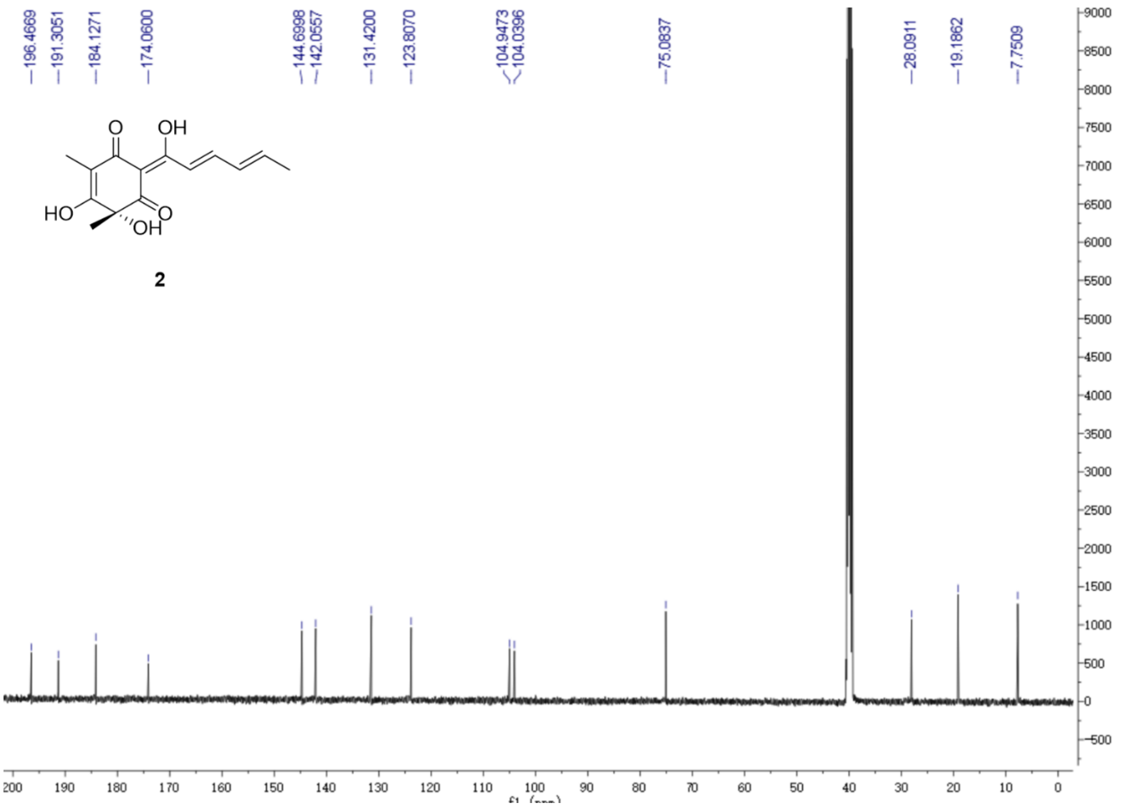

Supplementary Figure S37. ^{13}C NMR (125 MHz, DMSO-d_6) spectrum of compound **2**.

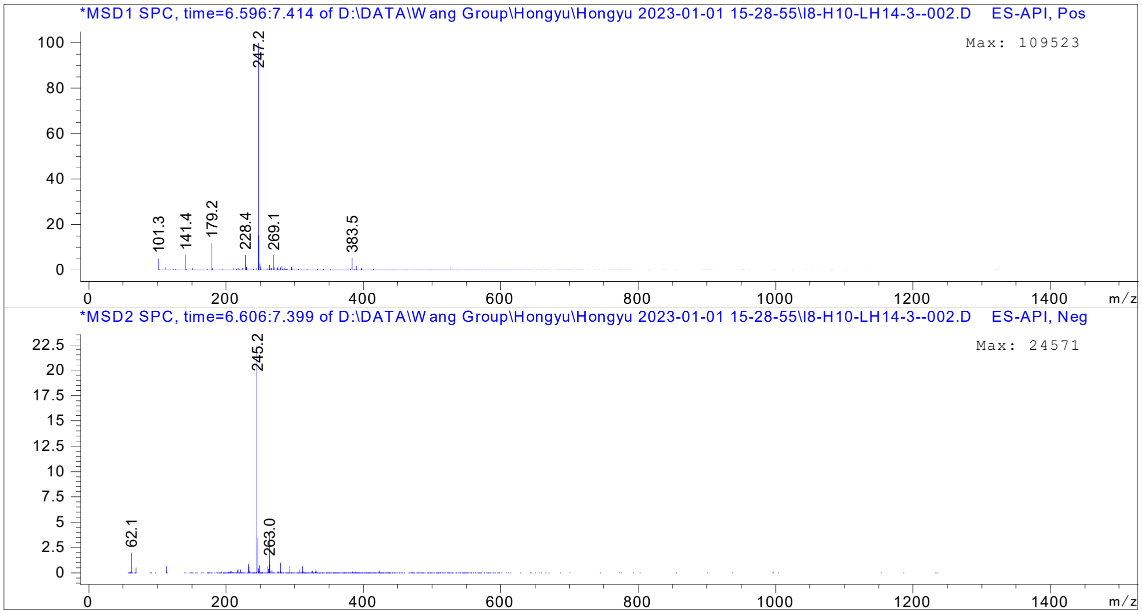

Supplementary Figure S38. (+) and (–)-ESI-MS of compound **3**.

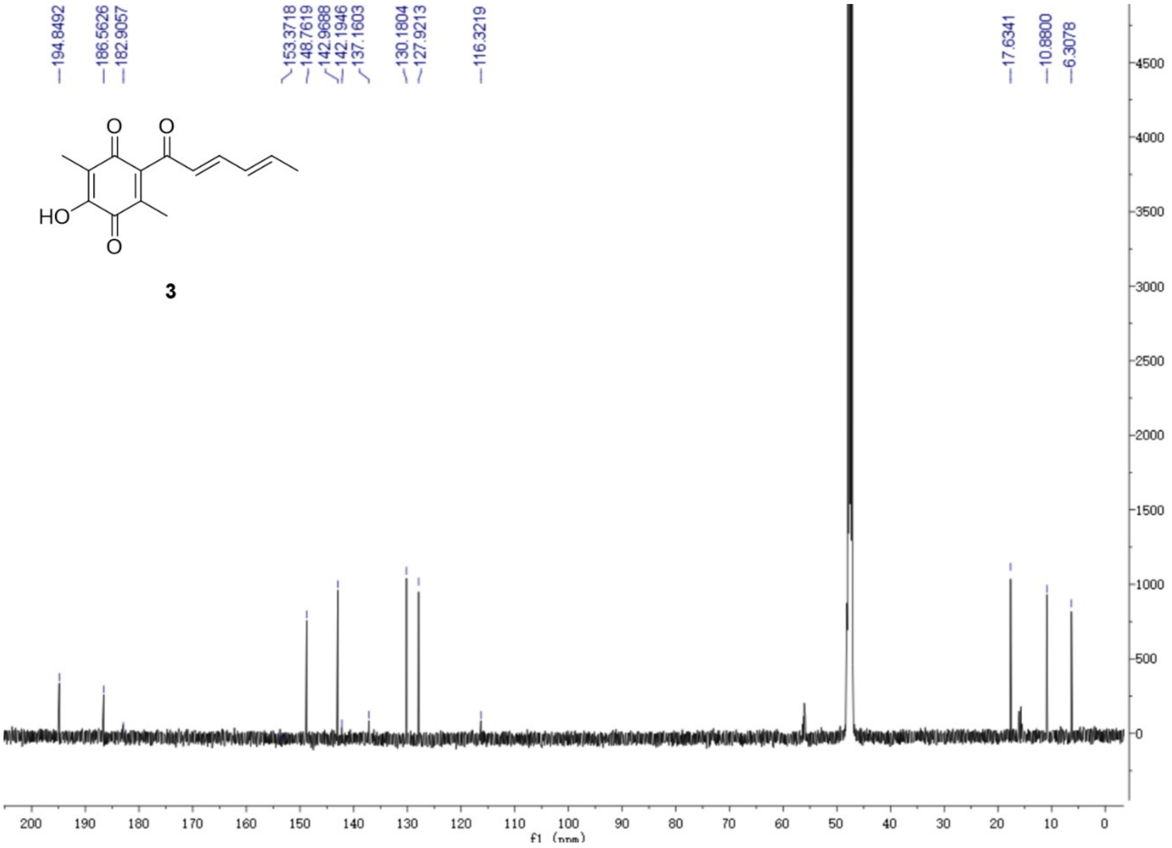

Supplementary Figure S39. ^{13}C NMR (125 MHz, CD_3OD) spectrum of compound **3**.

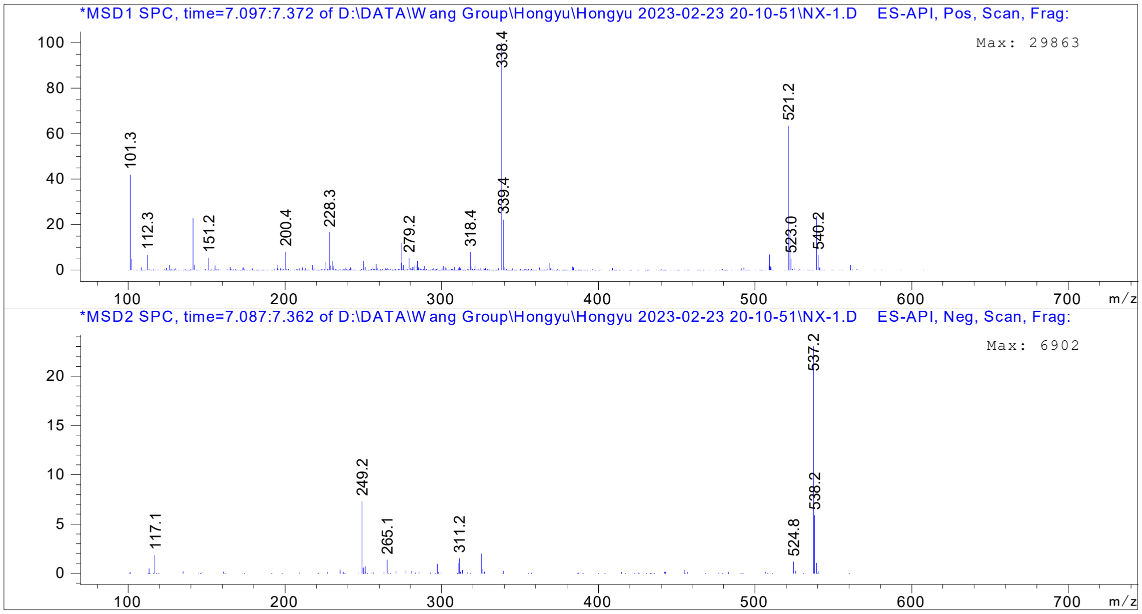

Supplementary Figure S40. (+) and (–)-ESI-MS of compound **4**.

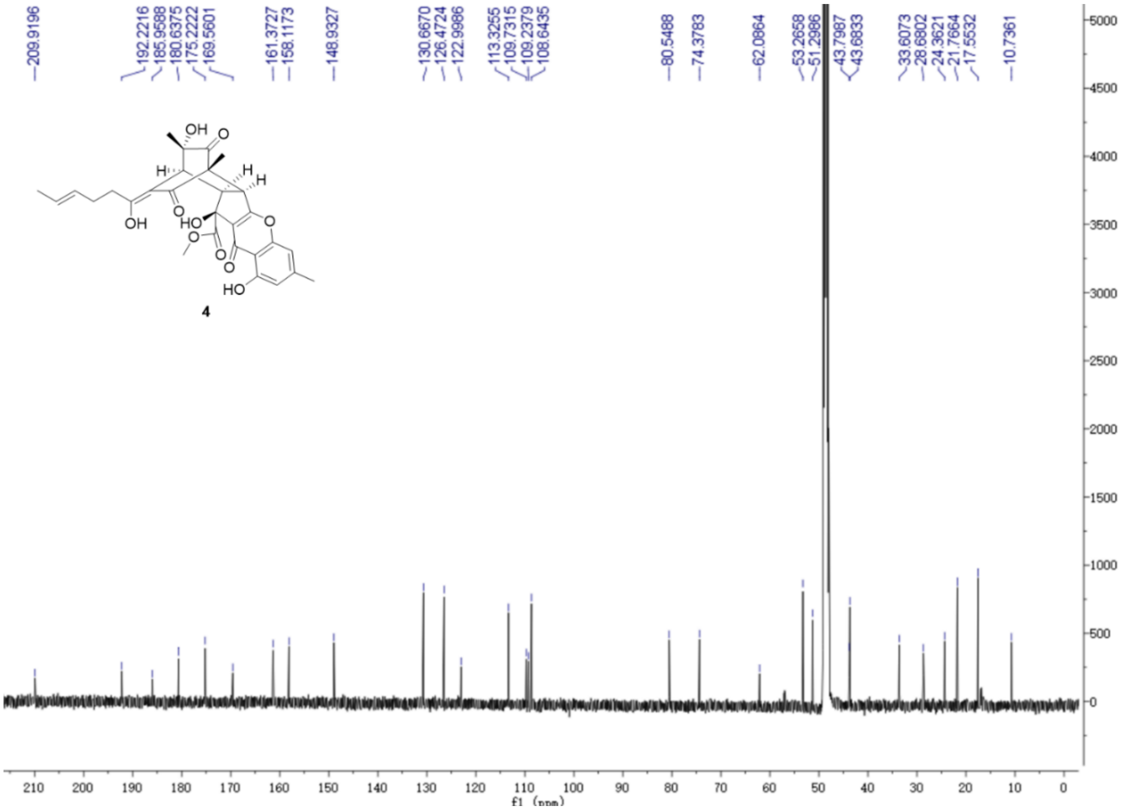

Supplementary Figure S41. ^{13}C NMR (125 MHz, CD_3OD) spectrum of compound **4**.

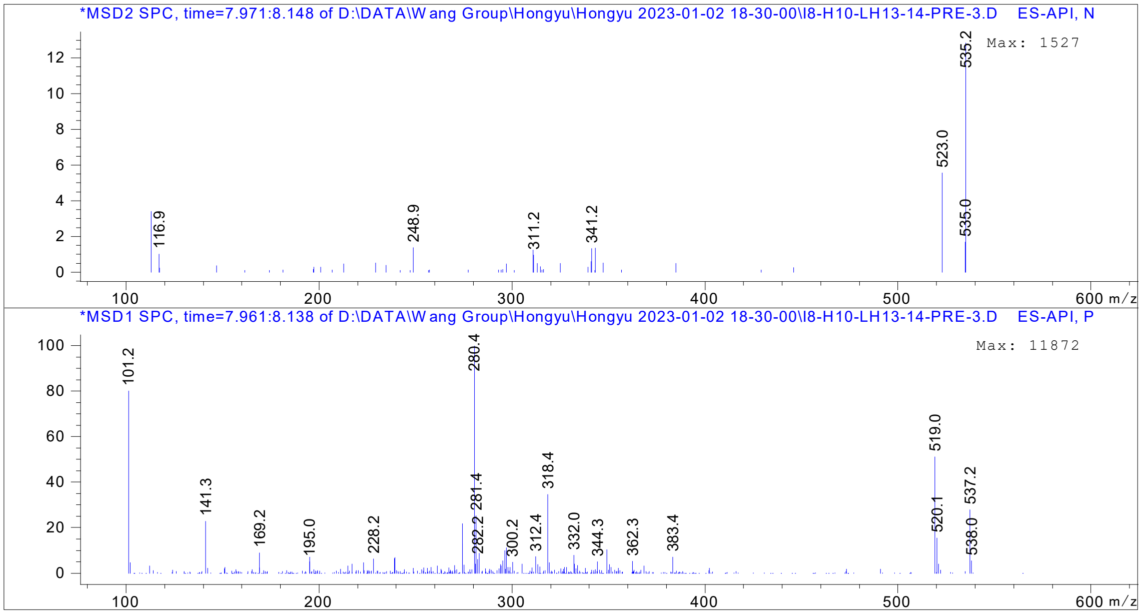

Supplementary Figure S42. (+) and (–)-ESI-MS of compound **5**.

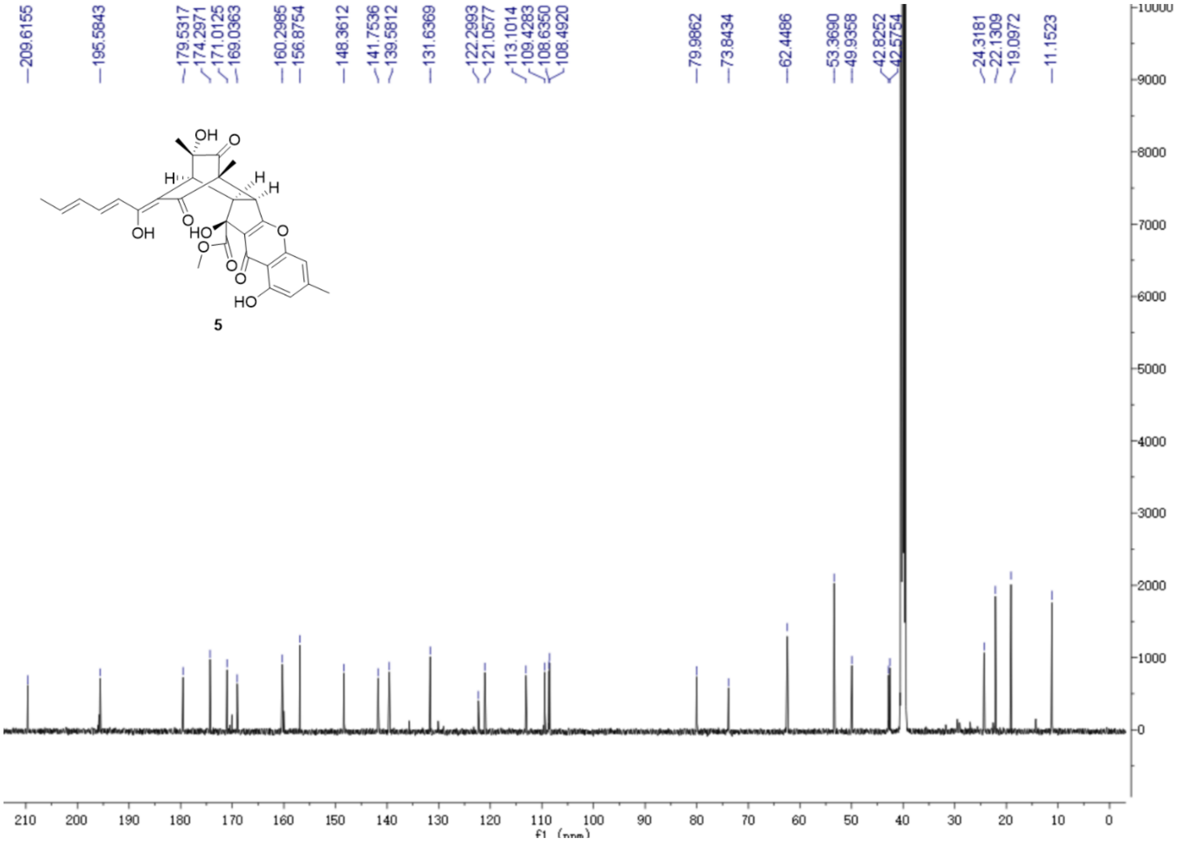

Supplementary Figure S43. ^{13}C NMR (125 MHz, DMSO-d_6) spectrum of compound **5**.

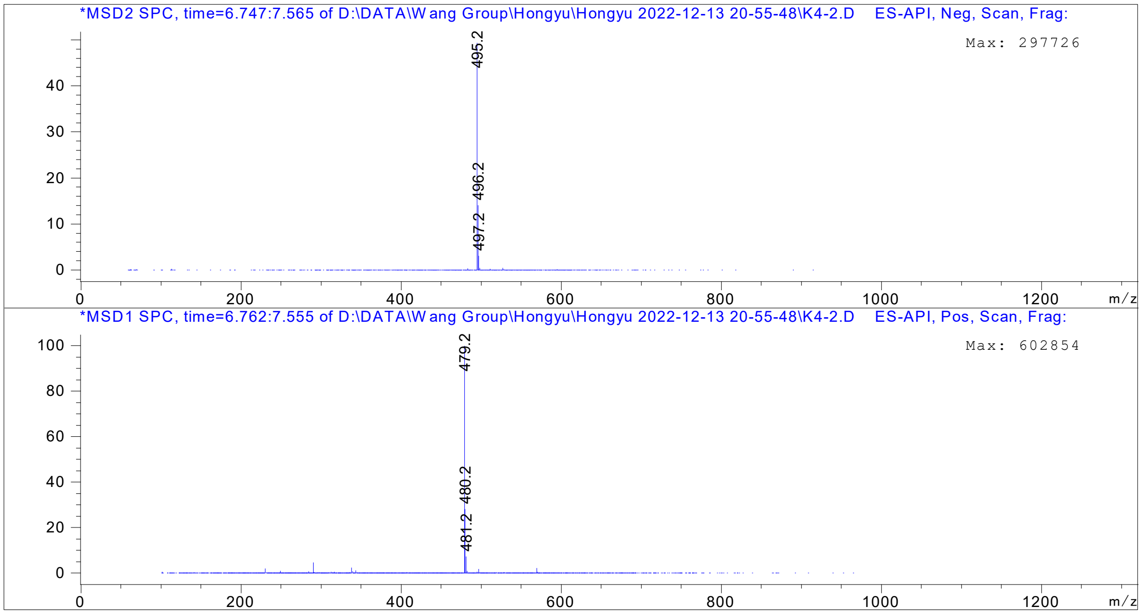

Supplementary Figure S44. (+) and (–)-ESI-MS of compound **6**.

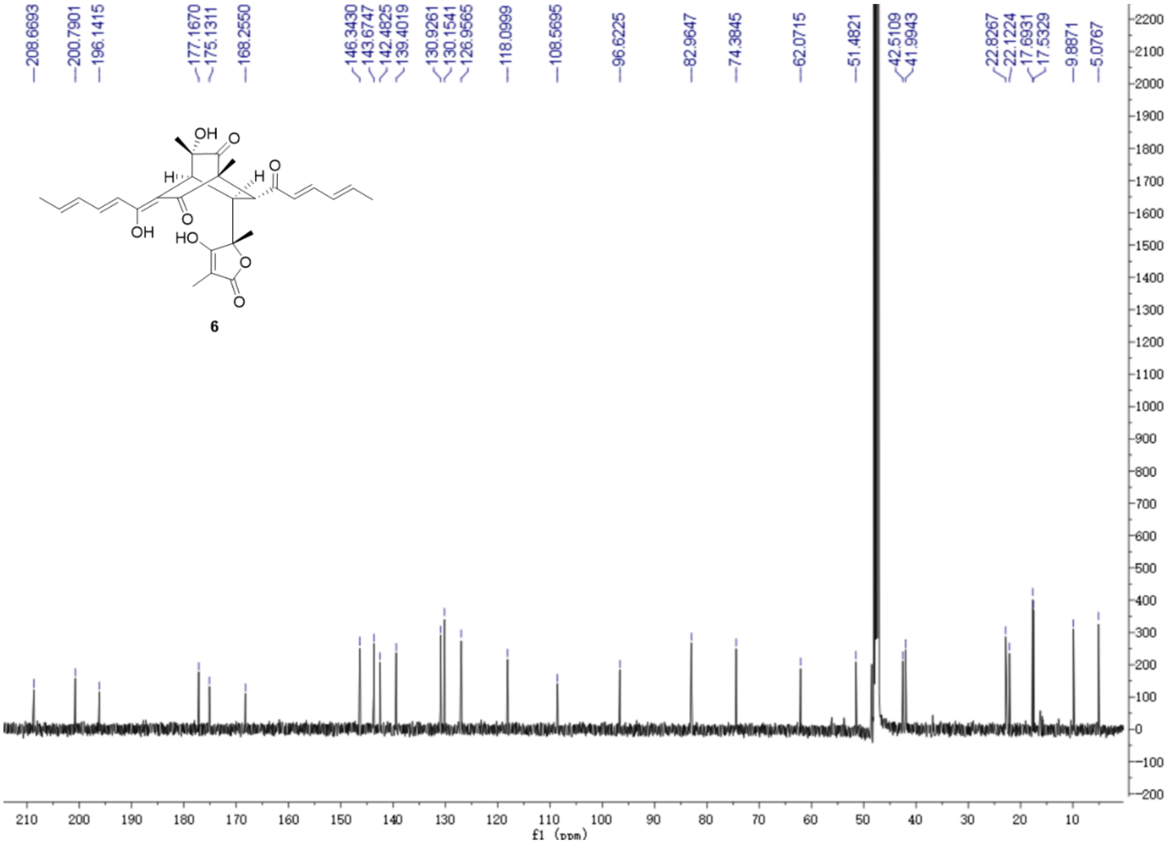

Supplementary Figure S45. ^{13}C NMR (125 MHz, CD_3OD) spectrum of compound **6**.

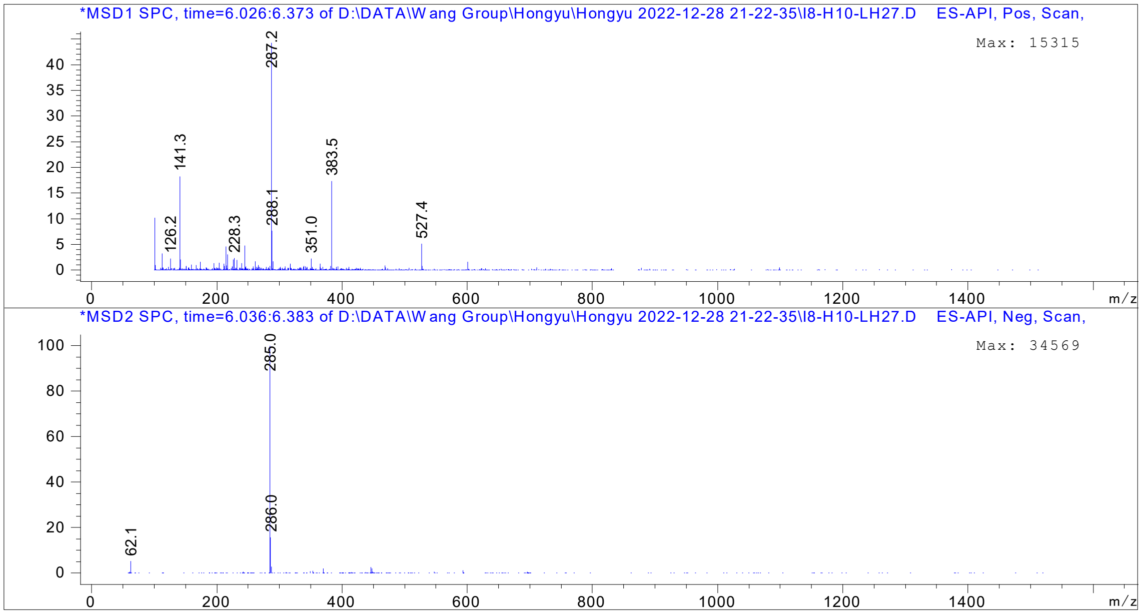

Supplementary Figure S46. (+) and (–)-ESI-MS of compound **7**.

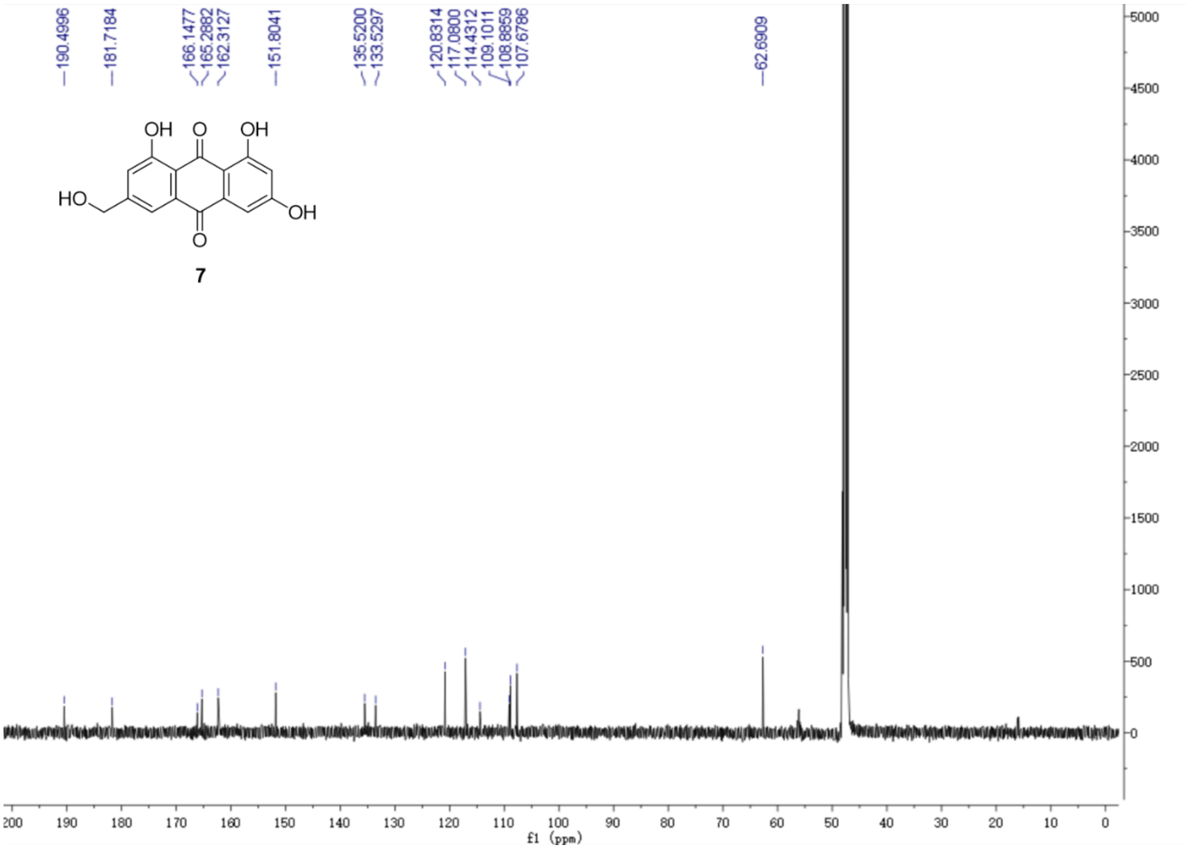

Supplementary Figure S47. ^{13}C NMR (125 MHz, CD_3OD) spectrum of compound **7**.

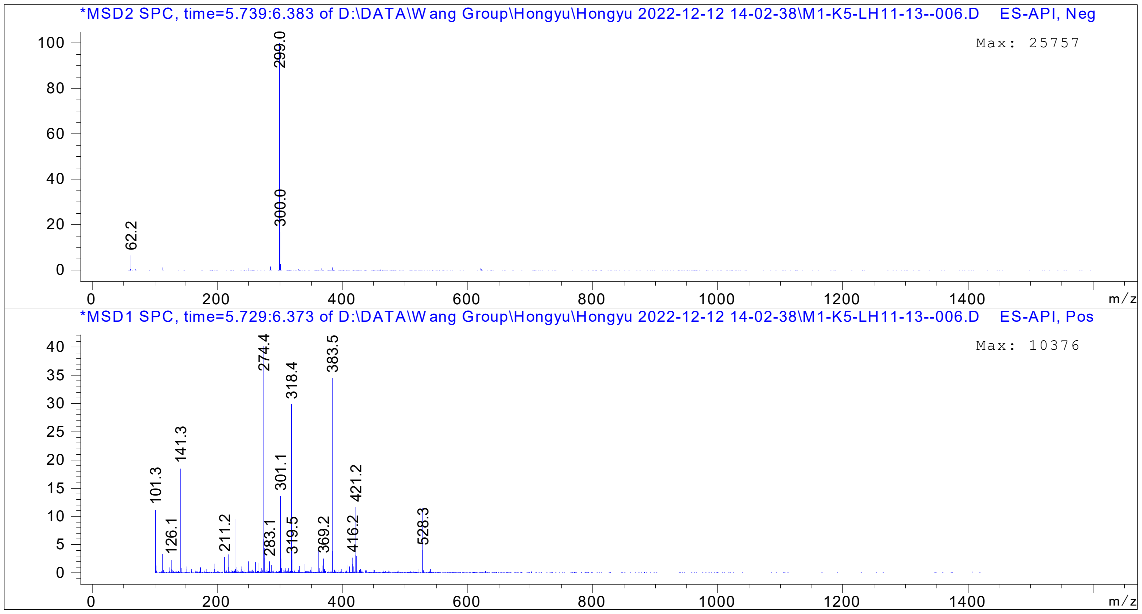

Supplementary Figure S48. (+) and (–)-ESI-MS of compound **8**.

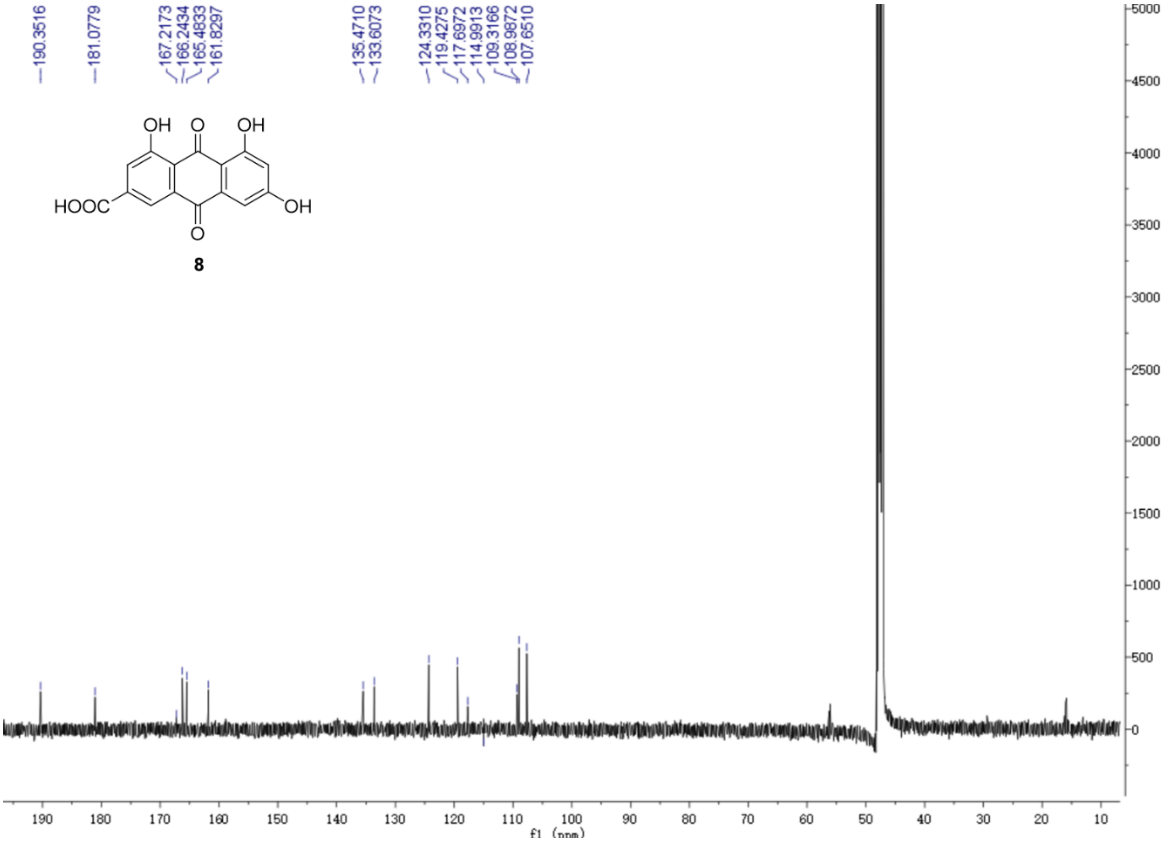

Supplementary Figure S49. ^{13}C NMR (125 MHz, CD_3OD) spectrum of compound **8**.

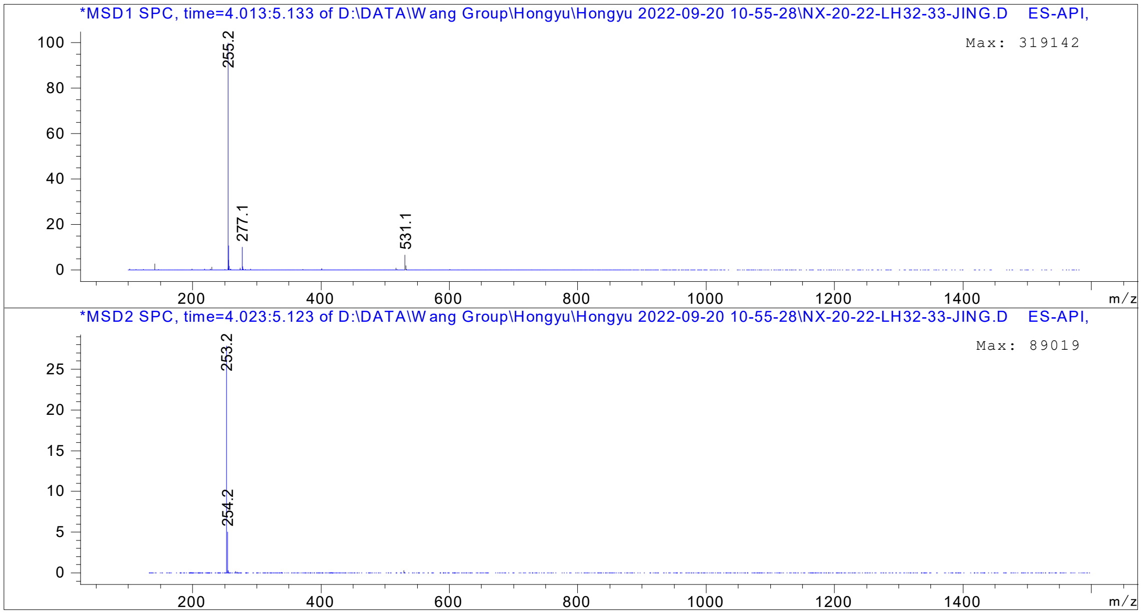

Supplementary Figure S50. (+) and (–)-ESI-MS of compound **9**.

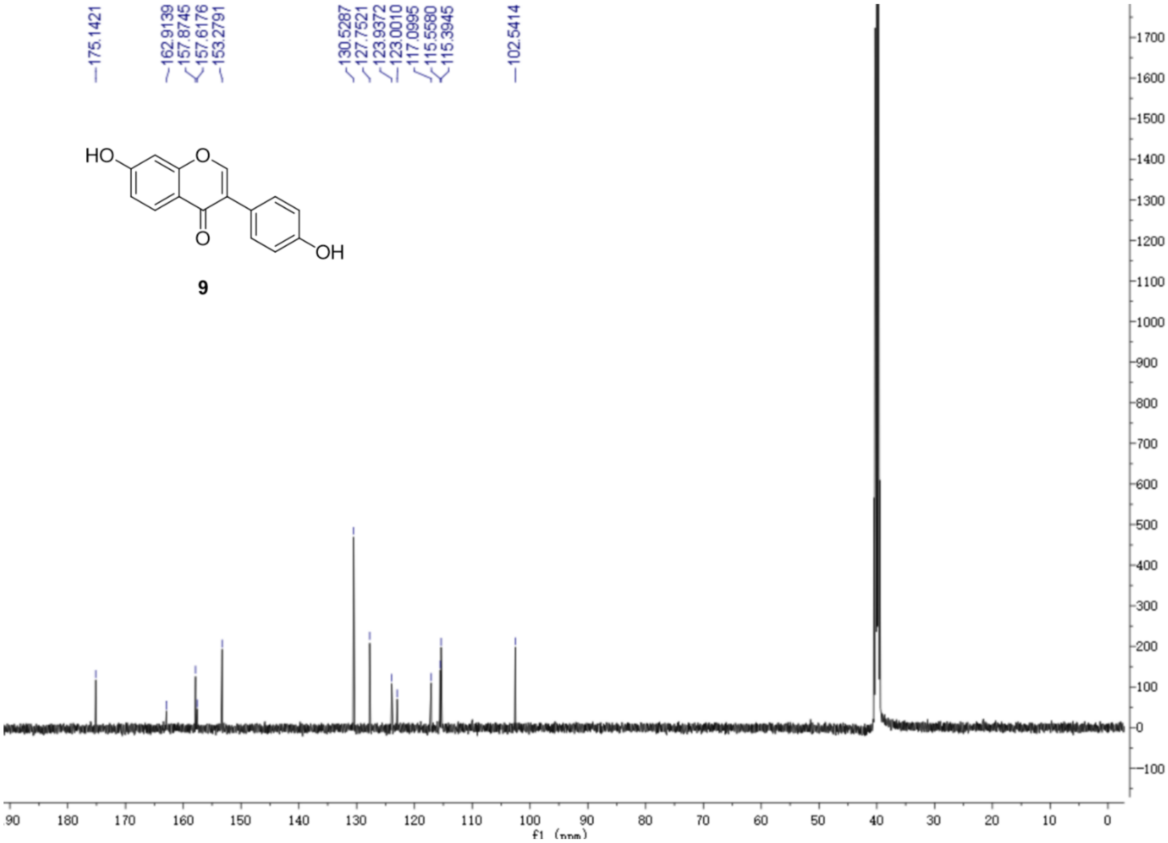

Supplementary Figure S51. ^{13}C NMR (125 MHz, DMSO-d_6) spectrum of compound **9**.

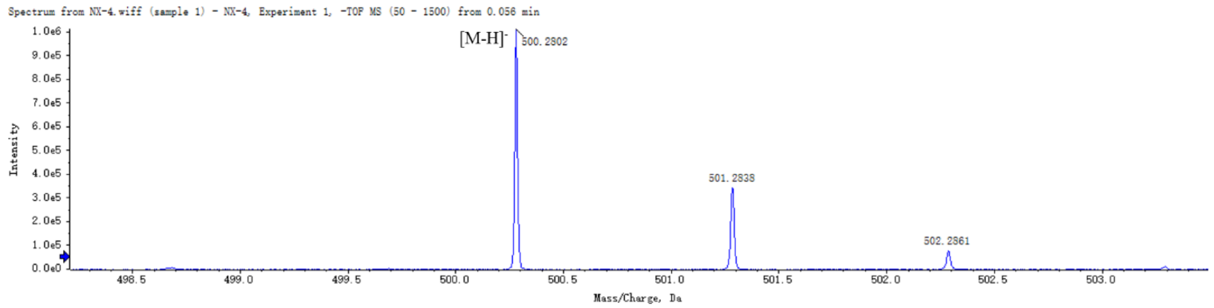

Supplementary Figure S52. (–)-HRESI-MS of compound **10**.

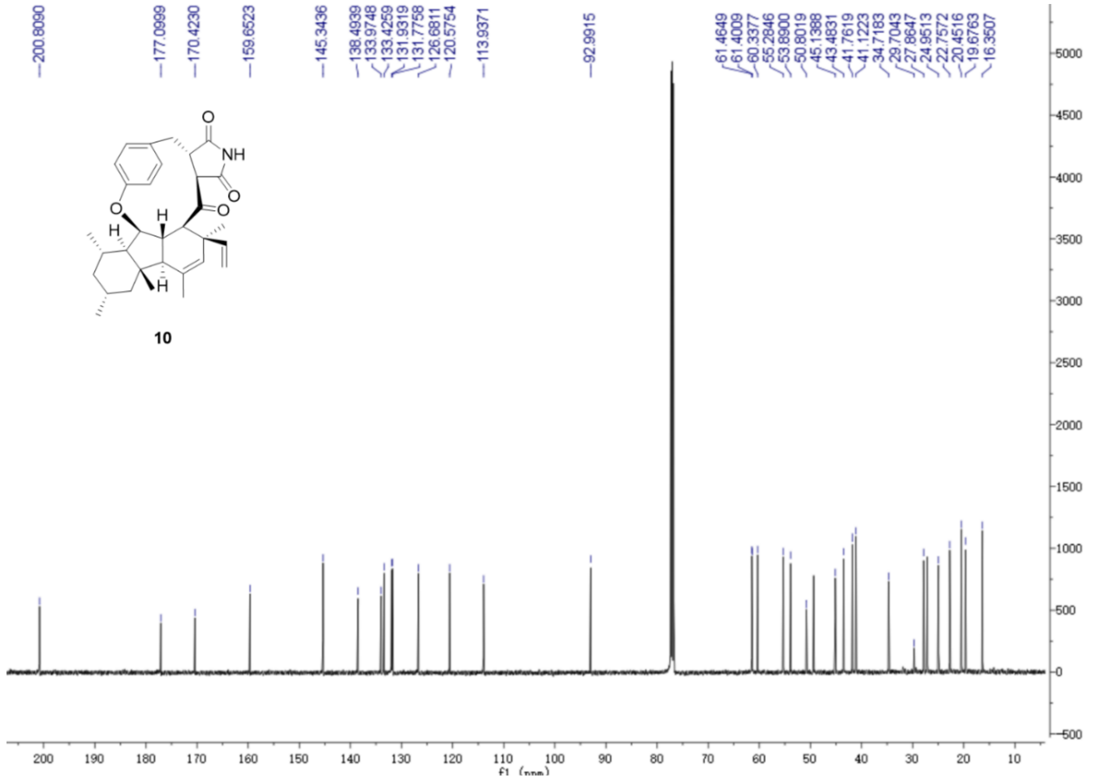

Supplementary Figure S53. ^{13}C NMR (125 MHz, CD_3OD) spectrum of compound **10**.

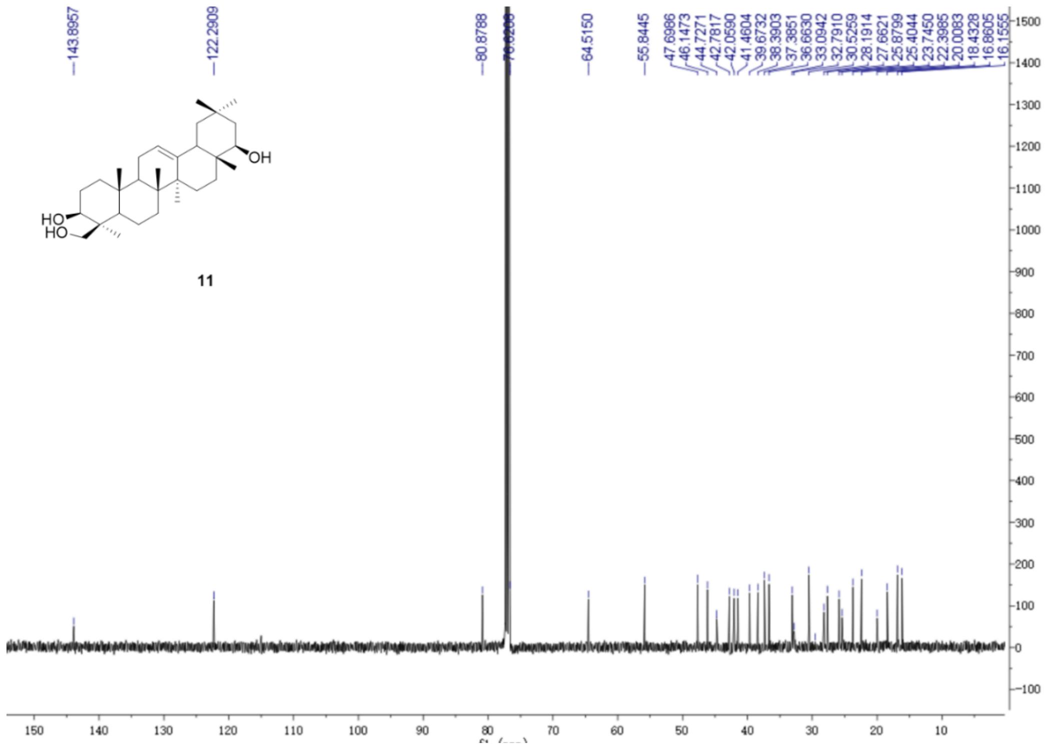

Supplementary Figure S54. ^{13}C NMR (125 MHz, CD_3Cl_3) spectrum of compound **11**.

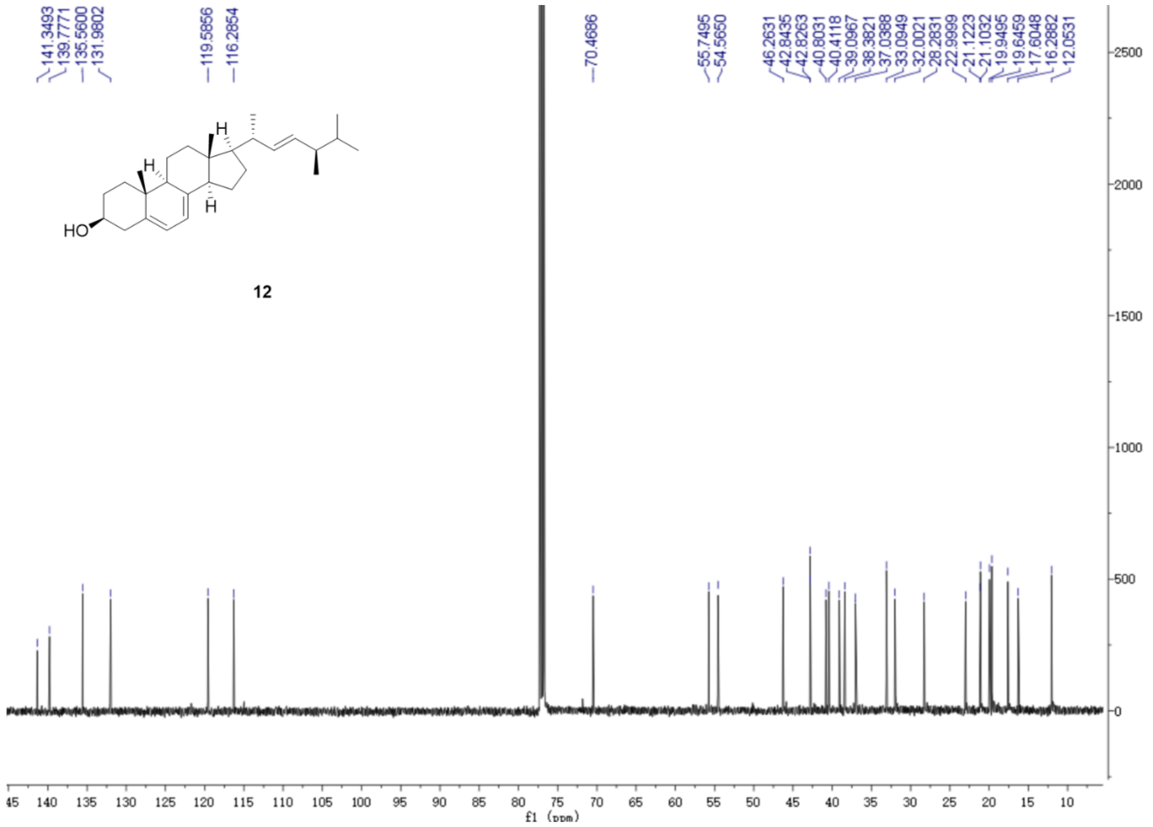

Supplementary Figure S55. ^{13}C NMR (125 MHz, CD_3Cl_3) spectrum of compound **12**.

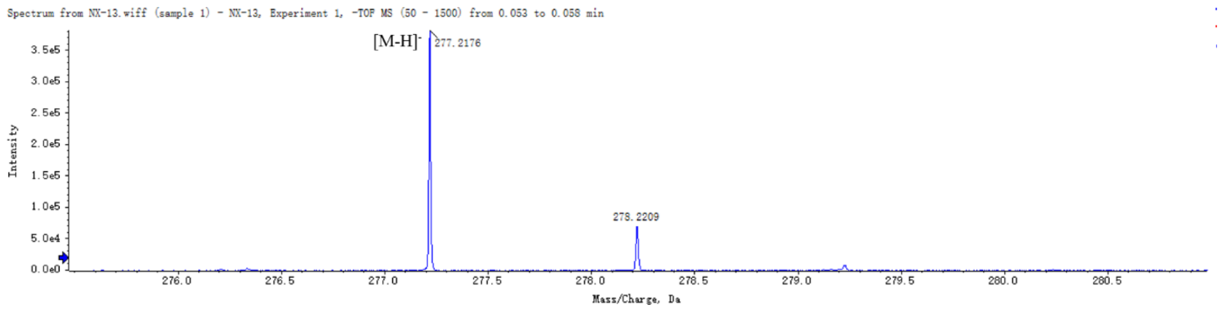

Supplementary Figure S56. (–)-HRESI-MS of compound **13**.

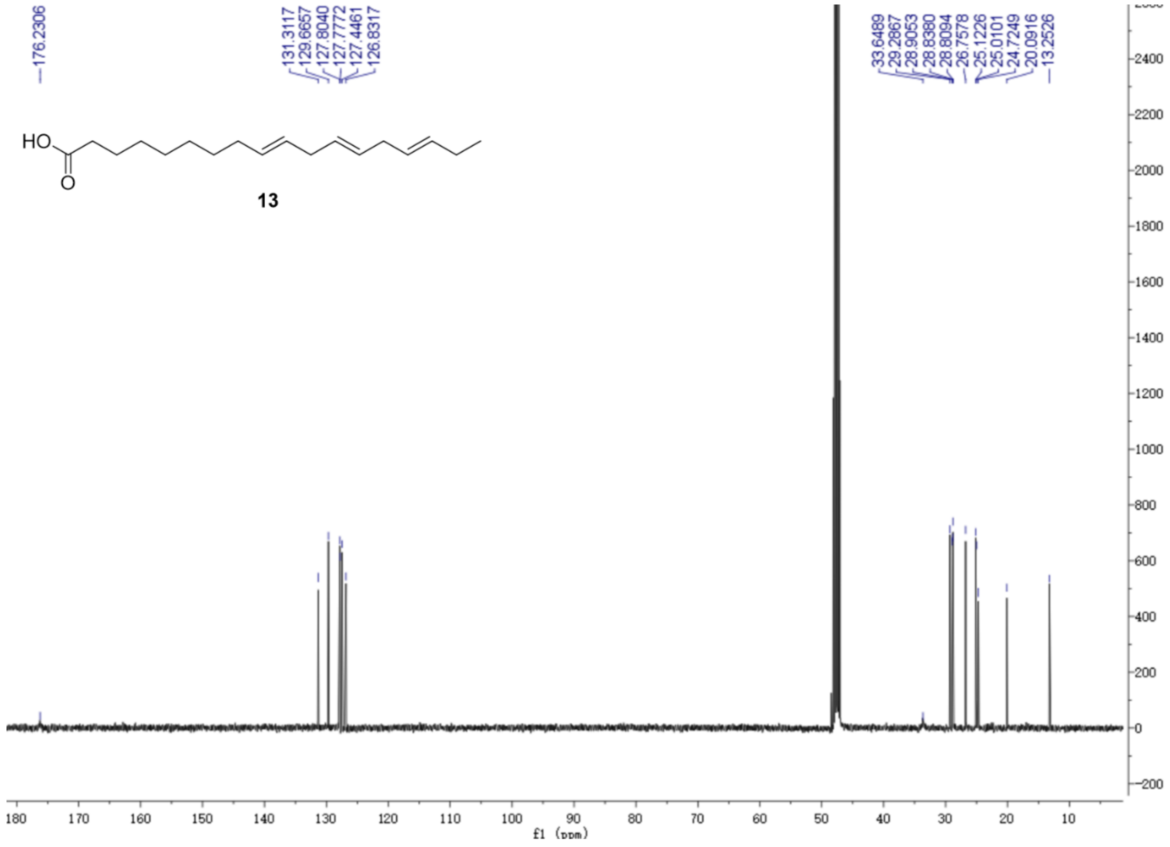

Supplementary Figure S57. ^{13}C NMR (125 MHz, CD_3OD) spectrum of compound **13**.

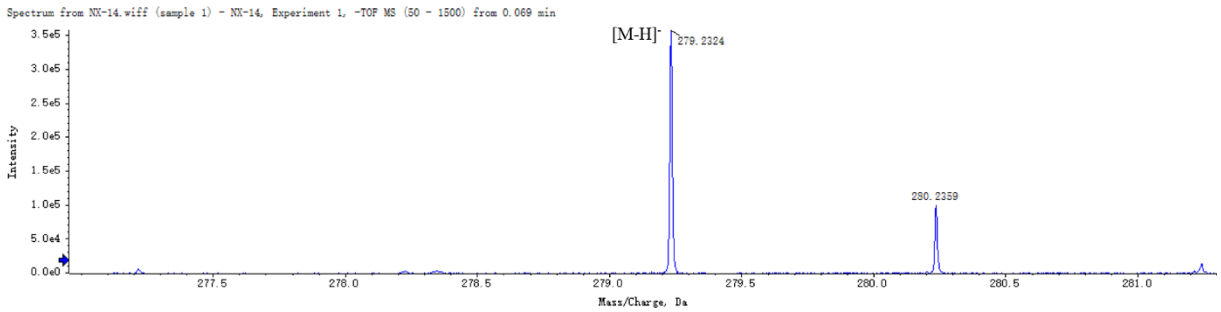

Supplementary Figure S58. (–)-HRESI-MS of compound **14**.

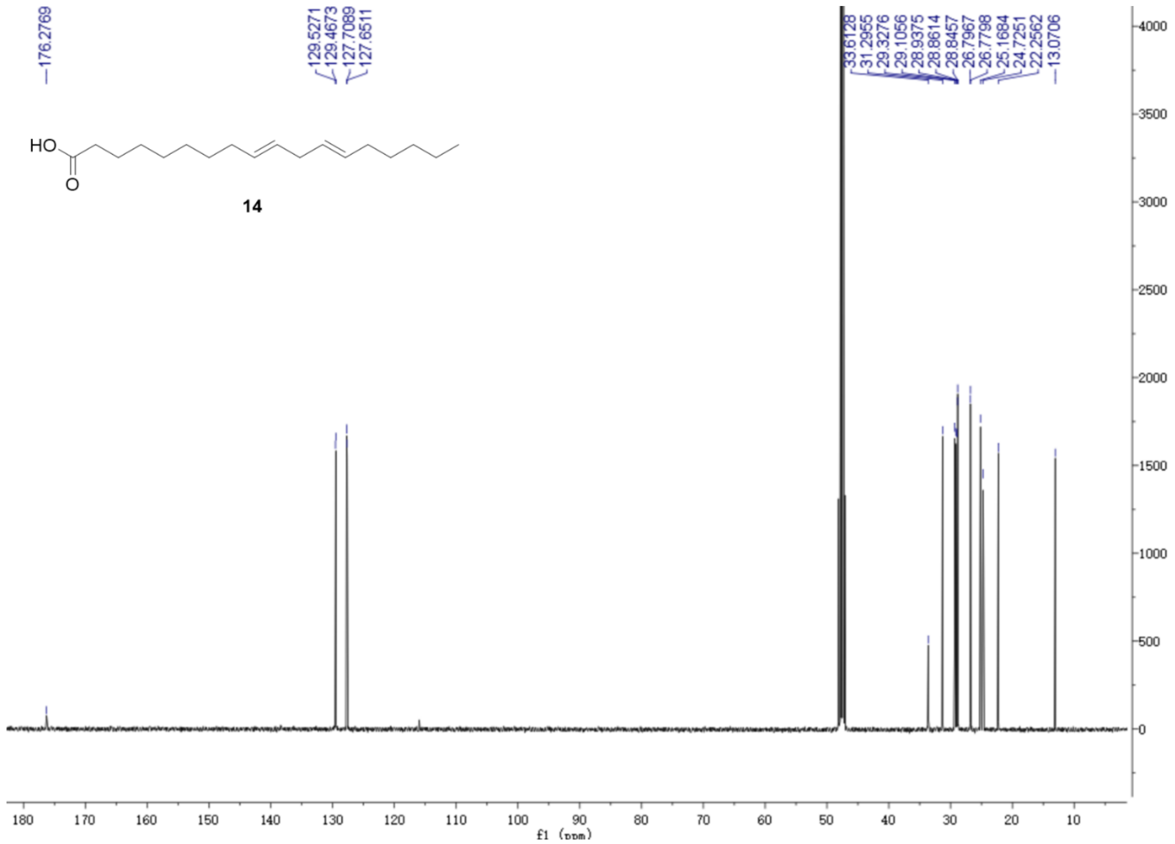

Supplementary Figure S59. ^{13}C NMR (125 MHz, CD_3OD) spectrum of compound **14**.

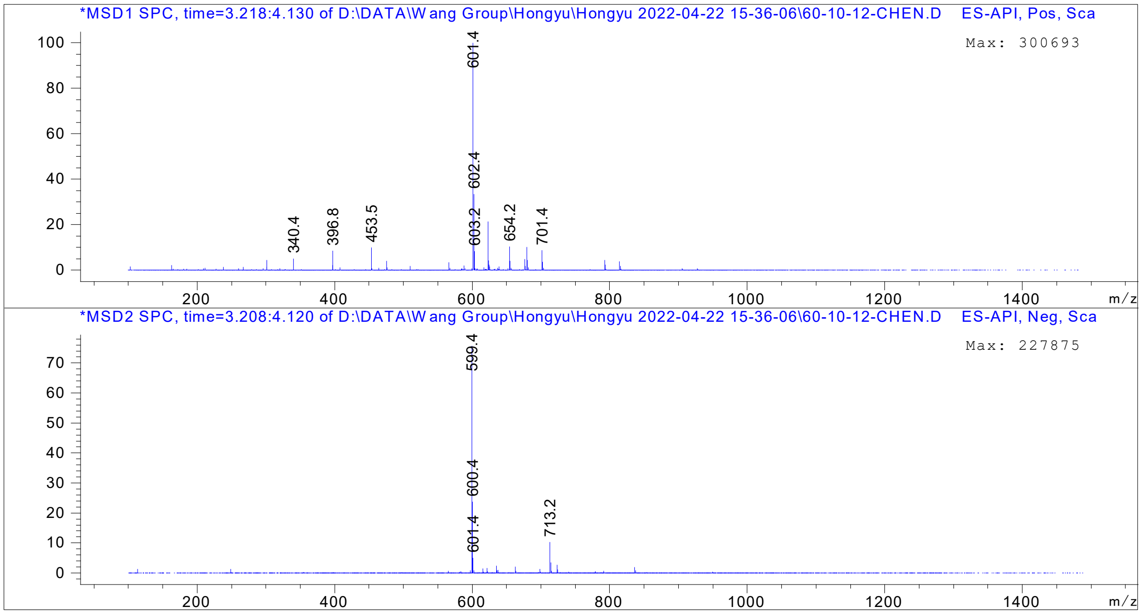

Supplementary Figure S60. (+) and (–)-ESI-MS of compound **15**.

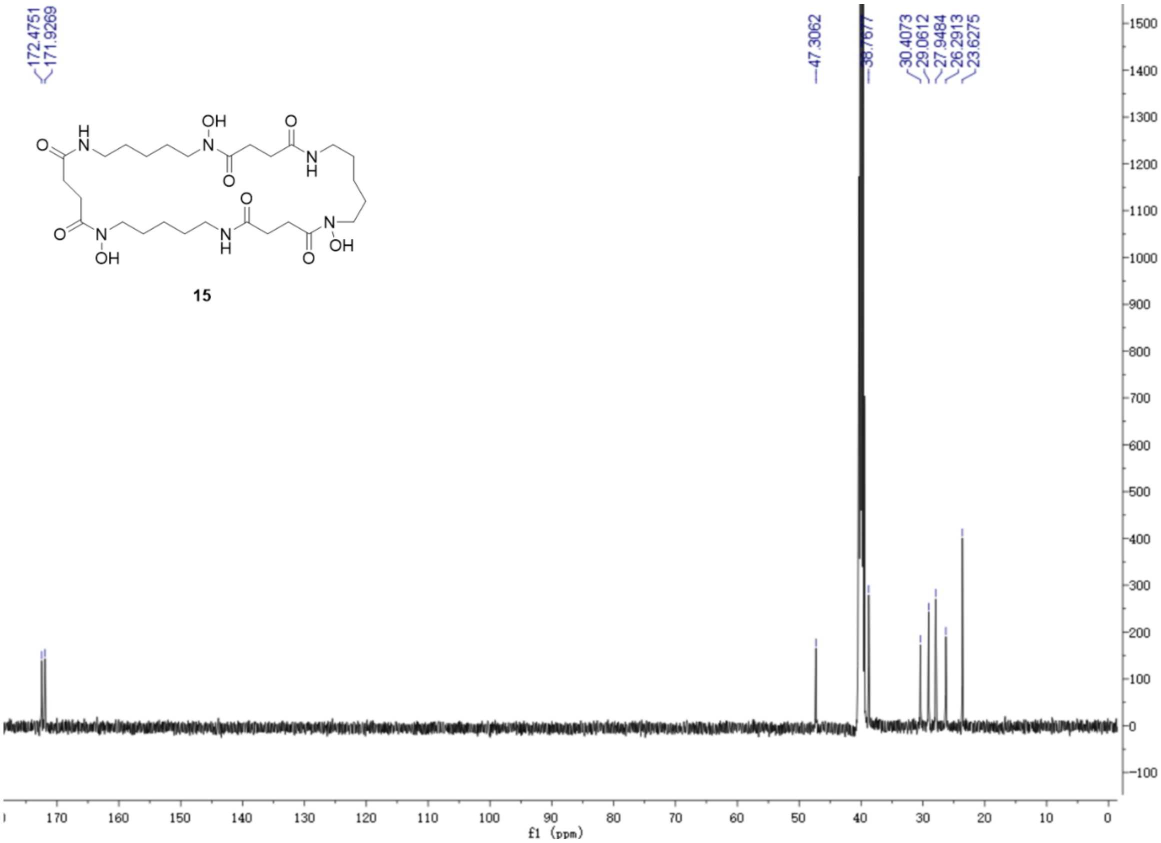

Supplementary Figure S61. ^{13}C NMR (125 MHz, DMSO-d_6) spectrum of compound **15**.

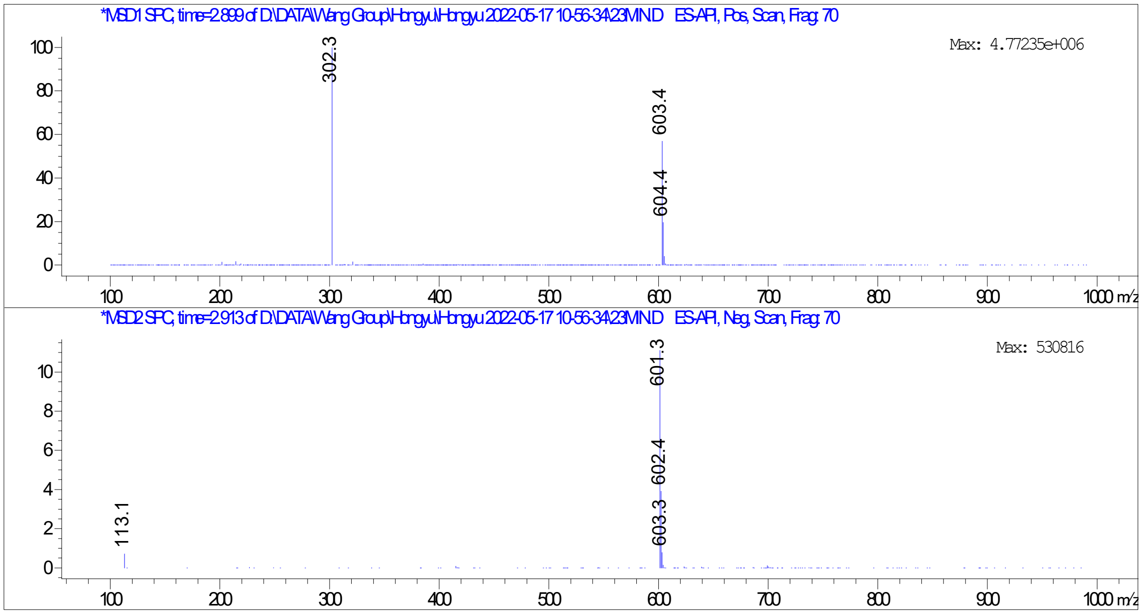

Supplementary Figure S62. (+) and (–)-ESI-MS of compound **16**.

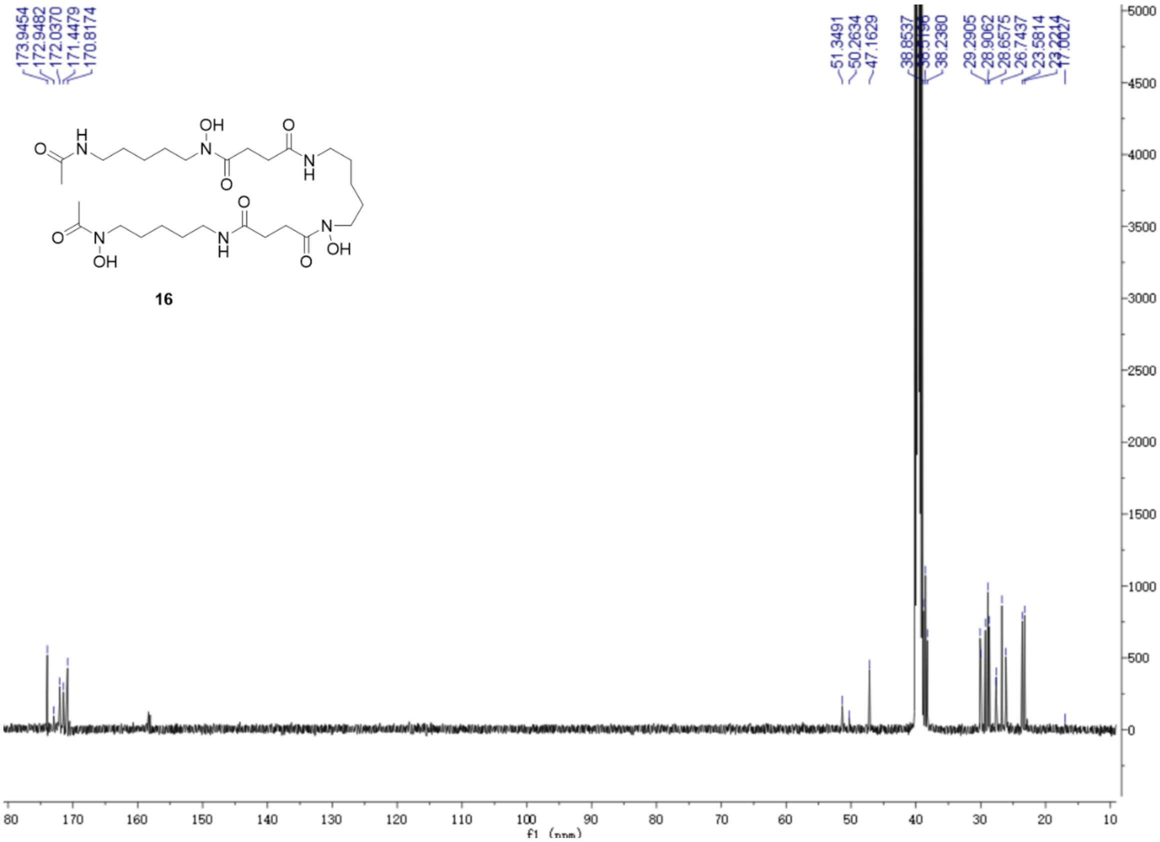

Supplementary Figure S63. ^{13}C NMR (125 MHz, DMSO-d_6) spectrum of compound **16**.

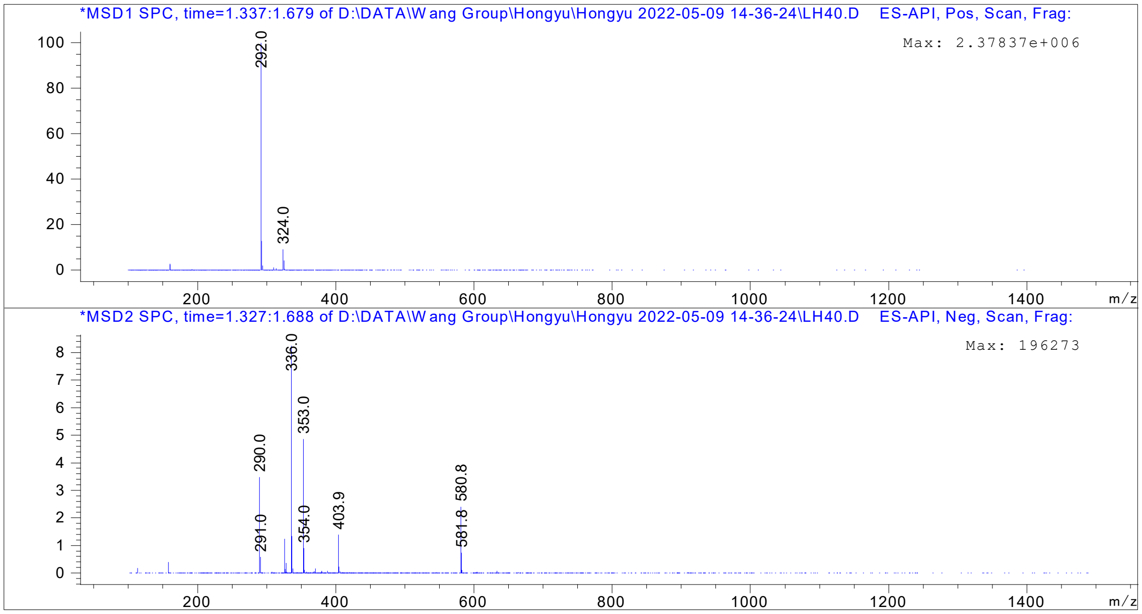

Supplementary Figure S64. (+) and (–)-ESI-MS of compound **17**.

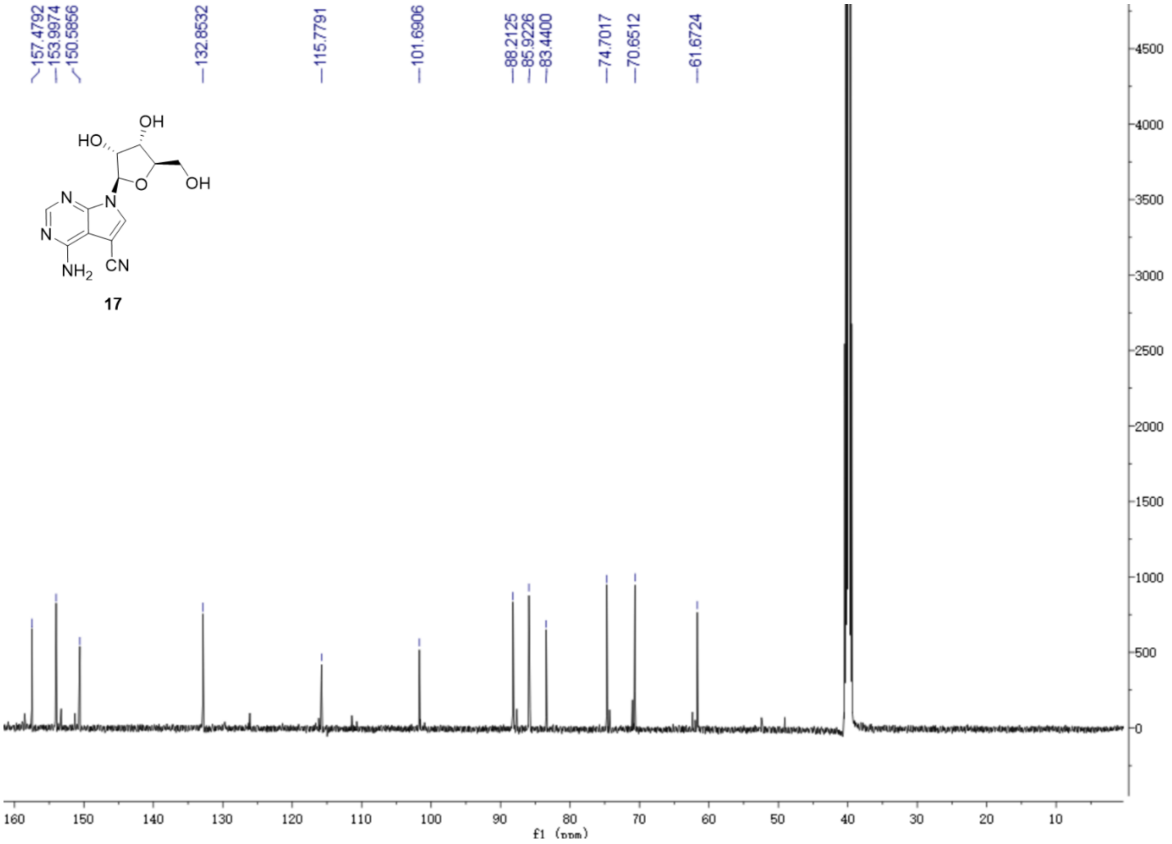

Supplementary Figure S65. ^{13}C NMR (125 MHz, DMSO-d_6) spectrum of compound **17**.

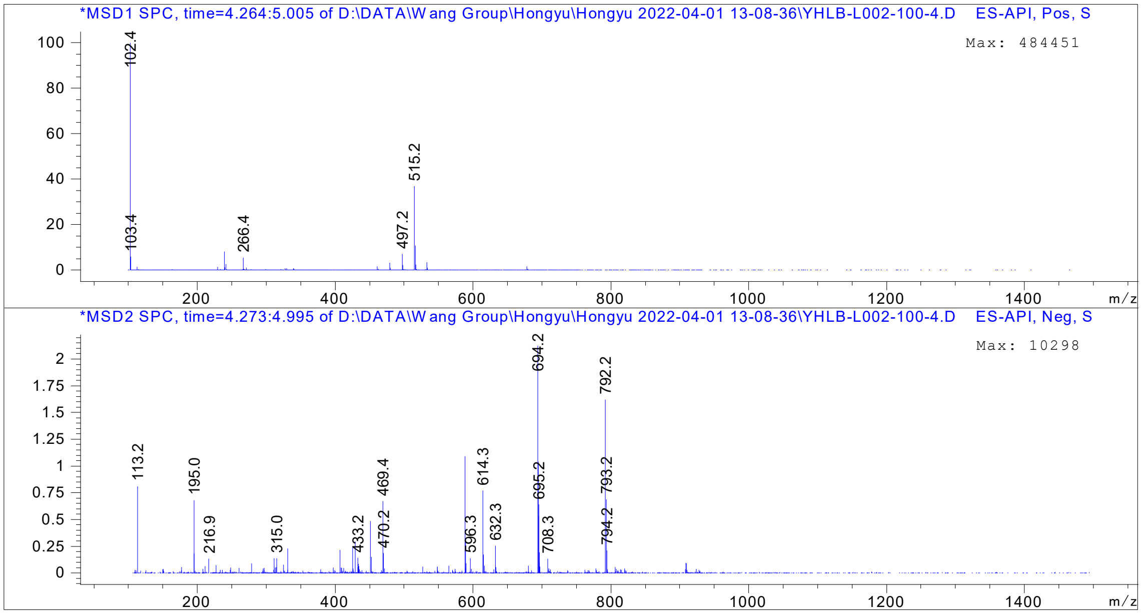

Supplementary Figure S66. (+) and (–)-ESI-MS of compound **18**.

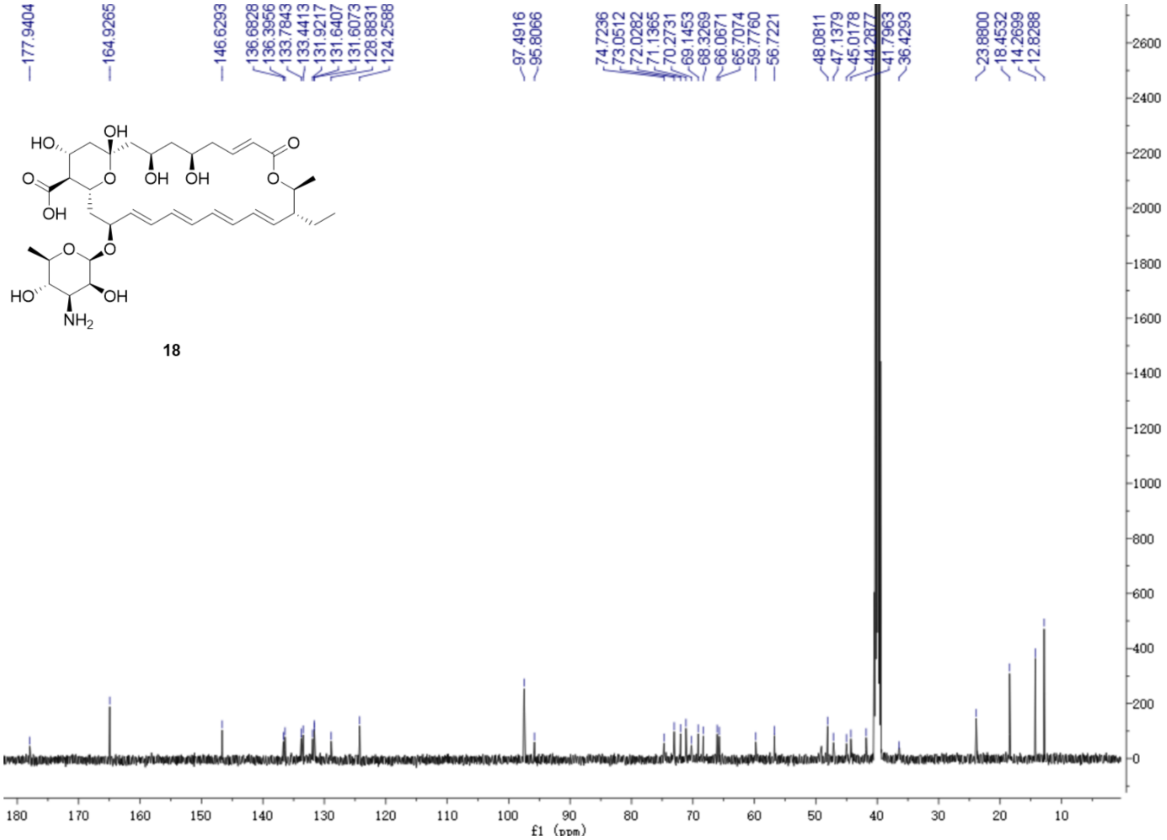

Supplementary Figure S67. ^{13}C NMR (125 MHz, DMSO-d_6) spectrum of compound **18**.

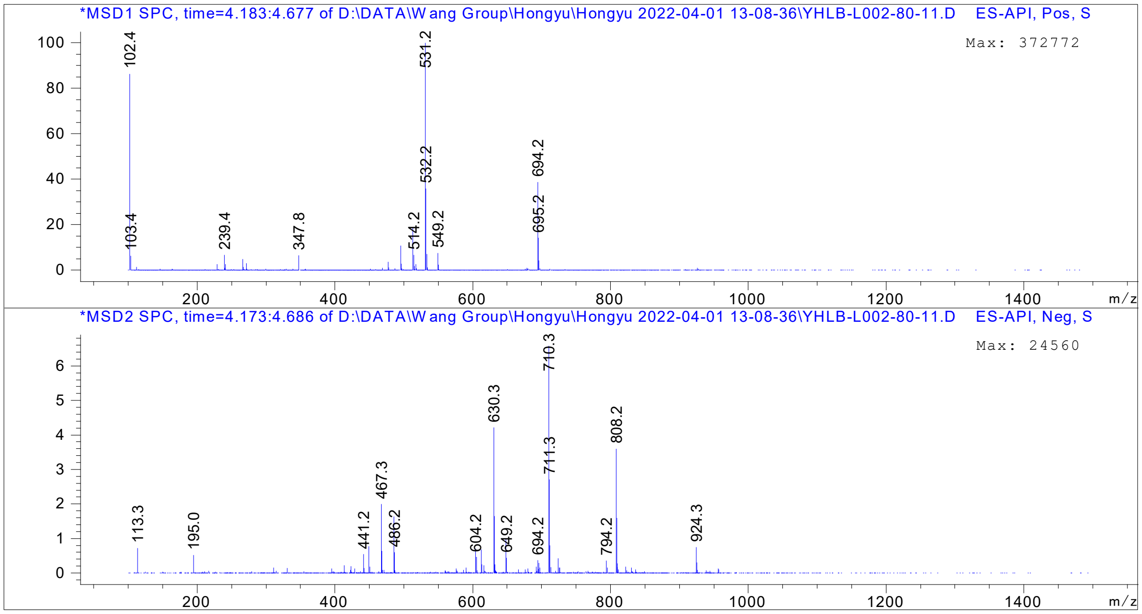

Supplementary Figure S68. (+) and (–)-ESI-MS of compound **19**.

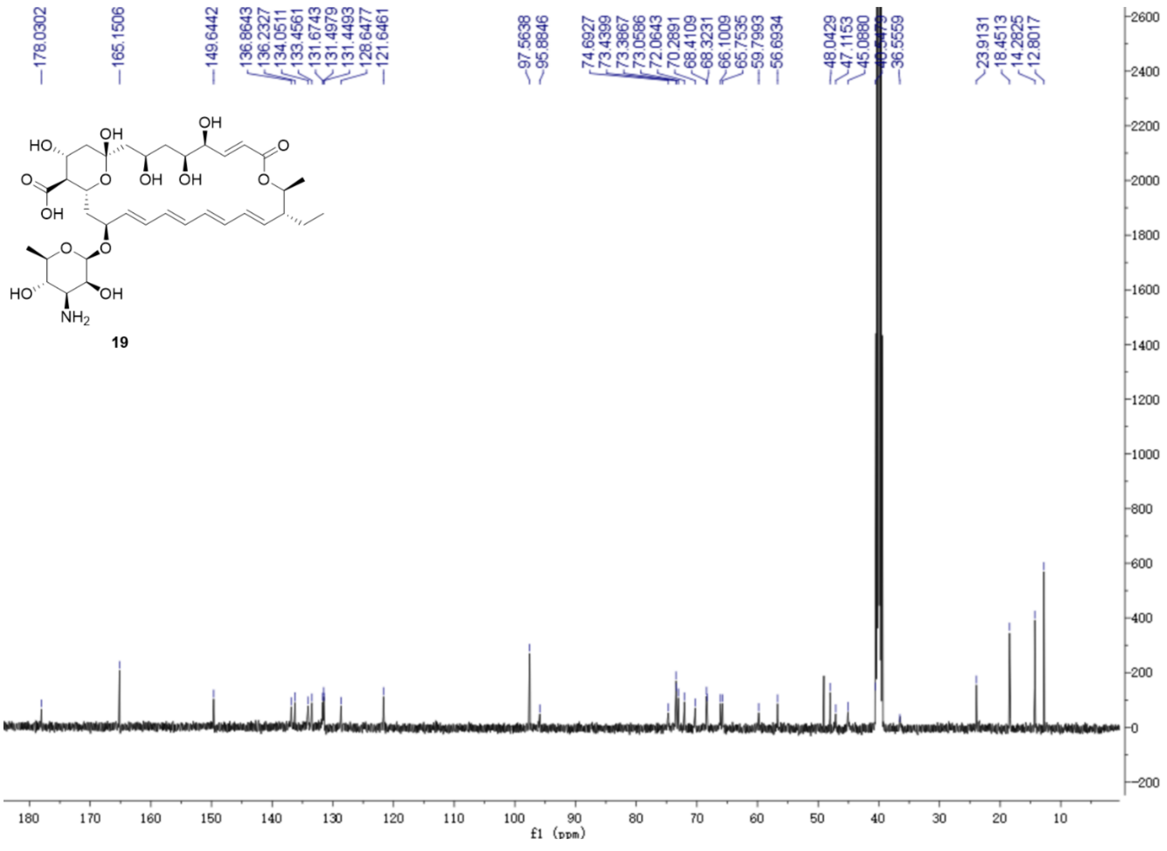

Supplementary Figure S69. ^{13}C NMR (125 MHz, DMSO-d_6) spectrum of compound **19**.

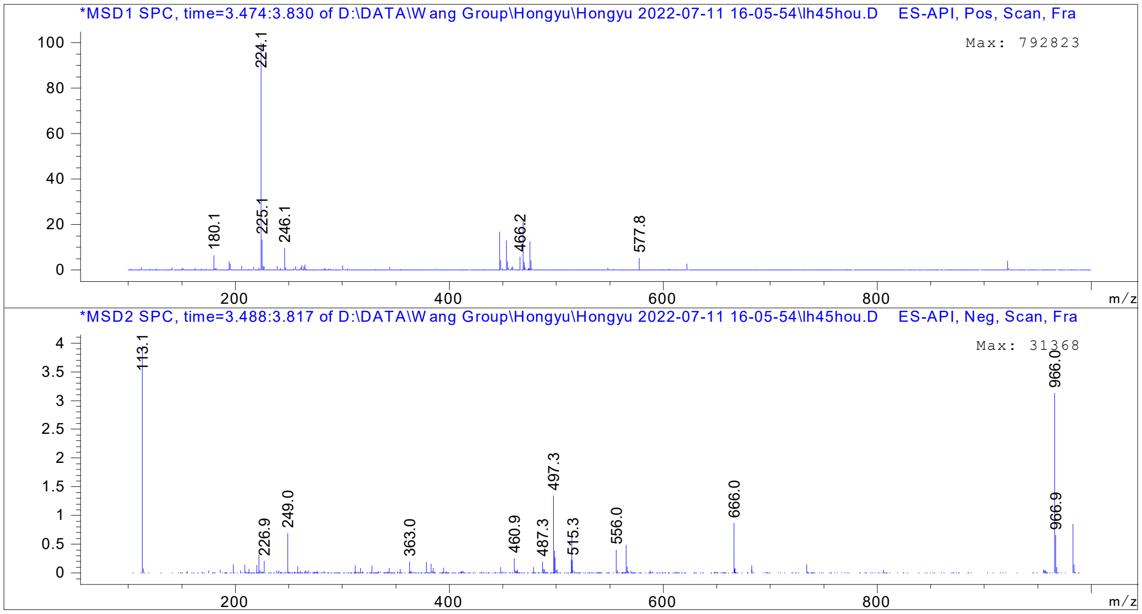

Supplementary Figure S70. (+) and (–)-ESI-MS of compound **20**.

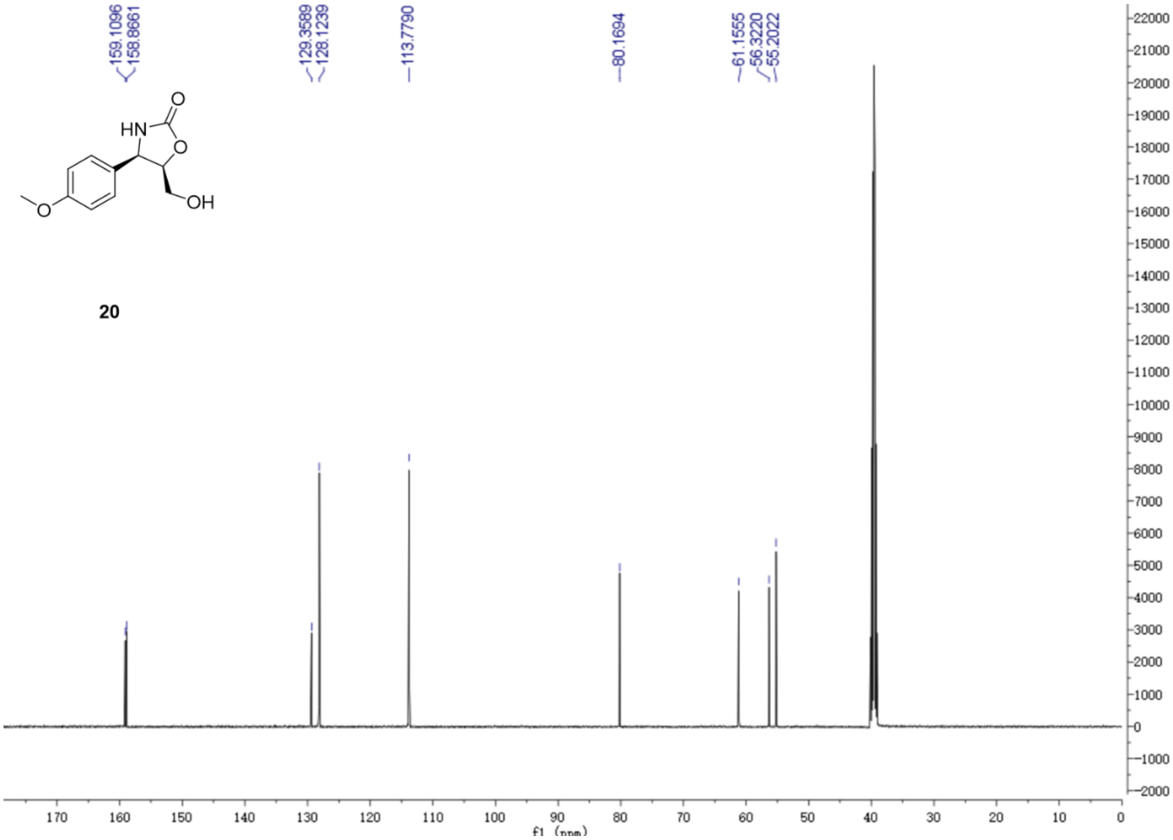

Supplementary Figure S71. ^{13}C NMR (125 MHz, DMSO-d_6) spectrum of compound **20**.

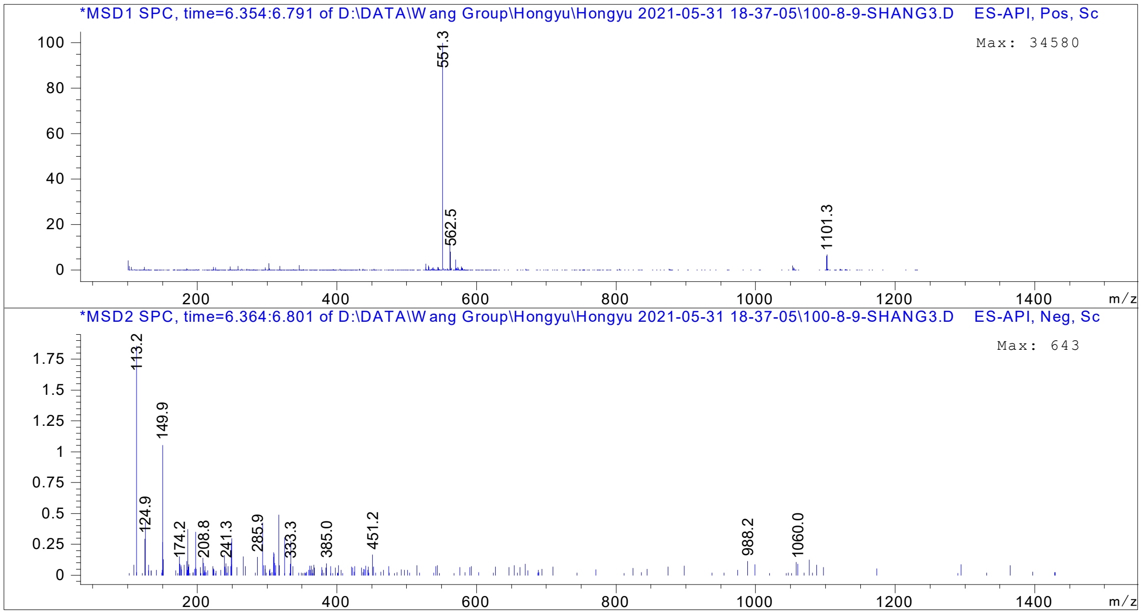

Supplementary Figure S72. (+) and (–)-ESI-MS of compound **21**.

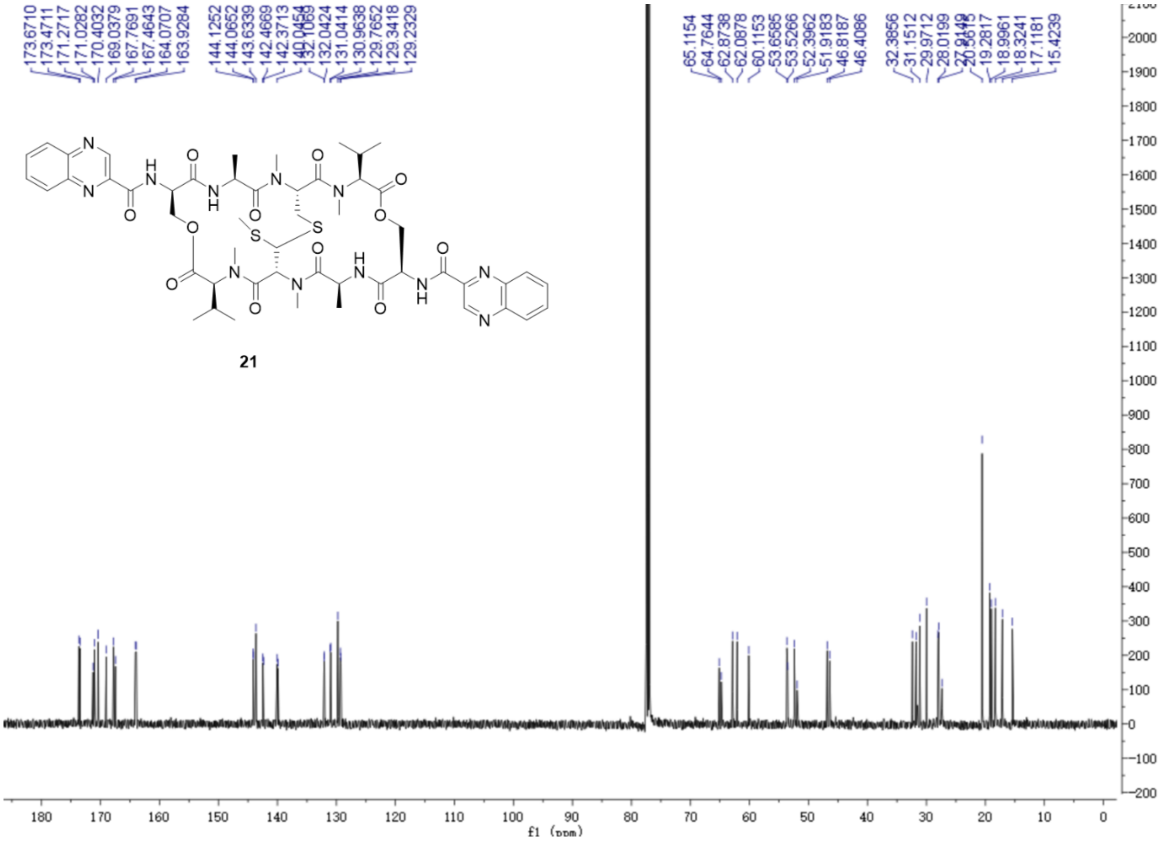

Supplementary Figure S73. ^{13}C NMR (125 MHz, $CDCl_3$) spectrum of compound **21**.

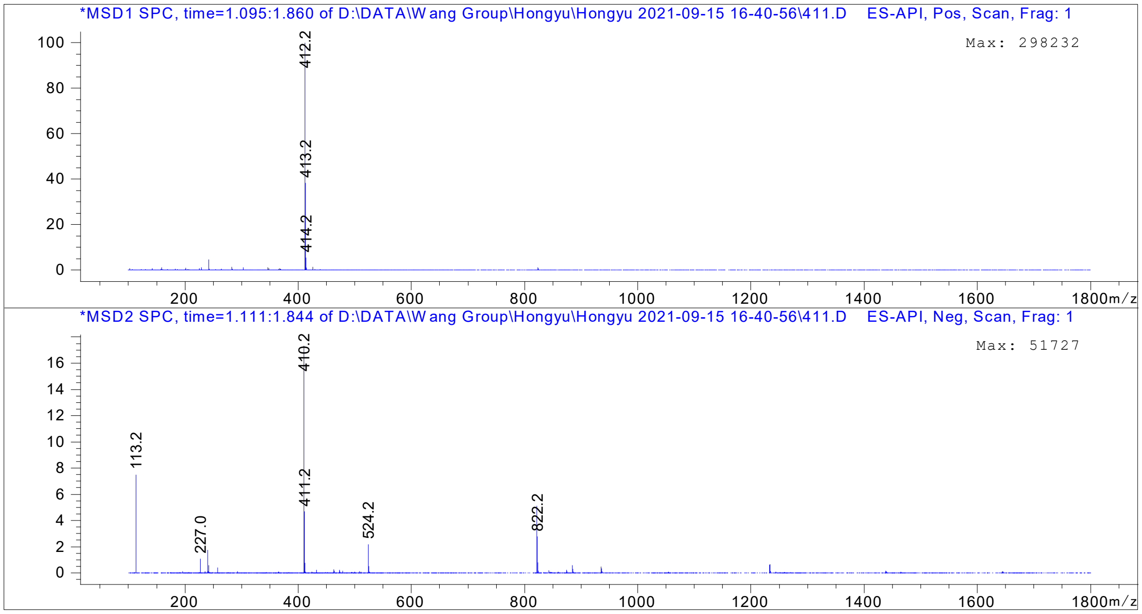

Supplementary Figure S74. (+) and (–)-ESI-MS of compound **22**.

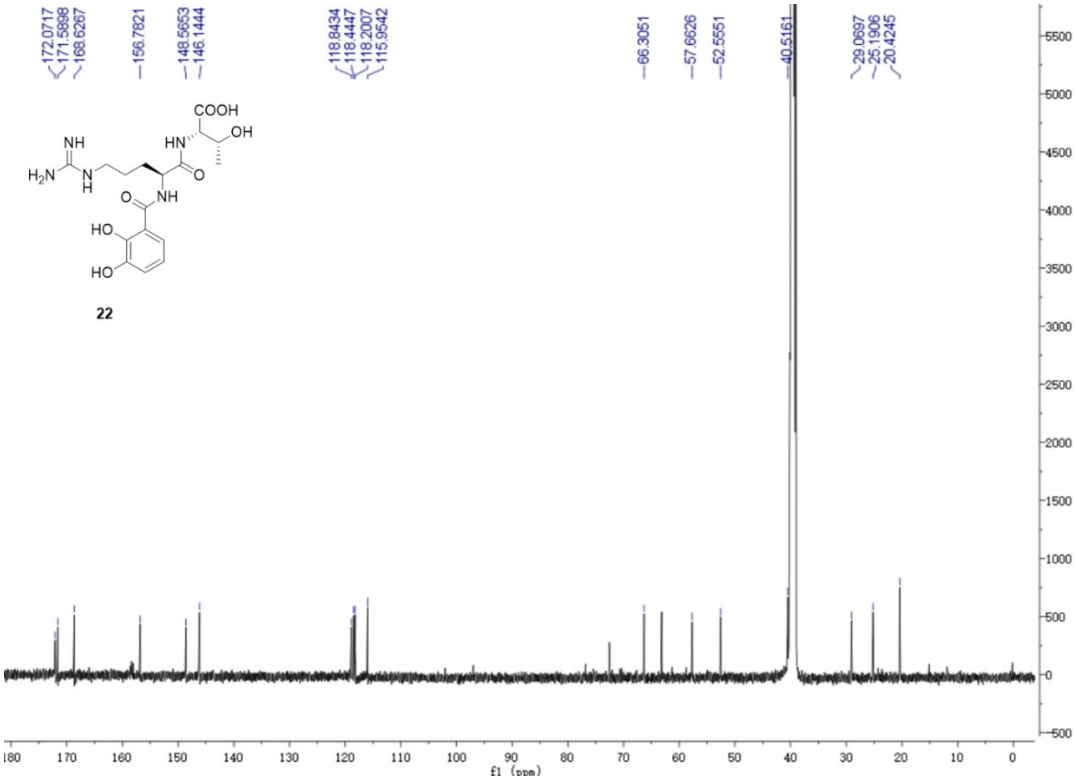

Supplementary Figure S75. ^{13}C NMR (125 MHz, DMSO-d_6) spectrum of compound **22**.

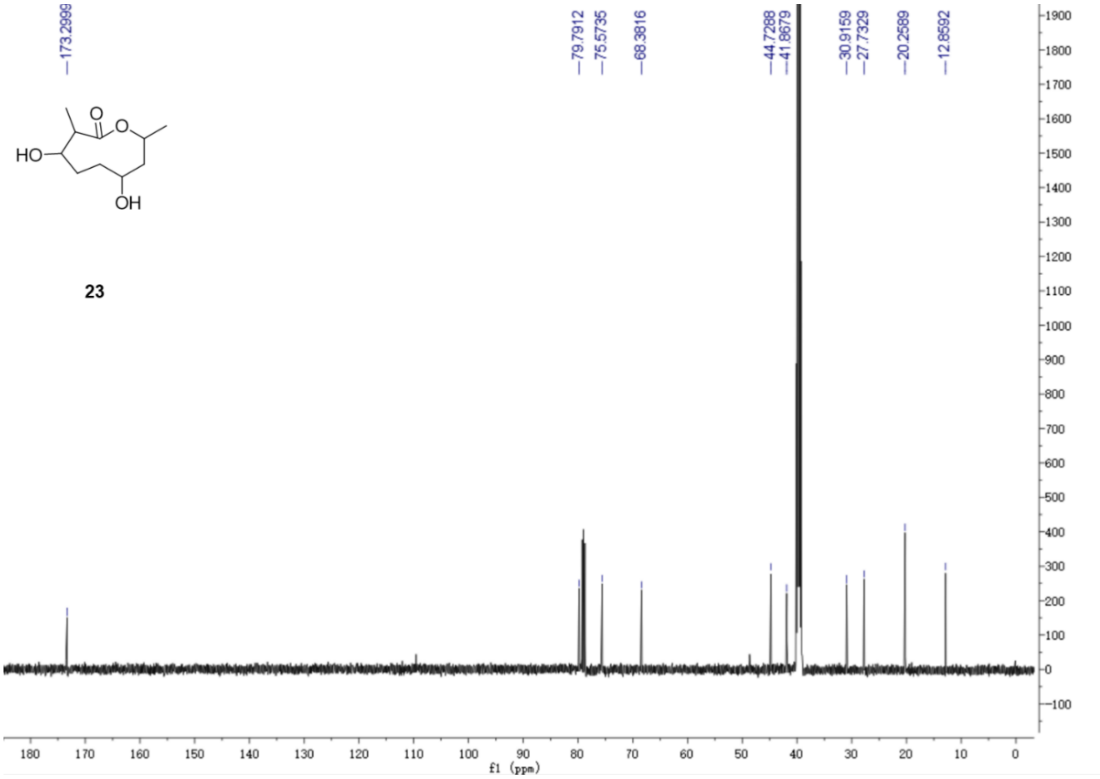

Supplementary Figure S76. ^{13}C NMR (125 MHz, DMSO-d_6) spectrum of compound **23**.

1.2 Supplementary Tables

Supplementary Table S1. Phylogenetic analyses of 124 endophytic strains from *Achyranthes bidentata*.

NO.	Strains	Parts	Closest sequences by BlastN	Percent Identity	Genbank accession
**1**	NX-R-1	Root	*Streptomyces triostinicus*	100.00%	OQ438164
**2**	NX-R-2	Root	*Streptomyces manipurensis*	99.93%	OQ438165
**3**	NX-R-3	Root	*Streptomyces* sp. CPE338	100.00%	OQ438166
**4**	NX-R-4	Root	*Streptomyces paradoxus*	96.61%	OQ438167
**5**	NX-R-5	Root	Same as 45	-	-
**6**	NX-R-6	Root	*Streptomyces flavogriseus*	99.93%	OQ438168
**7**	NX-R-7	Root	*Bacillus thuringiensis*	99.93%	OQ438169
**8**	NX-R-8	Root	*Streptomyces* sp.	98.04%	OQ438170
**9**	NX-R-9	Root	*Streptomyces phaeoverticillatus* subsp. *takatsukiensis*	98.24%	OQ438171
**10**	NX-R-10	Root	*Streptomyces collinus*	99.93%	OQ438172
**11**	NX-R-11	Root	*Streptomyces* sp.	99.86%	OQ438173
**12**	NX-R-12	Root	*Streptomyces* sp.	99.93%	OQ438174
**13**	NX-R-13	Root	*Streptomyces* sp. X2-21	99.93%	OQ438175
**14**	NX-R-14	Root	Same as 16	-	-
**15**	NX-R-15	Root	*Streptomyces* sp. MG62	99.72%	OQ438176
**16**	NX-R-16	Root	*Streptomyces sporovirgulis*	99.72%	OQ438177
**17**	NX-R-17	Root	*Streptomyces* sp.	95.07%	OQ438178
**18**	NX-R-18	Root	*Streptomyces* sp.	99.81%	OQ438179
**19**	NX-R-19	Root	*Streptomyces chartreusis*	96.33%	OQ438180
**20**	NX-R-20	Root	*Streptomyces cadmiisoli*	99.93%	OQ438181
**21**	NX-R-21	Root	*Bacillus anthracis*	100.00%	OQ438182
**22**	NX-R-22	Root	*Streptomyces* sp. RM395	100.00%	OQ438183
**23**	NX-R-23	Root	*Streptomyces phaeochromogenes*	99.93%	OQ438184
**24**	NX-R-24	Root	*Streptomyces halstedii*	99.79%	OQ438185
**25**	NX-R-25	Root	*Streptomyces* sp.	98.72%	OQ438186
**26**	NX-R-26	Root	*Streptomyces canus*	99.72%	OQ438187
**27**	NX-R-27	Root	*Streptomyces* sp. SOK6/25-05	99.93%	OQ438188
**28**	NX-R-28	Root	*Streptomyces canus*	99.93%	OQ438189
**29**	NX-R-29	Root	*Streptomyces* sp. SOK6/25-05	99.93%	OQ438190
**30**	NX-R-30	Root	*Actinobacteria* bacterium	100.00%	OQ438191
**31**	NX-R-31	Root	*Streptomyces canus*	99.86%	OQ438192
**32**	NX-R-32	Root	*Streptomyces* sp. GLY-P2	99.39%	OQ438193
**33**	NX-R-33	Root	*Streptomyces* sp.	97.43%	OQ438194
**34**	NX-R-34	Root	*Streptomyces parvus*	99.86%	OQ438195
**35**	NX-R-35	Root	*Streptomyces violascens*	100.00%	OQ438196
**36**	NX-R-36	Root	*Streptomyces flavogriseus*	99.93%	OQ438197
**37**	NX-R-37	Root	*Streptomyces* sp. Sn-13	99.86%	OQ438198
**38**	NX-R-38	Root	*Streptomyces flavogriseus*	99.93%	OQ438199
**39**	NX-R-39	Root	*Streptomyces canus*	100.00%	OQ438200
**40**	NX-R-40	Root	*Streptomyces beijiangensis*	99.81%	OQ438201
**41**	NX-R-41	Root	*Streptomyces phaeochromogenes*	99.71%	OQ438202
**42**	NX-R-42	Root	*Streptomyces* sp.	100.00%	OQ438203
**43**	NX-R-43	Root	*Streptomyces tauricus*	100.00%	OQ438204
**44**	NX-R-44	Root	*Streptomyces* sp. X1-2	99.66%	OQ438205
**45**	NX-R-45	Root	*Streptomyces* sp.	99.59%	OQ438206
**46**	NX-R-46	Root	*Rhodococcus* sp. 7-181	99.93%	OQ438207

**47**	NX-R-47	Root	*Streptomyces tauricus*	100.00%	OQ438208
**48**	NX-R-48	Root	*Bacillus thuringiensis*	99.93%	OQ438209
**49**	NX-R-49	Root	*Bacillus cereus*	99.93%	OQ438210
**50**	NX-R-50	Root	*Streptomyces parvus*	99.79%	OQ438211
**51**	NX-R-51	Root	*Streptomyces* sp.	99.37%	OQ438212
**52**	NX-R-52	Root	*Streptomyces* sp.	99.93%	OQ438213
**53**	NX-R-53	Root	*Streptomyces* sp.	100.00%	OQ438214
**54**	NX-R-54	Root	*Streptomyces* sp. SOK6/25-05	100.00%	OQ438215
**55**	NX-R-55	Root	*Streptomyces* sp.	99.93%	OQ438216
**56**	NX-R-56	Root	*Streptomyces manipurensis*	99.86%	OQ438217
**57**	NX-R-57	Root	*Streptomyces canus*	99.79%	OQ438218
**58**	NX-R-58	Root	*Streptomyces collinus*	100.00%	OQ438219
**59**	NX-R-59	Root	*Streptomyces microflavus*	99.86%	OQ438220
**60**	NX-R-60	Root	*Streptomyces anulatus*	99.93%	OQ438221
**61**	NX-R-61	Root	*Streptomyces manipurensis*	99.93%	OQ438222
**62**	NX-R-62	Root	*Streptomyces* sp.	100.00%	OQ438223
**63**	NX-R-63	Root	*Streptomyces* sp.	99.93%	OQ438224
**64**	NX-R-64	Root	*Streptomyces collinus*	100.00%	OQ438225
**65**	NX-R-65	Root	*Streptomyces* sp. CPE338	100.00%	OQ438226
**66**	NX-R-66	Root	*Streptomyces* sp. GLY-P2	99.86%	OQ438227
**67**	NX-R-67	Root	*Nocardia* sp. 30903	99.93%	OQ438228
**68**	NX-R-68	Root	*Streptomyces* sp. YRA166	98.94%	OQ438229
**69**	NX-R-69	Root	*Streptomyces* sp. A333 Ydz-QZ	100.00%	OQ438230
**70**	NX-R-70	Root	*Nocardia asteroides*	100.00%	OQ438231
**71**	NX-R-71	Root	*Bacillus cereus*	100.00%	OQ438232
**72**	NX-R-72	Root	*Streptomyces* sp. 0616206	99.86%	OQ438233
**73**	NX-R-73	Root	*Streptomyces neyagawaensis*	99.79%	OQ438234
**74**	NX-R-74	Root	*Streptomyces anulatus*	99.86%	OQ438235
**75**	NX-L-1	Leaf	*Streptomyces* sp. NBRC 3111	99.93%	OQ438236
**76**	NX-L-2	Leaf	*Streptomyces* sp. X4-1	99.64%	OQ438237
**77**	NX-L-3	Leaf	*Actinobacteria* bacterium	99.26%	OQ438238
**78**	NX-L-4	Leaf	*Streptomyces* sp. CPE314	97.31%	OQ438239
**79**	NX-L-5	Leaf	*Streptomyces* sp. BF-9	99.93%	OQ438240
**80**	NX-L-6	Leaf	*Streptomyces* sp. MM117	99.86%	OQ438241
**81**	NX-L-7	Leaf	*Streptomyces griseolus*	99.51%	OQ438242
**82**	NX-L-8	Leaf	*Streptomyces* sp.	99.93%	OQ438243
**83**	NX-L-9	Leaf	*Streptomyces* sp.	99.42%	OQ438244
**84**	NX-L-10	Leaf	*Streptomyces rubiginosohelvolus*	99.86%	OQ438245
**85**	NX-L-11	Leaf	*Streptomyces tricolor*	99.79%	OQ438246
**86**	NX-L-12	Leaf	*Streptomyces zaomyceticus*	99.93%	OQ438247
**87**	NX-L-13	Leaf	*Streptomyces parvus*	99.72%	OQ438248
**88**	NX-L-14	Leaf	*Streptomyces* sp.	98.65%	OQ438249
**89**	NX-L-15	Leaf	*Streptomyces* sp. QZGY-A35	99.79%	OQ438250
**90**	NX-L-16	Leaf	Same as 123	-	-
**91**	NX-L-17	Leaf	*Streptomyces variabilis*	100.00%	OQ438251
**92**	NX-L-18	Leaf	*Streptomyces zaomyceticus*	99.93%	OQ438252
**93**	NX-L-19	Leaf	*Nocardia asteroides*	100.00%	OQ438253
**94**	NX-L-20	Leaf	*Methylobacterium brachiatum*	99.90%	OQ438254
**95**	NX-L-21	Leaf	*Bacillus cereus*	99.16%	OQ438255
**96**	NX-L-22	Leaf	*Microbacterium* sp. PTS2402	94.75%	OQ438256
**97**	NX-L-23	Leaf	*Streptomyces* sp. STR43	98.75%	OQ438257
**98**	NX-L-24	Leaf	*Streptomyces* sp.	99.93%	OQ438258
**99**	NX-L-25	Leaf	*Streptomyces violascens*	100.00%	OQ438259

**100**	NX-L-26	Leaf	*Streptomyces* sp. A160 Ydz-ZZ	100.00%	OQ438260
**101**	NX-L-27	Leaf	*Streptomyces microflavus*	99.93%	OQ438261
**102**	NX-L-28	Leaf	*Streptomyces parvus*	99.93%	OQ438262
**103**	NX-L-29	Leaf	*Streptomyces halstedii*	99.79%	OQ438263
**104**	NX-L-30	Leaf	*Streptomyces akiyoshiensis*	99.93%	OQ438264
**105**	NX-L-31	Leaf	*Streptomyces* sp. MM14	99.86%	OQ438265
**106**	NX-S-1	Stem	*Streptomyces* sp. E5N187	99.86%	OQ438266
**107**	NX-S-2	Stem	*Streptomyces* sp. wa22	98.28%	OQ438267
**108**	NX-S-3	Stem	*Streptomyces venezuelae*	99.86%	OQ438268
**109**	NX-S-4	Stem	*Streptomyces* sp. X54	99.79%	OQ438269
**110**	NX-S-5	Stem	*Streptomyces halstedii*	99.23%	OQ438270
**111**	NX-S-6	Stem	*Penicillium sp.*	99.63%	OQ438028
**112**	NX-S-7	Stem	*Nocardia asteroides*	100.00%	OQ438271
**113**	NX-S-8	Stem	*Streptomyces anulatus*	99.86%	OQ438272
**114**	NX-S-9	Stem	*Streptomyces* sp. SM18	96.96%	OQ438273
**115**	NX-S-10	Stem	*Nocardia asteroides*	100.00%	OQ438274
**116**	NX-S-11	Stem	*Streptomyces* sp.	98.64%	OQ438275
**117**	NX-S-12	Stem	*Streptomyces* sp.	99.86%	OQ438276
**118**	NX-S-13	Stem	*Streptomyces flavogriseus*	100.00%	OQ438277
**119**	NX-S-14	Stem	*Nocardia asteroides*	100.00%	OQ438278
**120**	NX-S-15	Stem	*Streptomyces* sp. A2-16	99.86%	OQ438279
**121**	NX-S-16	Stem	*Nocardia asteroides*	100.00%	OQ438280
**122**	NX-S-17	Stem	*Streptomyces griseolus*	99.51%	OQ438281
**123**	NX-S-18	Stem	*Bacillus* sp. CSB5	83.70%	OQ438282
**124**	NX-AP-1	Aerial Part	*Bacillus cereus*	99.86%	OQ438283

(Red color: selected for further study).

Supplementary Table S2. Phylogenetic analyses of 13 endophytic strains from *Ainsliaea fragrans*.

NO.	Strains	Parts	Closest sequences by BlastN	Percent Identity	Genbank accession
**1**	XXTEF-R-1	Root	*Streptomyces anulatus*	99.93%	OQ438816
**2**	XXTEF-SL-1	Stem-Leaf	*Streptomyces* sp. INBio_4506AN	99.93%	OQ438817
**3**	XXTEF-SL-2	Stem-Leaf	*Streptomyces* sp. SM18	99.93%	OQ438818
**4**	XXTEF-SL-3	Stem-Leaf	*Streptomyces mucoflavus*	98.56%	OQ438819
**5**	XXTEF-SL-4	Stem-Leaf	*Streptomyces mucoflavus*	98.56%	OQ438820
**6**	XXTEF-SL-5	Stem-Leaf	*Streptomyces* sp. SM18	99.93%	OQ438821
**7**	XXTEF-SL-6	Stem-Leaf	*Streptomyces halstedii*	99.93%	OQ438822
**8**	XXTEF-SL-7	Stem-Leaf	*Streptomyces variabilis*	100.00%	OQ438823
**9**	XXTEF-SL-8	Stem-Leaf	*Streptomyces* sp. SM18	99.93%	OQ438824
**10**	XXTEF-SL-9	Stem-Leaf	*Streptomyces* sp. MJM4235	99.86%	OQ438825
**11**	XXTEF-SL-10	Stem-Leaf	*Streptomyces mucoflavus*	98.56%	OQ438826
**12**	XXTEF-F-1	Flower	*Micromonospora chokoriensis*	99.79%	OQ438827
**13**	XXTEF-F-2	Flower	*Bacillus thuringiensis*	99.93%	OQ438828

Supplementary Table S3. Phylogenetic analyses of 11 endophytic strains from *Artemisia capillaris*.

NO.	Strains	Parts	Closest sequences by BlastN	Percent Identity	Genbank accession
**1**	YCH-R-1	Root	*Streptomyces chartreusis* NRRL 3882	100.00%	OQ430664
**2**	YCH-L-1	Leaf	*Streptomyces zaomyceticus*	100.00%	OQ430665
**3**	YCH-AP-1	Aerial Part	*Microbacterium hydrocarbonoxydans*	99.93%	OQ430666
**4**	YCH-AP-2	Aerial Part	*Terribacillus goriensis*	100.00%	OQ430667
**5**	YCH-AP-3	Aerial Part	*Streptomyces* sp.	100.00%	OQ430668
**6**	YCH-AP-4	Aerial Part	*Streptomyces* sp. A311Ydz-AH	99.86%	OQ430669
**7**	YCH-AP-5	Aerial Part	*Staphylococcus xylosus*	99.93%	OQ430670
**8**	YCH-AP-6	Aerial Part	*Streptomyces* sp.	99.17%	OQ430671
**9**	YCH-AP-7	Aerial Part	*Streptomyces* sp. DUT_AHX	99.93%	OQ430672
**10**	YCH-AP-8	Aerial Part	*Streptomyces neopeptinius*	99.79%	OQ430673
**11**	YCH-AP-9	Aerial Part	*Streptomyces pseudovenezuelae*	99.93%	OQ430674

Supplementary Table S4. Phylogenetic analyses of 13 endophytic strains from *Daucus carota*.

**NO.**	**Strains**	**Parts**	**Closest sequences by BlastN**	**Percent Identity**	**Genbank accession**
**1**	YHLB-R-1	Root	*Streptomyces griseolus*	98.38%	OQ438148
**2**	YHLB-R-2	Root	*Streptomyces halstedii*	99.86%	OQ438149
**3**	YHLB-R-3	Root	*Streptomyces flavogriseus*	100.00%	OQ438150
**4**	YHLB-L-1	Leaf	*Methylobacterium aquaticum*	97.75%	OQ438151
**5**	YHLB-L-2	Leaf	*Streptomyces diastatochromogenes*	100.00%	OQ438152
**6**	YHLB-L-3	Leaf	*Microbacterium oxydans*	94.18%	OQ438153
**7**	YHLB-L-4	Leaf	*Sphingomonas* sp.	99.93%	OQ438154
**8**	YHLB-S-1	Stem	*Streptomyces flaveus*	99.93%	OQ438155
**9**	YHLB-S-2	Stem	*Streptomyces griseolus*	99.79%	OQ438156
**10**	YHLB-S-3	Stem	*Streptomyces flavogriseus*	100.00%	OQ438157
**11**	YHLB-F-1	Flower	*Kineococcus tegulae*	99.54%	OQ438158
**12**	YHLB-F-2	Flower	Same as 13	-	-
**13**	YHLB-F-3	Flower	*Methylobacterium* sp. N7	99.93%	OQ438159

(Red color: selected for further study).

Supplementary Table S5. Phylogenetic analyses of 21 endophytic strains from *Leonurus japonicus*.

**NO.**	**Strains**	**Parts**	**Closest sequences by BlastN**	**Percent Identity**	**Genbank accession**
**1**	YMC-R-1	Root	*Streptomyces globisporus*	99.93%	OQ438707
**2**	YMC-R-2	Root	*Bacillus albus*	99.93%	OQ438708
**3**	YMC-R-3	Root	*Streptomyces venezuelae*	99.72%	OQ438709
**4**	YMC-R-4	Root	*Microbacterium* sp. SuP-E064	99.64%	OQ438710
**5**	YMC-R-5	Root	*Streptomyces zaomyceticus*	99.79%	OQ438711
**6**	YMC-R-6	Root	*Streptomyces* sp. NJYH2618	99.86%	OQ438712
**7**	YMC-R-7	Root	*Streptomyces* sp. X1-2	98.86%	OQ438713
**8**	YMC-R-8	Root	*Streptomyces* sp.	99.38%	OQ438714
**9**	YMC-R-9	Root	*Streptomyces albogriseolus*	99.93%	OQ438715
**10**	YMC-R-10	Root	*Streptomyces albogriseolus*	100.00%	OQ438716
**11**	YMC-R-11	Root	*Bacillus cereus*	99.93%	OQ438717
**12**	YMC-SL-1	Stem-Leaf	*Methylorubrum populi*	97.96%	OQ438718
**13**	YMC-SL-2	Stem-Leaf	*Streptomyces lateritius*	99.86%	OQ438719
**14**	YMC-SL-3	Stem-Leaf	*Curtobacterium* sp. b163	100.00%	OQ438720
**15**	YMC-F-1	Flower	*Streptomyces* sp. HF-0	100.00%	OQ438721
**16**	YMC-F-2	Flower	*Streptomyces* sp. G49	99.03%	OQ438722
**17**	YMC-F-3	Flower	*Streptomyces* sp. 3076	99.79%	OQ438742
**18**	YMC-F-4	Flower	*Streptomyces* sp. FSRh8	99.86%	OQ438723
**19**	YMC-F-5	Flower	*Streptomyces griseolus*	99.93%	OQ438724
**20**	YMC-F-6	Flower	*Streptomyces* sp. SM18	99.86%	OQ438725
**21**	YMC-F-7	Flower	*Streptomyces* sp. SM18	99.79%	OQ438726

Supplementary Table S6. Phylogenetic analyses of 15 endophytic strains from *Perilla frutescens*.

NO.	Strains	Parts	Closest sequences by BlastN	Percent Identity	Genbank accession
**1**	BS-R-1	Root	*Streptomyces fragilis*	99.65%	OQ438801
**2**	BS-AP-1	Aerial-Part	*Streptomyces* sp.	99.79%	OQ438802
**3**	BS-AP-2	Aerial-Part	*Streptomyces* sp. JSM 147702	99.93%	OQ438803
**4**	BS-AP-3	Aerial-Part	*Streptomyces tanashiensis*	99.36%	OQ438804
**5**	BS-AP-4	Aerial-Part	*Streptomyces kunmingensis*	99.93%	OQ438805
**6**	BS-AP-5	Aerial-Part	*Streptomyces* sp.	99.57%	OQ438806
**7**	BS-AP-6	Aerial-Part	*Methylobacterium* sp. MG-2011-78-DC	99.86%	OQ438807
**8**	BS-AP-7	Aerial-Part	*Nakamurella* sp.	99.63%	OQ438808
**9**	BS-AP-8	Aerial-Part	*Nakamurella* sp.	99.92%	OQ438809
**10**	BS-AP-9	Aerial-Part	*Streptomyces kunmingensis*	99.79%	OQ438810
**11**	BS-1	Whole Plant	*Nocardia* sp. HBUM 79084	100.00%	OQ438811
**12**	BS-2	Whole Plant	*Streptomyces* sp. ACTYS11	99.93%	OQ438812
**13**	BS-3	Whole Plant	*Streptomyces* sp. FSRh6	99.86%	OQ438813
**14**	BS-4	Whole Plant	*Streptomyces akiyoshiensis*	99.52%	OQ438814
**15**	BS-5	Whole Plant	*Streptomyces* sp. JSM 147702	99.93%	OQ438815

Supplementary Table S7. Phylogenetic analyses of 28 endophytic strains from *Prunella vulgris*.

NO.	Strains	Parts	Closest sequences by BlastN	Percent Identity	Genbank accession
**1**	XKC-R-1	Root	*Streptomyces* sp. SM18	99.66%	OQ449506
**2**	XKC-R-2	Root	*Streptomyces* sp. W	99.93%	OQ449507
**3**	XKC-R-3	Root	*Streptomyces zaomyceticus*	99.93%	OQ449508
**4**	XKC-R-4	Root	*Streptomyces* sp. 18(2008)	99.93%	OQ449509
**5**	XKC-R-5	Root	*Streptomyces* sp. SM18	99.86%	OQ449510
**6**	XKC-R-6	Root	*Bacillus cereus*	99.86%	OQ449511
**7**	XKC-R-7	Root	*Streptomyces* sp. ZG634	99.72%	OQ449512
**8**	XKC-R-8	Root	*Streptomyces* sp. TY68-3	100.00%	OQ449519
**9**	XKC-R-9	Root	*Streptomyces* sp.	98.00%	OQ449513
**10**	XKC-R-10	Root	*Streptomyces canus*	96.09%	OQ449514
**11**	XKC-R-11	Root	*Streptomyces* lateritius	99.93%	OQ449515
**12**	XKC-R-12	Root	*Staphylococcus* sp. MRSA154B15_13_3E	100.00%	OQ449516
**13**	XKC-R-13	Root	*Streptomyces* sp. H	99.86%	OQ449517
**14**	XKC-R-14	Root	*Streptomyces parvus*	97.92%	OQ449518
**15**	XKC-R-15	Root	*Streptomyces* sp. H	99.93%	OQ449536
**16**	XKC-R-16	Root	*Streptomyces luteogriseus*	99.93%	OQ449552
**17**	XKC-R-17	Root	*Streptomyces* sp. TY68-3	100.00%	OQ449519
**18**	XKC-R-18	Root	*Streptomyces hygroscopicus*	99.86%	OQ449520
**19**	XKC-R-19	Root	*Streptomyces* sp.	99.15%	OQ449521
**20**	XKC-R-20	Root	*Streptomyces flavovariabilis*	99.86%	OQ449522
**21**	XKC-SL-1	Stem-Leaf	*Streptomyces* sp.	97.88%	OQ449523
**22**	XKC-SL-2	Stem--Leaf	*Kocuria* sp.	100.00%	OQ449524
**23**	XKC-SL-3	Stem-Leaf	*Microbacterium* sp. HP4S	100.00%	OQ449525
**24**	XKC-SL-4	Stem-Leaf	*Methylobacterium* sp. N7	99.93%	OQ449526
**25**	XKC-SL-5	Stem-Leaf	*Streptomyces sannanensis*	99.65%	OQ449527
**26**	XKC-F-1	Flower	*Pseudomonas parafulva*	99.86%	OQ449503
**27**	XKC-F-2	Flower	*Methylobacterium* sp. PB280	99.93%	OQ449504
**28**	XKC-F-3	Flower	*Streptomyces* sp. SM18	99.86%	OQ449505

Supplementary Table S8. Phylogenetic analyses of 16 endophytic strains from *Salvia miltiorrhiza*.

NO.	Strains	Parts	Closest sequences by BlastN	Percent Identity	Genbank accession
**1**	DS-R-1	Root	*Nocardia tengchongensis*	99.93%	OQ438792
**2**	DS-SL-1	Stem-Leaf	*Streptomyces* sp. E5N142	98.27%	OQ438769
**3**	DS-SL-2	Stem-Leaf	*Streptomyces caviscabies*	99.79%	OQ438770
**4**	DS-SL-3	Stem-Leaf	*Bosea* sp.	99.93%	OQ438771
**5**	DS-SL-4	Stem-Leaf	*Bacillus subtilis*	99.86%	OQ438772
**6**	DS-SL-5	Stem-Leaf	*Kocuria carniphila*	99.93%	OQ438773
**7**	DS-SL-6	Stem-Leaf	*Streptomyces* sp.	99.86%	OQ438774
**8**	DS-SL-7	Stem-Leaf	*Streptomyces glauciniger*	99.93%	OQ438775
**9**	DS-SL-8	Stem-Leaf	*Nocardia* sp. HBUM 79084	100.00%	OQ438776
**10**	DS-SL-9	Stem-Leaf	*Streptomyces verne*	99.86%	OQ438777
**11**	DS-SL-10	Stem-Leaf	*Streptomyces sannanensis*	99.79%	OQ438778
**12**	DS-SL-11	Stem-Leaf	*Streptomyces* sp. NBRC 3111	99.79%	OQ438779
**13**	DS-F-1	Flower	*Actinomycetia* bacterium	100.00%	OQ438780
**14**	DS-F-2	Flower	*Nocardia* sp. HBUM 79084	100.00%	OQ438781
**15**	DS-F-3	Flower	*Microbacterium saperdae*	99.44%	OQ438782
**16**	DS-F-4	Flower	*Micromonospora aurantiaca*	100.00%	OQ438783

Supplementary Table S9. Phylogenetic analyses of 28 endophytic strains from *Stemona sessilifolia*.

NO.	Strains	Parts	Closest sequences by BlastN	Percent Identity	Genbank accession
**1**	ZLBB-RT-1	Root-Tuber	*Streptomyces* sp. KGS-6-31	99.85%	OQ438618
**2**	ZLBB-RT-2	Root-Tuber	*Streptomyces pseudovenezuelae*	99.64%	OQ438619
**3**	ZLBB-RT-3	Root-Tuber	*Streptomyces diastatochromogenes*	99.57%	OQ438620
**4**	ZLBB-RT-4	Root-Tuber	*Streptomyces microsporus*	99.79%	OQ438621
**5**	ZLBB-RT-5	Root-Tuber	*Streptomyces* sp. STR30	99.86%	OQ438622
**6**	ZLBB-RT-6	Root-Tuber	Same as 9	-	-
**7**	ZLBB-RT-7	Root-Tuber	*Staphylococcus xylosus*	99.93%	OQ438623
**8**	ZLBB-RT-8	Root-Tuber	*Nocardia* sp. FXJ1.491	100.00%	OQ438624
**9**	ZLBB-RT-9	Root-Tuber	*Nocardia niigatensis*	99.85%	OQ438625
**10**	ZLBB-L-1	Leaf	*Streptomyces* sp. A46Ydz-XG	99.93%	OQ438626
**11**	ZLBB-L-2	Leaf	*Streptomyces* sp. INBio_4515Q	98.78%	OQ438627
**12**	ZLBB-L-3	Leaf	*Streptomyces griseoruber*	97.10%	OQ438628
**13**	ZLBB-L-4	Leaf	*Micromonospora* sp. A6-9	100.00%	OQ438629
**14**	ZLBB-L-5	Leaf	*Streptomyces* sp.	99.79%	OQ438630
**15**	ZLBB-L-6	Leaf	*Streptomyces* sp. BF-9	99.72%	OQ438631
**16**	ZLBB-S-1	Stem	*Streptomyces tauricus*	100.00%	OQ438632
**17**	ZLBB-S-2	Stem	*Streptomyces flavofungini*	99.86%	OQ438633
**18**	ZLBB-S-3	Stem	*Streptomyces* sp. A240 Ydz-QZ	99.93%	OQ438634
**19**	ZLBB-S-4	Stem	*Streptomyces thinghirensis*	100.00%	OQ438635
**20**	ZLBB-S-5	Stem	*Streptomyces* sp. CLI2509	99.93%	OQ438636
**21**	ZLBB-S-6	Stem	*Streptomyces rubiginosohelvolus*	100.00%	OQ438637
**22**	ZLBB-S-7	Stem	*Streptomyces* sp.	99.93%	OQ438638
**23**	ZLBB-S-8	Stem	*Hyphomicrobium* sp.	98.98%	OQ438639
**24**	ZLBB-S-9	Stem	*Streptomyces* sp. RI104-LiC104	99.49%	OQ438640
**25**	ZLBB-S-10	Stem	*Streptomyces* sp. RI104-LiC104	99.31%	OQ438641
**26**	ZLBB-S-11	Stem	*Nocardia niigatensis*	98.00%	OQ438642
**27**	ZLBB-S-12	Stem	*Micromonospora* sp. A6-9	100.00%	OQ438643
**28**	ZLBB-S-13	Stem	*Nocardia niigatensis*	99.57%	OQ438644

(Red color: selected for further study).

Supplementary Table S10. Experimental and calculated ^{13}C NMR for **1** in $CDCl_3$.

**1**		6*S*-**1**		6*R*-**1**	
No.	δ_{exp}	δ_{cal}	$\Delta\delta$	δ_{cal}	$\Delta\delta$
1	196.5	198.5	-2.0	199.5	-3.0
2	103.4	105.5	-2.1	108.5	-5.1
3	187.9	183.5	4.4	180.5	7.4
4	97.3	99.8	-2.5	102.8	-5.5
5	163.5	159.8	3.7	158.8	4.7
6	75.0	74.9	0.1	74.9	0.1
7	7.6	6.8	0.8	6.1	1.5
8	32.8	30.7	2.1	28.7	4.1
1'	185.1	180.7	4.4	179.7	5.4
2'	123.8	128.5	-4.7	128.6	-4.8
3'	144.1	140.6	3.5	140.6	3.5
4'	131.2	132.9	-1.7	132.9	-1.7
5'	140.4	143.6	-3.2	143.6	-3.2
6'	18.9	17.2	1.7	17.2	1.7

Supplementary Table S11. Antibacterial screening results of three selected endophytes (inhibition, %).

Strains	Organisms	
	S. aureus	*E. coli*
NX-S-6	64	26
YHLB-L-2	51	72
ZLBB-S-6	97	10

1.3 Supplementary Schemes

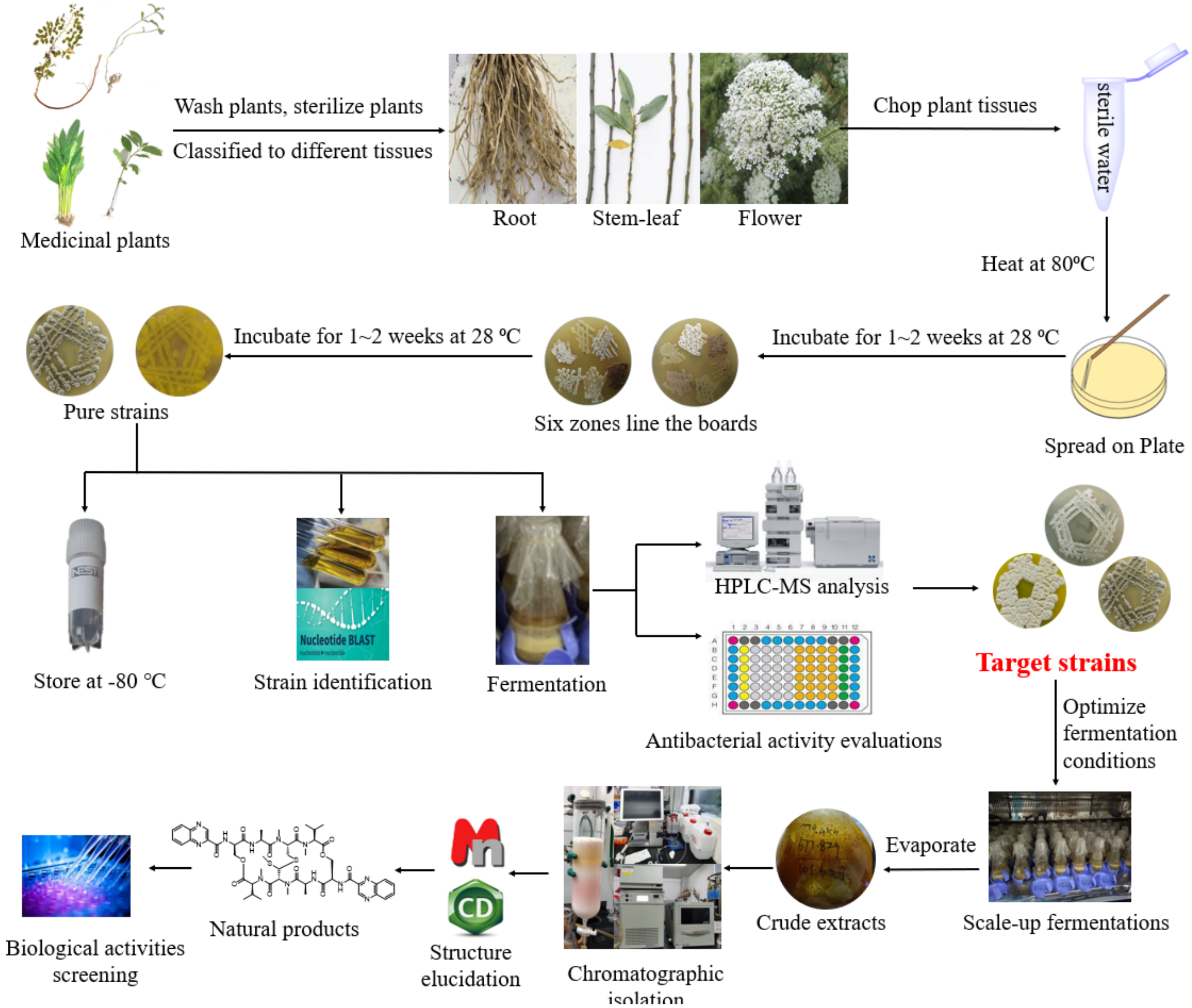

Supplementary Scheme S1. Working scheme for this project.

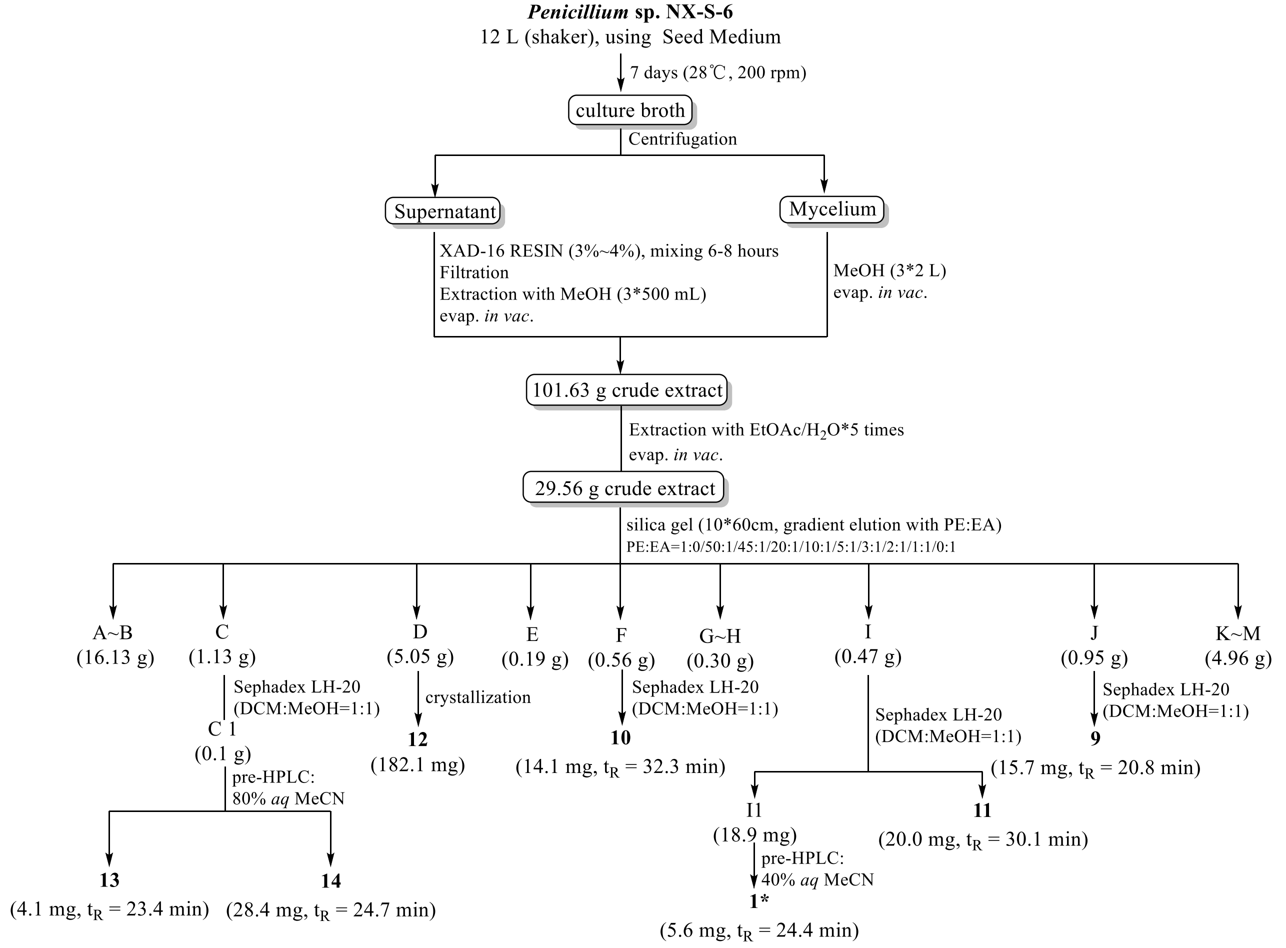

Supplementary Scheme S2. Work-up scheme for the *Penicillium sp.* NX-S-6 culture (1st).

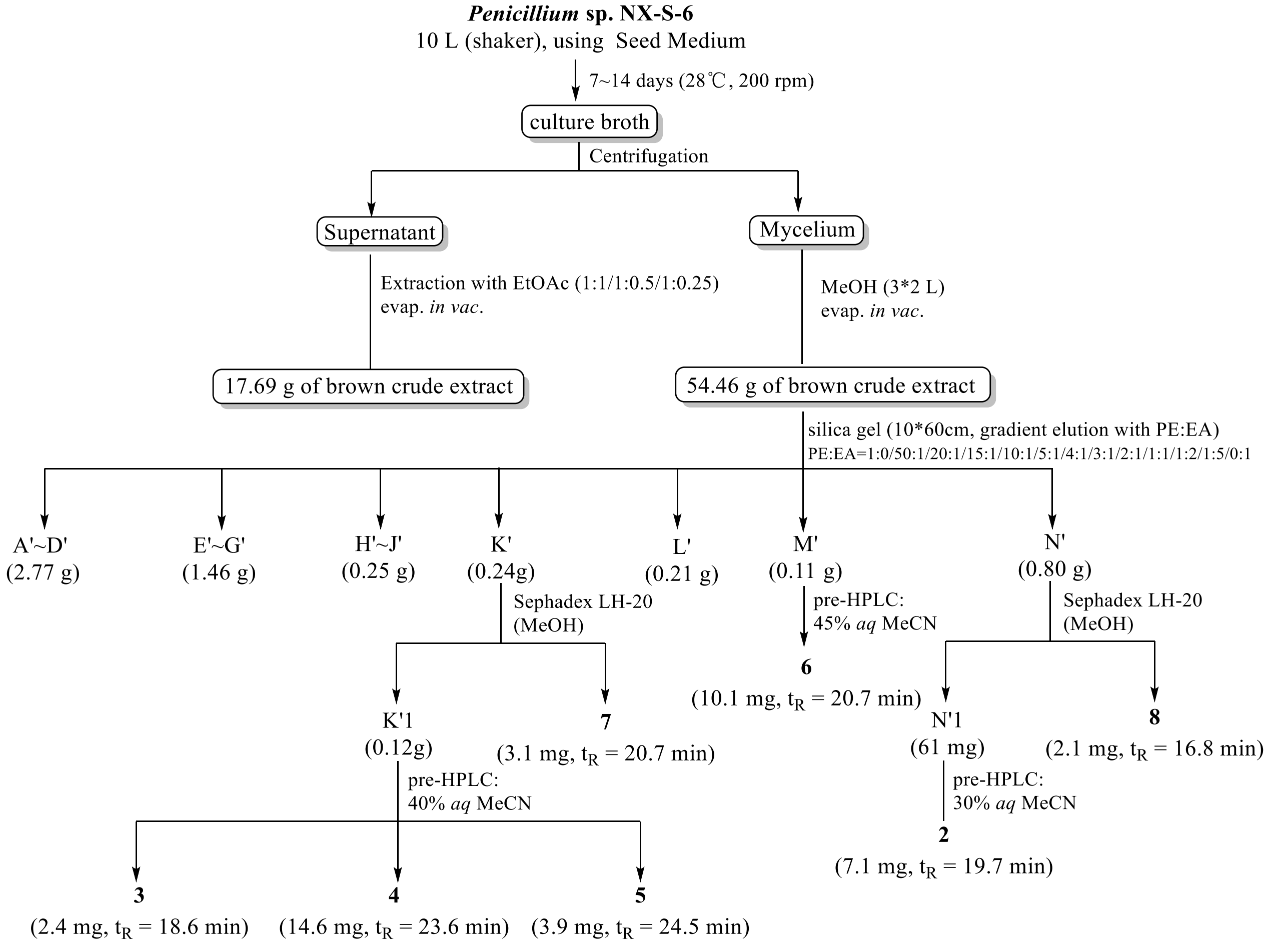

Supplementary Scheme S3. Work-up scheme for the *Penicillium sp.* NX-S-6 culture (2nd).

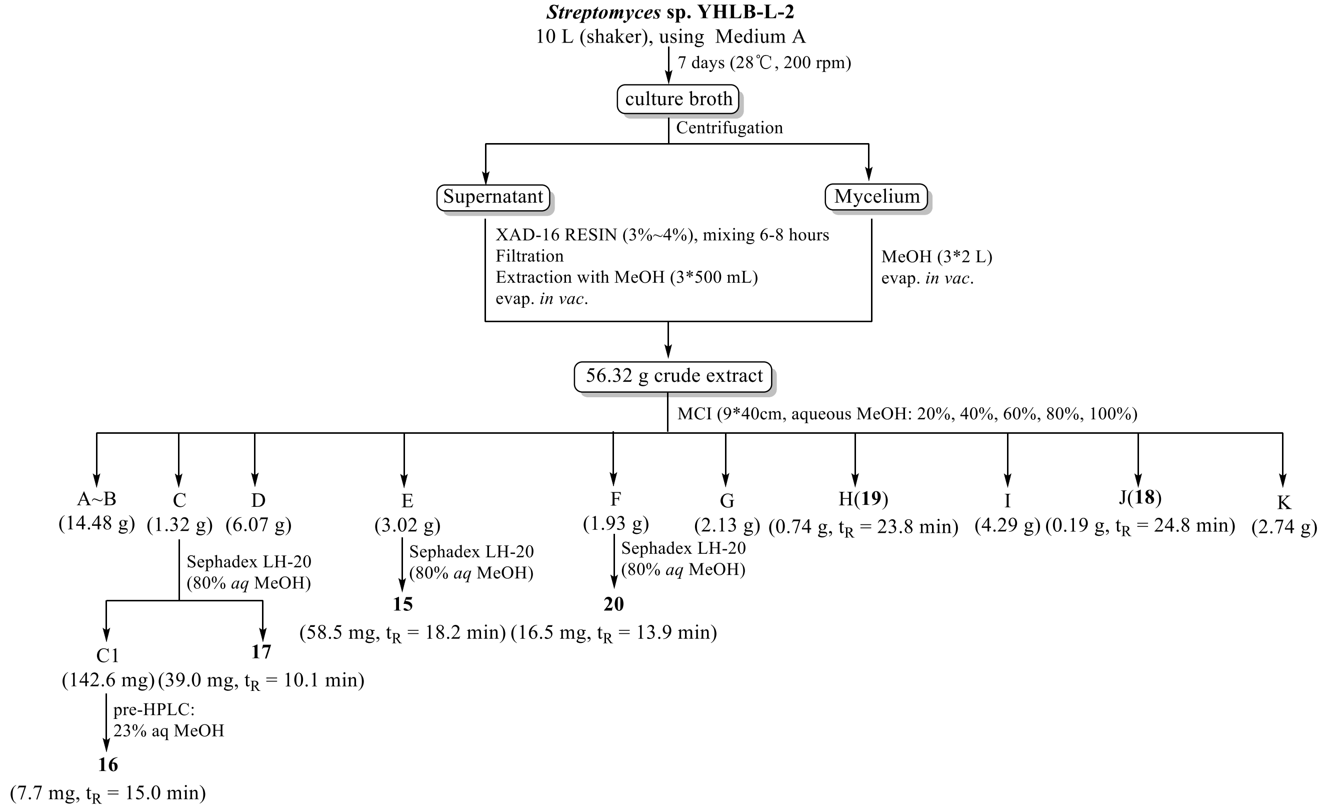

Supplementary Scheme S4. Work-up scheme for the *Streptomyces sp.* YHLB-L-2 culture.

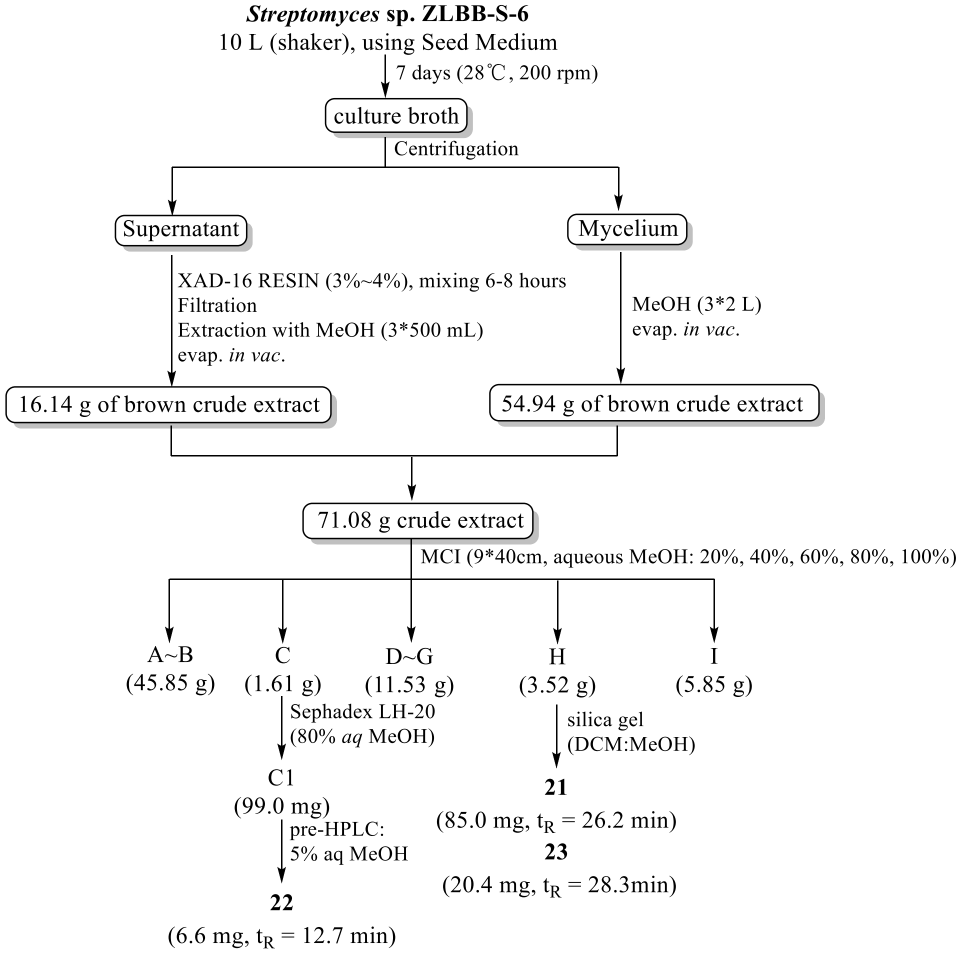

Supplementary Scheme S5. Work-up scheme for the *Streptomyces sp.* ZLBB-S-6 culture.
